# Multifaceted Strategy for the Synthesis of Diverse 2,2'-Bithiophene Derivatives

**DOI:** 10.3390/molecules20034565

**Published:** 2015-03-12

**Authors:** Stanisław Krompiec, Michał Filapek, Iwona Grudzka-Flak, Aneta Slodek, Sławomir Kula, Jan Grzegorz Malecki, Joanna Malarz, Grażyna Szafraniec-Gorol, Mateusz Penkala, Ewa Schab-Balcerzak, Marian Paluch, Michał Mierzwa, Marek Matussek, Agata Szlapa, Michał Pajak, Dariusz Blach, Beata Marcol, Witold Danikiewicz, Bartosz Boharewicz, Agnieszka Iwan

**Affiliations:** 1 Institute of Chemistry, Faculty of Mathematics, Physics and Chemistry, University of Silesia, Szkolna 9, Katowice 40-007, Poland; E-Mails: filus.lc@interia.pl (M.F.); iwonagrudzka@gmail.com (I.G.-F.); a.slodek@wp.pl (A.S.); kula.slawek@gmail.com (S.K.); gmalecki@us.edu.pl (J.G.M.); joanna.malarz@gmail.com (J.M.); grazyna.szafraniec@wp.pl (G.S.-G.); mateusz.penkala@us.edu.pl (M.P.); ewa.schab-balcerzak@us.edu.pl (E.S.-B.); marmat89@interia.pl (M.M.); agataszlapa@o2.pl (A.S.); spider1989@gmail.com (M.P.); dariusz2lo@o2.pl (D.B.); beata_marcol@wp.pl (B.M.); 2 Institute of Physics, Faculty of Mathematics, Physics and Chemistry, University of Silesia, Uniwersytecka 4, Katowice 40-007, Poland; E-Mails: marian.paluch@us.edu.pl (M.P.); michal.mierzwa@us.edu.pl (M.M.); 3 Polish Academy of Science, Institute of Organic Chemistry Kasprzaka 44/52, PO Box 58, Warsaw 01-224, Poland; E-Mail: witold.danikiewicz@icho.edu.pl; 4 Electrotechnical Institute, Division of Electrotechnology and Materials Science, M. Skłodowskiej-Curie 55/61, Wrocław 50-369, Poland; E-Mails: b.boharewicz@iel.wroc.pl (B.B.); a.iwan@iel.wroc.pl (A.I.)

**Keywords:** bithiophene derivatives, coupling, cycloaddition, high pressure, homogeneous catalysis, isomerization, PTC

## Abstract

New catalytically or high pressure activated reactions and routes, including coupling, double bond migration in allylic systems, and various types of cycloaddition and dihydroamination have been used for the synthesis of novel bithiophene derivatives. Thanks to the abovementioned reactions and routes combined with non-catalytic ones, new acetylene, butadiyne, isoxazole, 1,2,3-triazole, pyrrole, benzene, and fluoranthene derivatives with one, two or six bithiophenyl moieties have been obtained. Basic sources of crucial substrates which include bithiophene motif for catalytic reactions were 2,2'-bithiophene, gaseous acetylene and 1,3-butadiyne.

## 1. Introduction

Compounds containing thiophene, bithiophene or oligothiophene motifs are particularly popular because they are used in various fields of science and technology, ranging from organic chemistry and synthesis to material science, technology, medicine and pharmaceutical science. Therefore, new structures containing the abovementioned motifs are still being synthesized and the possibilities of their practical application intensively tested. Various organic and organometallic systems containing thiophene, bithiophene or oligothiophene moieties (and other essential structural elements, *i.e*., highly conjugated aromatic and heteroaromatic systems, coordinated metal centers, diimide or triphenylamine moiety and others) are used in organic electronics [1,2,3,4] and are still intensively investigated in OLED [1,2,3,4], organic field-effect transistor [1,5] and solar cell [1,6,7] technology.

As far as examples of application in medicine and pharmaceutical science are concerned, 5-(4-hydroxyphenyl)-5'-dicyanoethenyl-2,2'-bithiophene was tested as a marker of cell lesions in Alzheimer’s disease [8]. Moreover, 5'-mercapto-2,2'-bithiophene-5-carboxylic acid was tested as a potential biosensor detecting antigen-antibody connection [9], and a series of 5-(*E*)-styryl-2,2'-bithiophenes were tested as potential β-amyloid probes [10]. The starting substances for the synthesis of these compounds are simple derivatives of thiophene, bithiophene or oligothiophene substituted by Li, Br, I, C≡CH, MgCl, SnBu_3_, B(OH)_2_, B(OR)_2_, or ZnCl, which facilitate the chemical modification of their structures. The bithiophene derivatives, such as bromo- [11,12] and iodobithiophene [13,14,15], 5-trialkylstannyl-2,2'-bithiophene [16], 5-ethynyl-2,2'-bithiophene [17], 2,2'-bithiophene-5-boronic acid and its esters [12], and (2,2'-bithiophen-5-yl)magnesium bromide, are frequently used [18]. The abovementioned thiophene derivatives were obtained in the catalytic processes, mostly with the aid of transition metal catalysts.

However, in many cases such catalytic systems are inactive due to strong coordination of the substrate by a transition metal catalytic center [19]. This fact ought to be taken into consideration when planning catalytic systems to be used for transformations of strongly coordinated reagents, for instance thiophene or oligothiophene [19]. There are a number of stable neutral and cationic transition metal (Cr, Ru, Ir, Rh, Mn, Re, Ti, Cu, Fe *etc.*) complexes with thiophene. The issue of transition metal complexation with thiophene and oligothiophenes has been intensively studied in view of the hydrodesulfurization (HDS) process [20]. Thiophene coordinates transition metals in many ways, but the most common are the η^1^-S, η^1^-C, and η^5^- coordination modes [21,22], and furthermore, it forms neutral complexes with Re and Mn, involving C-, S- and π-coordinated ligands [23]. Moreover, it forms stable cationic complexes, e.g., [Mn(CO)_3_(η^5^-thiophene)][BF_4_], which precursor is [Mn(CO)_5_Br]. In addition, exchange of CO-ligand to η^5^-thiophene in the reaction of [Cr(CO)_6_] with various thiophene derivatives [Cr(CO)_3_(η^5^-substituted-thiophenes)] took place [24]. Furthermore, reaction of [RuCl_2_(*p*-cymene)]_2_ with tetramethylthiophene resulted in a very stable [(tetramethyl-thiophene)RuCl_2_]_2_ complex, indicating that thiophene coordinates a Ru atom stronger than *p*-cymene [25]. The above analysis indicates that strong coordination of a transition metal with thiophene and oligothiophene may interfere with some reactions involving substrates containing a thiophene moiety.

**Scheme 1:**
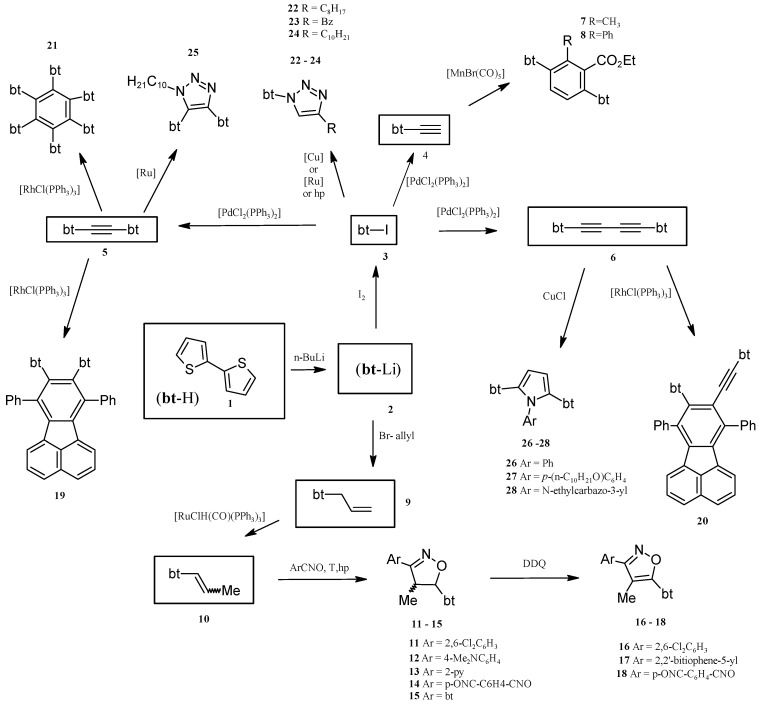
Synthetic pathways to various 2,2'-bithiophene derivatives from 2,2'-bithiophene (bt-H). Compounds marked as **7**, **8**, **10**–**28** are new.

The synthesis of pyridine [26], bipyridine, terpyridine [27], carbazole [28] and benzene [29] derivatives containing 2-thienyl, 3,4-methylenedioxothien-2-yl and/or 2,2'-bithiophen-5-yl moieties in Stille cross-coupling reactions has been recently reported. Herein, we present a novel strategy for the synthesis of many bithiophene derivatives via new catalytically or high pressure activated reactions and catalytic routes combined with non-catalytic ones (Scheme 1).

The substrates containing a bithiophene motif were prepared from commercially available 2,2'-bithiophene, which during the first stage was lithiated according to the procedure developed by us (Scheme 1). Next, the 5-lithio-2,2'-bithiophene (“the first generation” substrate) was converted into 5-iodo- and 5-allyl-2,2'-bithiophenes and *in situ* into 5-azido-2,2'-bithiophene (“the second generation” substrates). In the subsequent step, the abovementioned compounds were catalytically transformed into 5-ethynyl-2,2'-bithiophene, 1,2-bis(2,2'-bithiophene-5-yl)acetylene, 5-(1-propenyl)-2,2'-bithiophene, and 1,4-bis(2,2'-bithiophene-5-yl)buta-1,3-diyne (“the third generation” substrates).

It is worth noting that the reactions leading to new 2,2'-bithiophene derivatives have not been reported so far (or are significantly improved). Since our work is a multifaceted one, *i.e*., it concerns a series of different reactions, relevant literature is presented in each subsection.

## 2. Results and Discussion

### 2.1. Synthesis of Substrates (for Catalytic Reactions) Containing 2,2'-Bithiophene Moiety from 2,2'-Bithiophene

The initial step for the strategy presented in Scheme 1 was selective synthesis of 5-lithio-2,2'-bithiophene (**2**). Compound **2** was then used for a direct or multi-step preparation of the following compounds: 5-iodobithiophene (**3**), 5-ethynyl-2,2'-bithiophene (**4**), 1,2-bis(2,2'-bithiophen-5-yl)ethyne (**5**), and 1,4-bis(2,2'-bithiophen-5-yl)-1,3-butadiyne (**6**), 5-allyl-2,2'-bithiophene (**9**), 5-(1-propenyl)-2,2'-bithiophene (**10**) The selective synthesis of **2** via reaction of 2,2'-bithiophene with *n*-BuLi, and then synthesis of **3** and **4** was reported in detail in our previous paper [29] and patents [30,31]. Despite the fact that the synthesis of 5-iodo-2,2'-bithiophene (**3**) was highly selective and less than 0.5% of 5,5'-diiodo-2,2'-bithiophene was obtained, it was possible to obtain monocrystals of 5,5'-diiodo-2,2'-bithiophene which structure was determined by X-ray diffraction (Supplementary Table S3 in Supporting Information). From the practical point of view, it is very important that any bithiophene lithiation product used for the synthesis of **3**, **4**, **5**, **6**, **9** and **10**, contains 80% of **2**, less than 0.5% of dilithiobithiophene, and ~19.5% unreacted bithiophene. Low concentration of the dilithioderivative was crucial for the selectivity and yields of subsequent reactions, including the catalytic ones. In particular, thanks to the abovementioned selectivity, the formation of polymeric products was not observed in practice. Additionally, unreacted bithiophene turned out to be inert, did not disturb catalytic reactions, and was finally recovered in the pure product separation procedure.

**Scheme 2:**
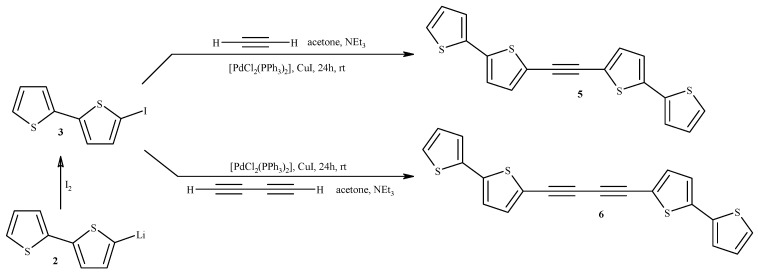
Synthesis of 5-iodo-2,2'-bithiophene (**3**), 1,2-bis(2,2'-bithiophen-5-yl)ethyne (**5**), and 1,4-bis(2,2'-bithiophen-5-yl)-1,3-butadiyne (**6**). The generation of buta-1,3-diyne has been previously described [11].

Compounds **5** and **6** were synthesized from gaseous acetylene or gaseous butadiyne using new methods (Scheme 2) [14,15]. Previously, ArC≡CAr type compounds, including 1,2-bis(2-thienyl)-acetylene, were obtained [32] using acetylene generated *in situ* from CaC_2_ and H_2_O in MeCN [11]. Compound **5** was previously obtained via three-step synthesis from iodobithiophene (coupling of **3** with TMS, distillation to **4** and finally coupling **3** with **4**) [33]. Compound **6** was synthesized via homocoupling of **4** in the presence of CuCl/TMEDA [34]. Additionally, various ArC≡CC≡CAr type compounds (but not **6**) were synthesized from ArI via Sonogashira-Glaser coupling [35]. On the other hand, the synthesis of compounds ArC≡CC≡CAr type from gaseous butadiyne is unknown.

The strategy of synthesis of some bithiophene-motif containing substrates from gaseous acetylene or gaseous butadiyne corresponds well with observed butadiyne rebirth in chemical technology [36]. It is well known that butadiyne is created as a side product of acetylene production [36]. Due to this fact, the application of the both gaseous reagents in our strategy, instead of protected forms of these compounds, is rational and modern. Additionally, commercially available trimethylsilylacetylene used by us for the synthesis of 5-ethynyl-2,2'-bithiophene was also prepared from acetylene [37].

In order to improve the yield of **5** and **6** we applied a cascade consisting of three reactors. The reactions were carried out in acetone due to a high solubility of acetylene and butadiyne therein. Other authors have used MeCN as solvent [32], but MeCN appears to be a much worse solvent (the solubility of acetylene therein is much lower), and in addition it is toxic. Gaseous acetylene (generated from CaC_2_ [34] or more conveniently supplied from a bottle gas) and butadiyne (generated from 1,4-dichloro-2-butyne) were dried before the introduction into the reaction system and additionally dispersed with a stream of argon. It is of particular importance in the case of butadiyne for safety reasons (high concentrated butadiyne is explosive). Moreover, in the procedure leading to **5** [PdCl_2_(PPh_3_)_2_] was applied, which was much more effective than Pd(OAc)_2_ + PPh_3_ catalytic system used by Chuentragool *et al.* in the synthesis of 1,2-bis(2-thienyl)acetylene [32]. Compound **6** was obtained as yellow crystals and its structure was confirmed using X-ray crystallography (Table 2 in Supporting Information). The [PdCl_2_(PPh_3_)_2_]/CuI catalytic system appeared to be more effective and convenient (due to its stability and price) than [Pd(PPh_3_)_4_] used by McCormick *et al*., in the synthesis of different ArC≡CC≡CAr from 1,4-bis(trimethylsilyl)-1,3-butadiyne [38].

**Scheme 3:**
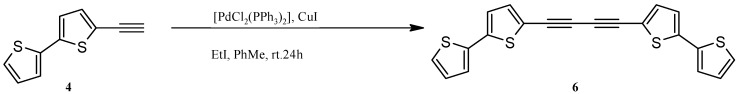
Homocoupling of 5-ethynyl-2,2'-bithiophene (**4**) to 1,4-bis(2,2'-bithiophen-5-yl)-buta-1,3-diyne (**6**) in the presence of ethyl iodide mediated by CuI/[PdCl_2_(PPh_3_)_2_].

Additionally, a new method of preparation of **6** via homocoupling of 5-ethynyl-2,2'-bithiophene (**4**) in the presence of ethyl iodide, catalyzed by CuI/[PdCl_2_(PPh_3_)_2_] was developed (Scheme 3). This method turned out to be very simple and effective (isolated yield was 97%).

It was found that the absence of alkyl iodide or bromide resulted in extremely low yield (approximately 10%). In addition, the influence of different alkyl bromides and iodides on the yield of this reaction was tested. It was confirmed that the yield of homocoupling depends on the type of the halide as follows: decyl bromide (60%), ethyl bromide (70%), decyl iodide (80%), and ethyl iodide (97%). Moreover, no heterocoupled products of reaction of alkyl halides with 5-ethynyl-2,2'-bithiophene (to bt-≡-R) were observed.

### 2.2. Benzene Derivatives with Two bt Moieties Prepared via Manganese-Catalyzed Reaction

**Scheme 4:**
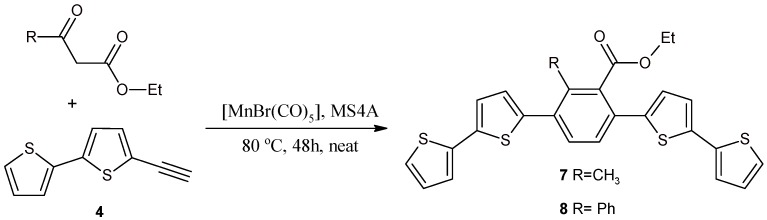
Synthesis of 1,4-bis(2,2'-bithiophen-5-yl)-2-methyl-3-ethoxycarbonylbenzene (**7**) and 1,4-bis(2,2'-bithiophen-5-yl)-2-phenyl-3-ethoxycarbonylbenzene (**8**) via manganese-catalyzed reactions of acetyl- or benzoylacetate with 5-ethynyl-2,2'-bithiophene (**4**).

We synthesized 1,4-bis(2,2'-bithiophen-5-yl)-2-methyl-3-etoxycarbonylobenzene (**7**) and 1,4-bis(2,2'-bithiophen-5-yl)-2-phenyl-3-etoxycarbonylobenzene (**8**) from **4** and ethyl acetoacetate or benzoylacetate via [MnBr(CO)_5_]-catalyzed dehydrative [2 + 2 + 2] cycloaddition (Scheme 4).

The manganese-catalyzed dehydrative [2 + 2 + 2] cycloaddition of 1,3-dicarbonyl compounds to terminal alkynes leading to 2,3,6-trisubstituted methyl or ethyl benzoate is known from the literature. However, heteroaromatic substituents are unknown [39,40,41]. A slight modification of this method allowed us to obtain compounds **7** and **8** with electron-rich heteroaromatic substituents such a 2,2'-bithiophen-5-yl. We did not observe any desulfurization side reactions or other transformations of the bithiophene systems, even though the catalytic reactions (using Mn, Pd, Rh, Ru, and Cu complexes) were carried out at high temperatures. However, the procedures applied by us for the synthesis of **7** and **8** did not cause the deactivation of the catalytic system. Due to this fact, one should assume that the reaction with 5-ethynyl-2,2'-bithiophene proceeds according to the mechanism proposed for other terminal alkynes [39,40,41]. 

### 2.3. Isoxazolines With bt Motifs

**Scheme 5:**
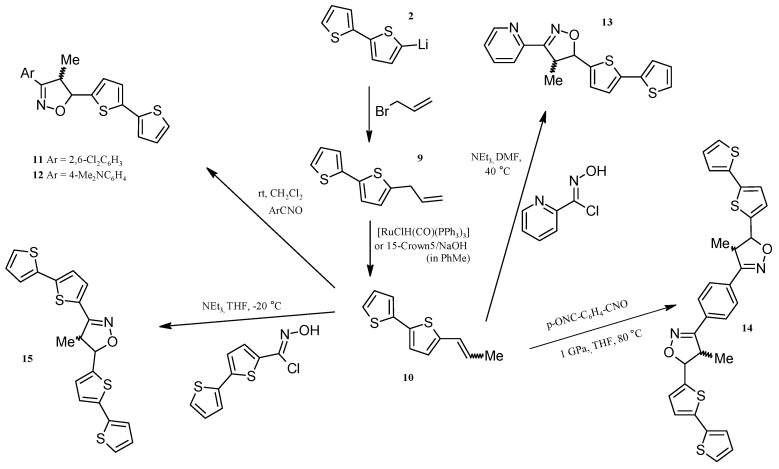
Synthesis of isoxazolines (*E* + *Z*)-(**10**): *cis* + *trans* 3-(2,6-dichlorophenyl-4-methyl-5-(2,2'-bithiophen-5-yl)isoxazoline (**11**); 3-(*p*-dimethylaminophenyl)-5-(2,2'-bithiophen-5-yl)-4-methylisoxazoline (**12**); 3,5-bis(2,2'-bithiophen-5-yl)-4-methylisoxazoline (**15**); 5-(2,2'-bithiophen-5-yl)-4-methyl-3-(2-pyridyl)isoxazoline (**13**); 1,4-bis(5-(2,2'-bithiophen-5-ylo)-4-methylisoxazoline-3-ylo)benzene (**14**).

Five new isoxazolines containing one or two bithienyl moieties were obtained via [Ru]- or 15-crown-5/NaOH catalyzed isomerization of 5-allyl-2,2'-bithiophene **9** to 5-(1-propenyl)-2,2'-bithiophene and then 1,3-DC of nitrile oxide to **10** (Scheme 5). The range of reviews on the synthesis of isoxazolines via the addition of nitrile oxides to alkenes is very extensive and includes many monographic reports [42,43,44,45], and our papers [46,47,48]. The methods of preparation of numerous pure, stable nitrile oxides [49] or generated *in situ* [50,51] have been also described. The synthesis of 2-allyl-thiophene in the reaction of 2-lithiothiophene (obtained by the lithiation of bithiophene with *n*-BuLi in THF at −78 °C) with allyl bromide has been reported [52,53]. However, our attempts to apply this method to the synthesis of **9** were inefficient due to low regioselectivity (lithiation of bithiophene produced more than 35% dilithiobithiophene). Moreover, the synthesis of **9** via reaction of 2-thienyl-magnesium chloride with allyl bromide was described in the literature [54]. However, our attempts to use this method to obtain 5-allyl-2,2'-bithiophene in the reaction of (2,2'-bithiophen-5-yl)magnesium iodide or bromide with allyl bromide were not successful in spite of testing various reaction conditions and various catalysts ([Pd(PPh_3_)_4_], [PdCl_2_(PPh_3_)_2_], [NiCl_2_(dppe)], [NiCl_2_(dppp)]). The application of our procedure of bithiophene lithiation [29] in the hexane-diethyl ether mixture allowed us to obtain high lithiation regioselectivity (less than 0.5% of the dilithio product). Compound **9** was conveniently and effectively obtained in the reaction of **2** with allyl bromide without the catalyst (Scheme 5). Practically quantitative isomerization of 5-allyl-2,2'-bithiophene (**9**) to (*E* + *Z*)-5-(1-propenyl)-2,2'-bithiophene (**10**) (determined by ^1^H-NMR: *E/Z* = 9/1) using [RuClH(CO)(PPh_3_)_3_] was performed. This catalyst is well-known and has been intensively studied by us and used by others [19,55,56]. Additionally, the abovementioned isomerization was realized with a new catalytic system, *i.e*., 15-crown-5/NaOH/PhMe. The latter catalytic system allowed the quantitative transformation of **9** into 1-propenyl derivative **10** even at room temperature (*E/Z* = 10/1). We also verified that this catalytic system is equally efficient in the isomerization of N-allylimidazole to *N*-(1-propenyl)imidazole and allylphenyl sulphide to phenyl-(1-propenyl) sulphide (in each case a mixture of *E* and *Z* isomers was formed). The reactions reached equilibrium Qallyl-Q(1-propenyl), where Q = 1-imidazolyl or PhS at room temperature within 24h in PhMe. Among the catalysts of crown ether-base type, applied for the double bond migration, only 18-crown-6/t-BuOK (for the isomerization of 1-phenyl-2-aza-1,4-pentadiene) [57] and 18-crown-6/KOH/benzene (for the isomerization of N-allylimidazole, PhXCH_2_CH=CH_2_, where X = O, S, Se and several ArCH=NCH_2_CH=CH_2_) have been described so far [46,58].

Compound **10** was used in the synthesis of five new isoxazolines **11**–**15**, containing one or two bithiophene motifs. The compounds **11**–**15** were obtained via 1,3-DC of two stable (2,6-Cl_2_C_6_H_4_CNO and *p*-(ONC)_2_C_6_H_4_), two low stable nitrile oxides (2-pyridinecarbonitrile oxide and *p*-dimethylamino-benzenecarbonitrile oxide) and extremely unstable 2,2'-bithiophen-5-carbonitrile oxide. It is known that sterically hindered nitrile oxides and those containing substituents decreasing the dipole moment of this dipole are stable [59]. The last three oxides (two low stable and one unstable) were generated *in situ* from the proper oxymoyl chlorides using triethylamine (added dropwise) in order to achieve high concentration of the dipolarophile (*E* + *Z*)-5-(1-propenyl)-2,2'-bithiophene. However, the dimerization of these oxides, mainly 2,2'-bithiophen-5-carbonitrile oxide, was observed. Therefore, a 3-fold excess of oxymoyl chloride in relation to the dipolarophile was used and consequently, the quantitative conversion of the latter was attained. When the ratio nitrile oxide/dipolarophile = 1/1, the conversion of **10** was low, e.g., for 2-pyridinecarbonitrile oxide it was 40% and the *E/Z* ratio in the unreacted **10**, *i.e*., after 1,3-DC, was significantly different from that for **10** before 1,3-DC (4/2 and 9/1, respectively). This means that (*E*)-(**10**) underwent cycloaddition much faster than (*Z*)-(**10**). We obtained stable 2-pyridine oxymoyl chloride (described in the literature) and 2,2'-bithiophen-5-carbamoyl chloride from the respective oximes in the reaction with NCS in DMF at room temperature via a typical or modified procedure [60]. Interestingly, in the case of the synthesis of 2,2'-bithiophen-5-carbamoyl chloride via the typical procedure (NCS, HCl in DMF) [60], chlorination of the bithiophene ring takes place. When the procedure was changed, *i.e*., when HCl was no longer used as an initiator, the desired oxymoyl chloride was obtained in high yield (95%) without the chlorination of thiophene ring. Furthermore, the generation of oxides *in situ* in DMF proceeds at room temperature (2-pyridine- and *p*-dimethylaminobenzenecarbonitrile oxides) and in THF at −20 °C (2,2'-bithiophen-5-carbonitrile oxide).

In the case of the cycloaddition reactions activated by high pressure, it is very important to take into consideration the stability and reactivity of ArCNO, and in addition the solubility of ArCNO in organic solvents (Scheme 5). In the case of terephthaloyldinitrile dioxide, which is a low reactivity oxide, performance of the cycloaddition under high pressure gave a good result, *i.e*., with practically quantitative conversion (Scheme 5). It was very important to reach such a conversion since it facilitated the separation of the pure product, which would have been difficult if the product had been a mixture of mono- and diisoxazoline. At room temperature the reaction between *p*-(ONC)_2_C_6_H_4_ and **10** practically did not occur, whereas at 80 °C in a steel reactor (under equilibrium pressure) the yield of isoxazolines was 60%, while 10% was the product of monoaddition. There is no doubt that considerable enhancement of yield is caused by high pressure activation. The reaction shown in Scheme 5 leading to **14** was the first 1,3-DC of nitrile oxide to alkenes under high pressure. In the literature, there are many examples reporting the beneficial, spectacular influence of high pressure on chemical reactions, cycloaddition reactions in particular, due to their highly negative activation volume [60,61,62,63]. There are many papers on diene-ene [4 + 2] cycloadditions but the number of papers on dipolar [3 + 2] cycloaddition is limited, furthermore, most of them concern the cycloaddition of nitrones to alkenes [62], and azides to alkynes [61,63]. There are no reports on the beneficial effects of high pressure on the cycloaddition reaction of nitrile oxide to alkenes. All five 1,3-DC reactions shown in Scheme 5 were concerted, since the *trans/cis* ratio for the isoxazolines obtained was the same as the *E/Z* ratio for the dipolarophile (9/1). On the other hand, the regioselectivity of the cycloaddition presented in Scheme 5 was slightly different. In the case of dipolarophiles of MeCH=CHQ type (as **10**), the regioselectivity was very high or complete when Q was a strong donor, as it has been shown in our previous papers [46,47,48]. The content of the second regioisomer, *i.e*., 3-Ar, 4-bithienyl-5-methylisoxazolines depended on the type of Ar is 5%, 2%, 2% 1% and 1% for 2,6-Cl_2_C_6_H_3_, *p*-Me_2_NC_6_H_4_, bt, C_6_H_4_, 2-Py, respectively. It means that the reactions are highly regioselective, allowing one to obtain pure 5-bithienyl isoxazolines. In the case of the reaction with 2-pyridinecarbonitrile oxide, a side product, *i.e*., oxide dimer was isolated, which structure was confirmed by X-ray spectroscopy (Table S1 in Supporting Information). This dimer is a known compound [64], however, its X-ray structure is not known.

**Scheme 6:**
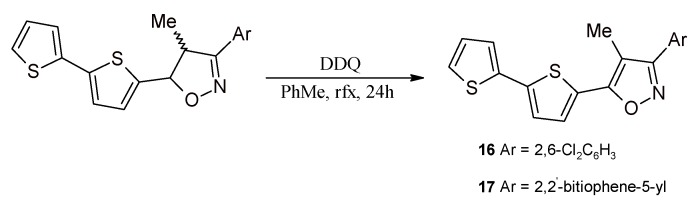
Aromatization of isoxazolines using DDQ to: 3-(2,6-dichlorophenyl)-4-methyl-5-(2,2'-bithiophen-5-yl)isoxazole (**16**) and 3,5-bis(2,2'-bithiophen-5-yl)-4-methylisoxazole (**17**).

The obtained 5-(2,2'-bithiophen-5-yl)-3-(2,6-dichlorophenyl)-4-methylisoxazoline (**11**) and 5-(2,2'-bithiophen-5-yl)-3-(*p*-dimethylaminophenyl)-4-methylisoxazoline (**12**) were assumed as model compounds for 1,3-DC and aromatization. Compounds 1,4-bis(5-(2,2'-bithiophen-5-yl)-4-methylisoxazoline-3-ylo)benzene (**14**) and 3,5-bis(2,2'-bithiophen-5-yl)-4-methylisoxazoline (**15**) are the precursors of conducting polymers of bt-A-bt type, where the spacer A contains an isoxazole ring. However, aromatization before polymerization is necessary. Isoxazoline **12** containing a 2-pyridyl-substituent is particularly interesting—it is a bidendate N,N-donor ligand and a precursor of a ligand which is an analogue of 2,2'-bipyridine (after aromatization). Both the dihydro form (isoxazoline) and, particularly, the fully aromatic form (isoxazole) may be used for the synthesis of molecular materials and conducting polymers. In the literature only a few transition metal (Pd, Pt, Re) complexes with 3-(2-pyridyl)isoxazolines, and also a few Ir and Re complexes with diisoxazoles [65] have been described. Importantly, such conducting polymers from the polythiophene group with isoxazole motif connecting the terthiophene fragments have not been reported. The obtained isoxazolines may be easily transformed into respective isoxazoles via oxidative dehydrogenation by DDQ, as it has been shown for two of them, *i.e*., **16**, **17** (Scheme 6). The aromatization of isoxazolines shown in Scheme 6 was performed using a typical procedure [66].

**Scheme 7:**
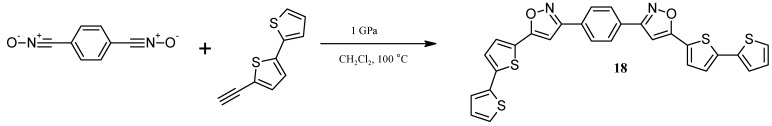
Synthesis of 1,4-bis[5-(2,2'-bithiophen-5-yl)isoxazol-3-yl]benzene (**18**) via high pressure activated 1,3-DC cycloaddition of terephthaloyldinitrile to 5-ethynyl-2,2'-bithiophene.

The alternative route for the synthesis of isoxazoles via addition nitrile oxide to double bond and then aromatization can be the direct addition of RCNO into triple bond—see Scheme 7.

According to us, the reaction shown in Scheme 7 is a particularly spectacular example of the beneficial influence of high pressure on 1,3-DC. The conversion and the selectivity were practically quantitative, whereas under atmospheric or equilibrium pressure, in a steel reactor, this reaction does not take place even at 100 °C (in CH_2_Cl_2_ or DMF).

### 2.4. Rh-Mediated [2 + 2 + 2] Cycloaddition for Synthesis of Fluoranthene and Benzene Derivatives with a bt Motif

**Scheme 8:**
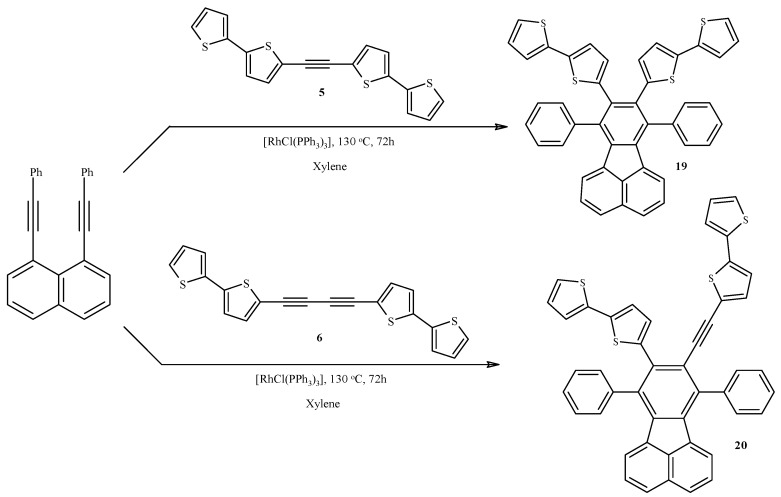
Rh-mediated synthesis of fluoranthene derivatives containing 2,2'-bithiophen-5-yl moiety: di(2,2'-bithienyl-5-yl)diphenylfluoranthene (**19**) and (2,2'-bithiophen-5-yl)[(2,2'-bithiophen-5-yl)ethynyl]diphenylfluoranthene (**20**).

Two new 7,8,9,10-tetrasubstituted fluoranthenes **19**, **20** were obtained from 1,8-diethynylnaphthalene and 1,2-bis(2,2'-bithiophen-5-yl)ethyne (**5**) or 1,4-bis(2,2'-bithiophen-5-yl)-1,3-butadiyne (**6**) derivatives via [RhCl(PPh_3_)_3_]-mediated formal [2 + 2 + 2] cycloaddition (Scheme 8). 

[RhCl(PPh_3_)_3_] was applied as a catalyst since, together with [Co_2_(CO)_8_], it is the most frequently applied and effective catalyst for both inter- and intramolecular [2 + 2 + 2] cycloadditions [67,68,69]. In the literature there are only a few papers devoted to the synthesis of 7,8,9,10-tetrasubstituted fluoranthenes from derivatives of 1,8-diethynylnaphthalene and alkynes, except for conjugated diynes, or NBD which can act as an acetylene equivalent catalyzed by various rhodium complexes [67,68,69]. To date, fluoranthenes with alkyl or phenyl substituents, except for the ones with heteroaryl substituent, have been obtained in this way. Recently, several fluoranthenes with substituted thiophene motifs (except for the ones with a bithiophene one) and two fluoranthenes with a 6*H*-indolo[2,3-b]quinazoline chromophore [70] have been obtained. The compounds were obtained via [4 + 2], but not via [2 + 2 + 2] cycloaddition. The novel compounds **19** and **20** are the first bithiophene derivatives of the bt-A-bt type, where the fluoroanthene fragment plays a role of a spacer. The polymers obtained from them are expected to have interesting conducting and luminescence properties.

**Scheme 9:**
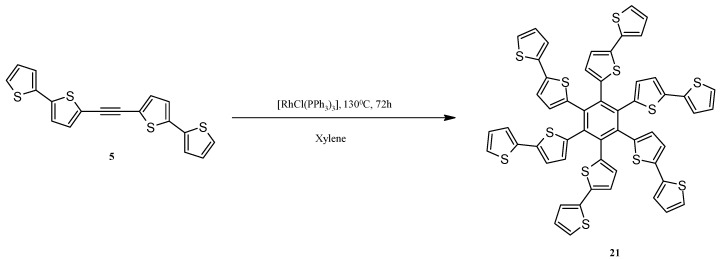
Cyclotrimerization of 1,2-bis(2,2'-bithiophen-5-yl)acetylene (**5**) to hexa(2,2'-bithiophen-5-yl)benzene (**21**) catalyzed by [RhCl(PPh_3_)_3_].

The methods known from the literature were applied for the synthesis of diiodonaphthalene [71], was obtained from commercially available 1,8-diaminonaphthalene, which was further used to obtain a phenyl derivative via Sonogashira coupling [72]. The trimerization of the acetylene derivative **5**, both in the reaction shown in Scheme 9 and during heating of **5** in xylene at 130 °C in the presence of 10 mol% [RhCl(PPh_3_)_3_] was observed. The latter reaction allowed to obtain pure hexa(2,2'-bithiophen-5-yl)benzene (**21**) with 40% yield (Scheme 9). In the literature, there is only one work devoted to the trimerization of ArC≡CAr (Ar is alkyl-substituted- thienyl, bithienyl or terthienyl) to hexakis(alkylthienyl-, bithienyl and terthienyl)benzenes catalyzed by [Co_2_(CO)_8_] [73].

Our result, *i.e*., the trimerization of **5**, corresponds to the results of other authors, who used [RhCl(PPh_3_)_3_] with very good results for fully intermolecular [2 + 2 + 2] cycloaddition [67]. However, trimerization of 1,4-bis(2,2'-bithiophen-5-yl)-1,3-butadiyne was not observed despite the fact that such a reaction is known for 1,4-diphenyl-1,3-butadiyne [67]. It probably results from the steric effects being greater for the 2,2-bitihophen-5-yl substituent than for the phenyl one, which have a major influence on the course of the studied cycloaddition reactions. One should remember that this reaction probably proceeds via a metalacyclopentadiene intermediate and steric hindrance plays a crucial role in this transformation [67]. Moreover, in accordance with the results of Wu *et al*., the thermal, intramolecular transformation of 1,8-di(phenylethyl)naphthalene to 7-phenylbenzo[k]fluoranthene described by Bossenbroek *et al*., was not observed by us [74].

### 2.5. Catalytically or High Pressure Activated 1,3-DC of Azides to Triple Bond for Synthesis of Triazoles with bt Moiety

**Scheme 10:**
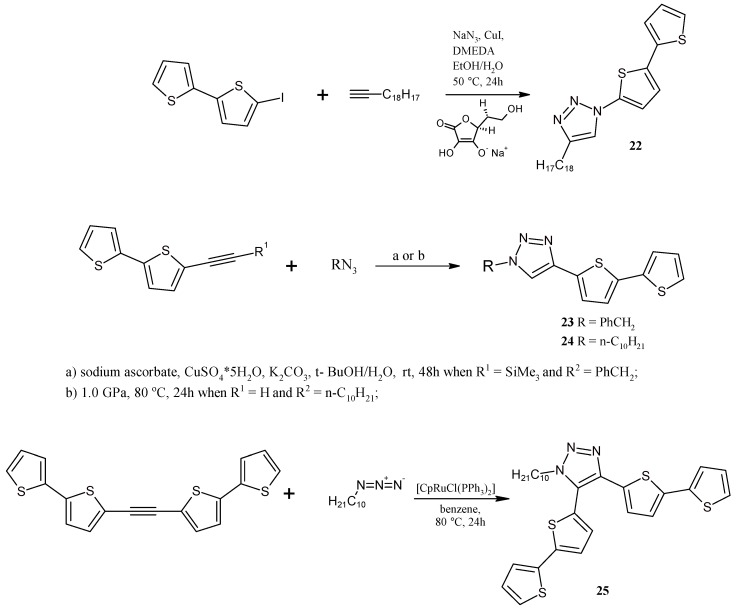
Cu- or Ru-catalyst or hp-mediated synthesis of triazoles **22**–**25** with a bt moiety.

Using 5-iodobithiophene (**3**), 5-ethynylbithiophene (**4**), 1,2-bis(2,2'-bithiophen-5-yl)acetylene (**5**) and Cu-catalyst or Ru-catalyst or high pressure mediated 1,3-dipolar cycloaddition of azides to alkynes, four novel triazole derivatives **22**–**25** containing one or two bt moieties were obtained (Scheme 10). In these syntheses, typical catalytic systems, *i.e*., CuSO_4_/sodium ascorbate [75,76,77] or [RuClcp(PPh_3_)_2_] [78,79] or high pressure activation were used. The use of high pressure in the 1,3-DC of azides to alkynes has already been described [63,80], although not for heteroaryl-substituted alkynes. Decyl, benzyl, and bithienyl azides [13] were synthesized using the methods known from the literature (the latter was generated *in situ*).

It should be noted that the reaction carried out under high pressure is fully regioselective—only 4-(2,2'-bithiophen-5-yl)triazole is formed. The obtained triazoles may play the role as cyclometalating ligands or monomers for the synthesis of polythiophenes, where the triazole ring is the linker connecting tetrathiophene fragments. Up till now, this type of conducting polythiophenes has not been known. Moreover, there are a lot of triazoles which play a vital role of ligands in the molecular catalysis described in the literature [75]. Additionally, a triazole motif is present in the structure of many pharmaceuticals, in particular antifungal ones [81].

### 2.6. Synthesis of Pyrroles with two bt Moieties via CuCl-Mediated Dihydroamination of 1,4-Bis(2,2'-bithiophene-5-yl)buta-1,3-diyne

**Scheme 11:**
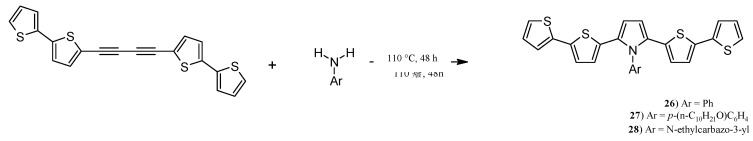
Synthesis of: *N*-phenyl-2,5-bis(2,2'-bithiophen-5-yl)pyrrole (**26**); *N*-(p-decyloxyphenyl)-2,5-bis(2,2'-bithiophen-5-yl)pyrrole (**27**) and *N*-[2-(N-carbazo-3-yl)ethyl]-2,5-bis(2,2'-bithiophen-5-yl)pyrrole (**28**) via CuCl-catalyzed dihydroamination of 1,4-bis(2,2'-bithiophen-5-yl)-1,3-butadiyne (**6**).

Three new pyrrole derivatives **26**–**28** were also obtained via CuCl-mediated hydroamination of 1,4-bis(2,2'-bithiophen-5-yl)buta-1,3-diyne (**6**) (Scheme 11). The addition of RNH_2_ to 1,3-diynes (hydroamination of 1,3-diynes) catalyzed with transition metal complexes leading to pyrrole derivatives is an alternative to classical Knorr, Paal-Knorr, and Hantzsch methods. The effective catalysts of these reactions are complexes of Au(I) [82,83], Au(I)-H_3_PO_4_-12WO_3_ [84], and CuCl [85,86,87]—the yields of these reactions range from moderate to very high. Convenient procedures for many disubstituted diynes, even for 1,4-bis(2-thienyl)-1,3-butadiyne are known [85,86,87,88]. Moreover, several aromatic and aliphatic amines were used for the abovementioned procedures [85,86,87,88].

It was shown that it is possible to obtain pyrroles with 2,2'-bithiophen-5-yl substituents in positions 2 and 5, and most of all, that aminoheteroarenes may also serve as substrates in this synthesis. Importantly, the synthesis yield of the derivative substituted with *N*-ethylcarbazo-3-yl was much higher than for phenyl and *p*-decyloxyphenyl substituents. The synthesized compounds **26**–**28** will be certainly very interesting as nanomaterials due to the π-excessive character of the pyrrole and bithiophene fragments, luminescence, and solubility in organic solvents. There are only two similar systems known in the literature [89]. Nevertheless, these systems contain a thiophene (not bithiophene) motif, *i.e*., 3,6-bis(2,5-di-2-thienyl-1*H*-pyrrol-1-yl)-9-ethyl(or dodecyl)-9*H*-carbazole, which were classically obtained from 1,4-bis(2-thienyl)buta-1,4-dione [89]. The mechanism of dihydroamination of conjugated diynes catalysed by CuCl has not been described in detail so far. Nevertheless, it is probably similar to the well-known addition of amines to disubstituted acetylenes catalyzed by various transition metal complexes [90].

## 3. Experimental Section 

### 3.1. General Methods

All starting materials and reagents were purchased from commercial sources and were used as received, unless otherwise stated. All reactions were carried out under argon atmospheres undernhydrous conditions. Solvents were dried and purified using usual methods before use. Thin layer chromatography was performed on silica gel (TLC silica gel 60, Merck, Darmstadt, Germany). NMR spectra were recorded on an Avance 400 instrument (400 MHz for ^1^H and 100 MHz for ^13^C) or Ascend 500 instrument (500 MHz for ^1^H and 125 MHz for ^13^C) (Bruker, Billerica, MA, USA). Chemical shifts were referenced to the residual proton signal of the solvent. Melting point measurements were conducted on Stuart automatic melting point SMP40 apparatus (Bibby Scientific Limited Group, Staffordshire, United Kingdom). Low resolution mass spectra were recorded in methanol on a Varian LC-920 instrument (Varian, Palo Alto, CA, USA). HRMS-ESI spectra were recorded in methanol on a Synapt G2-S HDMS mass spectrometer (Waters Inc., Milford, MA, USA) equipped with an electrospray ion source and q-TOF type mass analyzer. HRMS-EI spectra were recorded on an AutoSpec Premier magnetic sector mass spectrometer (Waters Inc.) equipped with an electron impact (EI) ion source and the EBE double focusing geometry mass analyzer. The setup for reactions under high pressure conditions was built for high pressure dielectric measurements by UNIPRESS (Warsaw, Poland) and described by Paluch *et al*. [91].

The crystals of compounds were mounted in turn on a Gemini Ultra Oxford Diffraction automatic diffractometer (Agilent, Santa Clara, CA, USA) equipped with a CCD detector, and used for data collection. X-ray intensity data was collected with graphite monochromated Mo*K_α_* radiation (λ = 0.71073 Å) at a temperature of 295.0(2) K, with ω scan mode. Ewald sphere reflections were collected up to 2θ 50.10. Details concerning crystal data and refinement is gathered in Tables S1–S3. Lorentz, polarization and empirical absorption corrections using spherical harmonics implemented in SCALE3 ABSPACK scaling algorithm [92]. The structures were solved by the direct method and subsequently completed by difference Fourier recycling. All the non-hydrogen atoms were refined anisotropically using full-matrix, least-squares techniques. The Olex2 [93] and SHELXS97, SHELXL97 [94] programs were used for all the calculations. Atomic scattering factors were incorporated in the computer programs.

### 3.2. Synthesis of 1,2-Bis(2,2'-bithiophene-5-yl)acetylene (**5**) and 1,4-Bis(2,2'-bithiophene-5-yl)buta-1,3-diyne (**6**)

A cascade of three reactors, each containing a mixture of substrates consisting of 5-iodo-2,2'-bithiophene (15.0 g, 51.3 mmol), CuI (1.125 g, 5.9 mmol), [PdCl_2_(PPh_3_)_2_] (0.75 g, 1.1 mmol), acetone (500 mL), triethylamine (11.25 mL, 81 mmol), was saturated with a steady stream of acetylene (for **5**) or buta-1,3-diyne (generated from 1,4-dichloro-2-butyne (12.64 g, 0.1 mol), according to the procedure described in literature [95] in temperature range from 70 to 75 °C for 30 min, 1 mol/6 h for **6**), mixed with argon (1:10 v/v) at room temperature for 6 h. After the flow of gas was finished, the content of the reactors was left to stir for 24 h at room temperature. Then the volatile fractions from the combined mixtures were evaporated on a rotary evaporator. Crude product was purified using column chromatography (SiO_2_, hexane). A yellow solid was obtained in 60% and 75% yield for **5** and **6**, respectively.

Compound **5**:


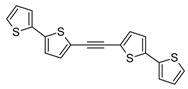


^1^H-NMR (CDCl_3_): δ 7.25 (dd, *J* = 5.1 Hz, J = 3.6 Hz, 2H), 7.20 (dd, *J* = 3.6 Hz, *J* = 1.1 Hz, 2H), 7.18 (d, *J* = 3.8 Hz, 2H), 7.07 (d, *J* = 3.8 Hz, 2H), 7.03 (dd, *J* = 5.1 Hz, *J* = 3.6 Hz, 2H). ^13^C-NMR (CDCl_3_): δ 139.53, 136.79, 133.15, 128.12, 125.17, 124.50, 123.77, 121.55, 87.43. HRMS (EI) calcd. for C_18_H_10_S_2_ 353.96654 found 353.93537.

Compound **6**:


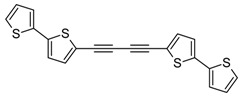


^1^H-NMR (CDCl_3_): δ 7.26 (dd, *J* = 5.1 Hz, *J* = 1.1 Hz, 2H), 7.25 (d, *J* = 3.8 Hz, 2H), 7.21 (dd, *J* = 3.6 Hz, *J* = 1.1 Hz, 2H), 7.05 (d, *J* = 3.8 Hz, 2H), 7.03 (dd, *J* = 5.1 Hz, *J* = 3.6 Hz, 2H). ^13^C-NMR (100 MHz, CDCl_3_): δ 140.82, 136.56, 135.52, 128.19, 125.69, 124.92, 123.74, 120.66, 79.16, 77.59. HRMS (EI) calcd for C_20_H_10_S_4_ [M]^+^ 377.9665 found 377.9669.

### 3.3. Synthesis of 1,4-Bis(2,2'-bithiophene-5-yl)-3-methyl-2-ethoxycarbonylbenzene (**7**) and 1,4-Bis(2,2'-bithiophene-5-yl)-3-phenyl-2-ethoxycarbonylbenzene (**8**)

The mixture of ethyl acetoacetate (for synthesis of **7**) or ethylbenzoyl acetate (for synthesis of **8**) (1.28 mmol) with 5-ethynyl-2,2'-bithiophene (609 mg, 3.20 mmol), [MnBr(CO)_5_] (20 mg, 0.0735 mmol), and powdered 4 Å molecular sieves (23 mg; 115 wt %-Mn cat.) was vigorously stirred and heated at 80 °C for 48 h. Crude product was purified using column chromatography (SiO_2_, toluene-petroleum ether 3:1). Compounds **7** and **8** were isolated in 60% and 70% yield, respectively, as yellow solids.

*1,4-Bis(2,2'-bithiophene-5-yl)-3-methyl-2-etoxycarbonylbenzene* (**7**). ^1^H-NMR (400 MHz, CDCl_3_): δ 7.48 (d, *J =* 8.0 Hz, 1H), 7.38 (d, *J =* 8.0 Hz, 1H), 7.25–7.23 (m, 2H), 7.21 (dd, *J =* 3.4 Hz, *J =* 1.0 Hz, 1H), 7.20 (dd, *J =* 3.4 Hz, *J =*1.0 Hz, 1H), 7.17 (d, *J =* 3.7 Hz, 1H), 7.13 (d, *J =* 3.7 Hz, 1H), 7.06–7.02 (m, 3H), 6.97 (d, *J =* 3.7 Hz, 1H), 4.30 (q, *J =* 7.1 Hz, 2H), 2.45 (s, 1H), 1.24 (t, *J =* 7.1 Hz, 3H). ^13^C-NMR (125 MHz, CDCl_3_): δ 169.68, 140.63, 140.12, 138.22, 137.94, 137.10, 137.08, 134.74, 134.27, 133.44, 131.44, 131.18, 128.03, 127.90, 127.37, 127.07, 124.62, 124.59, 124.15, 123.88, 123.84, 61.62, 18.05, 13.96. HRMS (EI) calcd for C_26_H_20_O_2_S_4_ [M]^+^ 492.0346 found 492.0361.

*1,4-Bis(2,2'-bithiophene-5-yl)-3-phenyl-2-etoxycarbonylbenzene* (**8**). ^1^H-NMR (400 MHz, CDCl_3_): δ 7.67 (d, *J =* 8.2 Hz, 1H), 7.55 (d, *J =* 8.2 Hz, 1H), 7.36–7.27 (m, 5H), 7.23 (dd, *J =* 5.0 Hz, *J =*0.7 Hz, 1H), 7.20 (dd, *J =* 3.6 Hz, *J =*0.8 Hz, 1H), 7.17 (dd, *J =* 5.1 Hz, *J =*0.9 Hz, 1H), 7.12 (d, *J =* 3.7 Hz, 1H), 7.09 (d, *J =* 3.8 Hz, 1H), 7.05 (dd, *J =* 3.5 Hz, *J =*0.8 Hz, 1H), 7.03 (dd, *J =* 5.0 Hz, *J =*3.6 Hz, 1H), 6.97 (dd, *J =* 5.0 Hz, *J =*3.6 Hz, 1H), 6.89 (d, *J =* 3.8 Hz, 1H), 6.50 (d, *J =* 3.8 Hz, 1H), 3.90 (q, *J =* 7.1 Hz, 2H), 0.89 (t, *J =* 7.1 Hz, 3H). ^13^C-NMR (125 MHz, CDCl_3_): δ 168.55, 140.72, 139.72, 138.31, 138.02, 138.00, 137.87, 137.16, 137.11, 134.85, 133.25, 130.49, 130.30, 130.26, 129.14, 128.09, 128.00, 127.89, 127.79, 127.31, 124.64, 124.41, 124.30, 123.90, 123.69, 123.61, 61.23, 13.56. HRMS (EI) calcd for C_31_H_22_O_2_S_4_ [M]^+^ 554.0503 found 554.0482.

### 3.4. Synthesis of 5-Allyl-2,2'-bithiophene (**9**)

1.6 M solution of *n*-BuLi in hexane (60.2 mL, 96.2 mmol) was injected through septum with a syringe (for about 30 min.) to vigorously stirred solution of 2,2'-bithiophene (**1**, 20 g, 120.3 mmol) in the mixture of hexane (1 L) and diethyl ether (350 mL) cooled down to below −5 °C. It was necessary to keep the temperature of reaction mixture below −5 °C during the injection of *n*-BuLi solution. After all n-BuLi was added, the cooling bath was removed and reaction mixture was left to reach room temperature. In the next step, allyl bromide (14.5 g, 120.3 mmol) was added dropwise through septum with a syringe. The reaction mixture was stirred for 72 h at room temperature and the progress of the reaction was monitored with TLC. After that, the post-reaction mixture was filtered and the solvents were evaporated from the filtrate using a rotary evaporator. The crude product was purified using column chromatography (SiO_2_, hexane). Product **9** was obtained as yellowish liquid in 85% yield.

#### Isomerization of 5-Allyl-2,2'-bithiophene (**9**), N-allylimidazole and Allyl-phenyl sulfide to 1-propenyl derivatives catalysed by 15-crown-5/NaOH 

Allyl substrate (10 mmol), 15-crown-5 (10 mmol), micronized NaOH (10 mmol) and toluene (1 mL) were vigorously stirred at room temperature for 24 h. After that, toluene (10 mL) was added and the mixture was washed with water (3 times 10 mL) and dried over anhydrous MgSO_4_. After drying an agent was filtered out, activated carbon (20 mg) was added and the mixture was stirred for 3 h at room temperature. After that, activated carbon was filtered out and the solvent was evaporated on a rotary evaporator. Pure *(E + Z)*-(1-propenyl) derivatives were obtained with 97% yield.

### 3.5. Synthesis of (E) + (Z)-5-(1-Propenyl)-2,2'-bithiophene (**10**)

A mixture of 5-allyl-2,2'-bithiophene (**9**) (2 g, 10 mmol), [RuClH(CO)(PPh_3_)_3_] (10 mg, 0.1 mmol) and toluene (1 mL) was stirred at 60 °C for 24 h. The solvent was evaporated using a rotary evaporator and the solid residue was purified using column chromatography (SiO_2_, hexane). Ru-free **10** was obtained with 97% yield (E/Z = 9) as a light-brown oil.

Compound **(10)**:


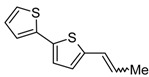


^1^H-NMR (400 MHz, C_6_D_6_): δ 1.54 (dd, *J =* 6.7 Hz, *J =* 1.7 Hz, 3H), 1.81 (dd, *J =* 7.3 Hz, *J =* 1.7 Hz, 3H), 5.47 (dq, *J =* 11.4 Hz, *J =* 7.3 Hz, 1H), 5.89 (dq, *J =* 15.5 Hz, *J =* 6.7 Hz, 1H), 6.23 (dtd, *J =* 15.5 Hz, *J =* 1.6 Hz, *J =* 1,1 Hz, 1H), 6.39 (ddd, *J =* 11.4 Hz, *J =* 1.7 Hz, *J =* 0.5 Hz, 1H), 6.50 (d, *J =* 3,7 Hz, 1H), 6.52 (d, *J =* 3,7 Hz, 1H), 6.64 (dd, *J =* 5.1 Hz, *J =* 3.6 Hz, 1H), 6.65 (dd, *J =* 5.1 Hz, *J =* 3.6 Hz, 1H), 6.71 (dd, *J =* 5.1 Hz, *J =* 1.1 Hz, 2H), 6.88 (d, *J =* 3.7 Hz, 1H), 6.93 (d, *J =* 3.7 Hz, 1H), 6.08 (dd, *J =* 3.6 Hz, *J =* 1.2 Hz, 1H), 7.00 (dd, *J =* 3.6 Hz, *J =* 1.2 Hz, 1H). ^13^C-NMR (100 MHz, CDCl_3_): δ 142.26, 137.84, 137.53, 134.93, 127.93, 127.92, 127.92, 126.10, 126.10, 125.08, 125.08, 124.47, 124.46, 124.46, 124.20, 123.92, 123.92, 123.88, 123.88, 123.50, 18.52, 15.39. HRMS (EI) calcd for C_11_H_10_S_2_ [M]^+^ 206.02239 found 206.02162.

### 3.6. General Procedure for 1,3-Dipolar Cycloaddition: Synthesis of **11**–**13**

To a solution of aldoxime (1.29 mmol) and NCS (1.50 mmol) in CH_2_Cl_2_ (or DMF for **13**) (12 mL), one drop of 36% hydrochloric acid was added. The reaction mixture was stirred at room temperature for 24 h. After that time, (*E* + *Z*)-5-(1-propenyl)-2,2'-bithiophene (**10**) (266 mg, 1.29 mmol) and triethylamine (0.10 mL, 1.41 mmol) were added and the reaction mixture was stirred at 40 °C for 24 h. In the next step, solvent was removed using a rotary evaporator. The crude product was purified using column chromatography (SiO_2_, CH_2_Cl_2_). Products **11** (61% yield, yellow solid), **12** (29% yield, pale-red oil), and **13** (34% yield, pale-brown oil) were isolated. During isolation of the isoxazoline **13** the dimer of 2-pyridinecarbonitrile oxide **13a** was additionally obtained (5% yield, pale yellow crystals).

*5-(2,2'-Bithiophen-5-yl)-3-(2,6-dichlorophenyl)-4-methylisoxazoline* (**11**). ^1^H-NMR (400 MHz, CDCl_3_): δ 7.44–7.31 (m, 3H), 7.26 (d, *J =* 5.1 Hz, 1H), 7.21 (d, *J =* 3.6 Hz, 1H), 7.10 (s, 2H), 7.05 (dd, *J =* 4.6 Hz, *J =* 3.6 Hz, 1H), 6.08 (d, *J =* 10.3 Hz, 1H), 5.50 (d, *J =* 10.0 Hz, 1H), 4.98 (dq, *J =* 9.3 Hz, *J =* 6.1 Hz, 1H), 4.87 (d, *J =* 9.4 Hz, 1H), 4.09–4.01 (m, 1H), 3.97 (dq, *J =* 10.0 Hz, *J =* 7.1 Hz, 1H), 1.65 (d, *J =* 6.1 Hz, 1H), 1.31 (d, *J =* 7.1 Hz, 3H), 1.00 (d, *J =* 7.5 Hz, 1H). ^13^C-NMR (100 MHz, CDCl_3_): δ 160.88, 139.80, 136.38, 135.98, 131.32, 128.17, 128.10, 127.95, 127.72, 127.38, 125.46, 124.63, 124.23, 109.70, 77.35, 77.04, 76.72, 8.09. m.p. 149 ± 1.2°C. HRMS (EI) calcd for C_18_H_13_Cl_2_NOS_2_ [M]^+^: 392.98156 found 392.98123.

*4-[5-(2,2'-Bithiophen-5-yl)-4-methyl-4,5-dihydroisoxazol-3-yl]-N,N-dimethylaniline* (**12**). cis^1^H-NMR (400 MHz, CDCl_3_): δ 1.43 (d, *J =* 7.1 Hz, 3H), 2.88 (s, 6H), 3.77 (qd, *J =* 7.1 Hz, *J =* 4.8 Hz, 1H), 5.45 (dd, *J =* 4.8 Hz, *J =* 0.5 Hz , 1H), 6.69 (dd, *J =* 8.4 Hz, *J =* 2.1 Hz, 1H), 6.96 (dd, *J =* 3.7 Hz, *J =* 0.6 Hz, 1H), 6.99 (dd, *J =* 5.1 Hz, *J =* 3.6 Hz, 1H), 7.02 (d, *J =* 3.6 Hz, 1H), 7.12 (dd, *J =* 3.6 Hz, *J =* 1.1 Hz, 1H), 7.20 (dd, *J =* 5.1 Hz, *J =* 1.1 Hz, 1H), 7.55 (dd, *J =* 8.4 Hz, *J =* 2.1 Hz, 1H), 7.72 (d, *J =* 2.1 Hz, 1H). trans^1^H-NMR (400 MHz, CDCl_3_): δ 1.44 (d, *J =* 7.1 Hz, 3H), 3.00 (s, 6H), 3.78 (dq, *J =* 7.1 Hz, *J =* 4.4 Hz, 1H), 5.41 (dd, *J =* 4.4 Hz, *J =* 0.5 Hz , 1H), 6.70–6.73 (m, 2H), 6.96 (dd, *J =* 3.7 Hz, *J =* 0.6 Hz, 1H), 6.98 (dd, *J =* 5.1 Hz, *J =* 3.6 Hz, 1 H), 7.01 (d, *J =* 3.6 Hz, 1H),7.11 (dd, *J =* 3.6 Hz, *J =* 1.1 Hz, 1H), 7.18 (dd, *J =* 5.1 Hz, *J =* 1.1 Hz, 1H), 7.58–7.62 (m, 2H). cis^13^C-NMR (100 MHz, CDCl_3_): δ 17.76, 43.45, 50.51, 85.76, 115.78, 122.88, 123.33, 124.02, 124.73, 125.82, 126.34, 129.51, 137.06, 137.74, 142.21, 151.93, 159.60. trans^13^C-NMR (100 MHz, CDCl_3_): δ 17.99, 40.24, 50.89, 85.10, 111.95, 119.86, 123.28, 123.89, 124.57, 125.54, 127.87, 128.46, 137.24, 137.43, 143.05, 151.54, 160.80. MS (ESI) *m/z* 369.11 [M+H]^+^, HRMS (ESI): calcd for C_20_H_21_N_2_OS_2_ [M+H]^+^ 369.1095 found 369.1096.

*2-[5-(2,2'-Bithiophen-5-yl)-4-methyl-4,5-dihydroisoxazol-3-yl]pyridine* (**13**). ^1^H-NMR (400 MHz, CDCl_3_): δ 1.53 (d, *J =* 7.1 Hz, 3H), 4.09 (dq, *J =* 7.1 Hz, *J =* 5.7 Hz, 1H), 5.50 (dd, *J =* 5.6 Hz, *J =* 0.5 Hz , 1H), 6.98 (dd, *J =* 5.1 Hz, *J =* 3.6 Hz, 1H), 6.99 (dd, *J =* 3.7 Hz, *J =* 0.7 Hz, 1H), 7.02 (d, *J =* 3.7 Hz, 1H),7.12 (dd, *J =* 3.6 Hz, *J =* 1.1 Hz, 1H), 7.18 (dd, *J =* 5.1 Hz, *J =* 1.1 Hz, 1H), 7.29 (ddd, *J =* 7.5 Hz, *J =* 4.9 Hz, *J =* 1.2 Hz, 1H), 7.72 (td, *J =* 7.8 Hz, *J =* 1.8 Hz, 1H), 8.04 (dt, *J =* 8.0 Hz, *J =* 1.0 Hz, 1H), 8.61 (ddd, *J =* 4.9 Hz, *J =* 1.7 Hz, *J =* 0.9 Hz, 1H). ^13^C-NMR (125 MHz, CDCl_3_): δ 17.63, 50.30, 86.34, 122.51, 123.39, 124.00, 124.35, 124.71, 125.94, 127.88, 136.57, 137.10, 137.84, 142.02, 148.87, 149.35, 161.82. HRMS (EI): calcd for C_17_H_14_N_2_OS_2_ [M+H]^+^ 326.0548 found 326.0555.

*2-Pyridinecarbonitrile Oxide Dimer* (**13a**). ^1^H-NMR (400 MHZ, CDCl_3_) δ = 7.32–7.35 ppm (m, 1H, C_Ar-H_), 7.38–7.41 (m, 1H, C_Ar-H_), 7.82–7.86 (m, 3H, C_Ar-H_), 7.97 (d, *J =* 8.0 Hz, 1H, C_Ar-H_), 8.53 (d, *J =* 4.6 Hz, 1H, C_Ar-H_), 8.56 (d, *J =* 4.8 Hz, 1H, C_Ar-H_). ^13^C-NMR (100 MHz, CDCl_3_) δ = 114.6 ppm, 123.8, 124.3, 124.6, 125.0, 136.7, 136.8, 143.7, 146.8, 149.6, 149.7, 156.3 (C_Ar_). MS (ESI^+^) *m/z* 240.0 [M]^+^, HRMS (ESI^+^): calcd for C_12_H_8_N_4_O_2_ [M]^+^ 240.0647 found 240.0642. IR (KBr): 3051, 2925, 2853, 1598, 1567, 1487, 1403, 1341, 1285, 1135, 1090, 993, 965, 827, 792, 749.

### 3.7. Synthesis of 1,4-Bis(5-(2,2'-bithiophen-5-ylo)-4-methylisoxazoline-3-yl)benzene (**14**) under High Pressure

A solution of (*E* + *Z*) 5-(1-propenyl)-2,2'-bithiophene (**10**) (536 mg, 2.60 mmol) and terephthalo-bis(nitrile *N*-oxide) (192 mg, 1.2 mmol) in CH_2_Cl_2_ (2 mL) was placed in a Teflon capsule and heated at 100 °C for 4h under pressure of 1 GPa. After that, volatile fractions were removed using a rotary evaporator. Crude product was purified using column chromatography (SiO_2_, CH_2_Cl_2_/hexane 1:3). Product **14** was obtained as an orange solid with 99% yield. ^1^H-NMR (400 MHz, CDCl_3_): δ 7.77 (s, 4H), 7.21 (d, *J =* 5.1 Hz, 2H), 7.13 (d, *J =* 3.4 Hz, 2H), 7.05–6.96 (m, 6H), 5.50 (d, *J =* 5.0 Hz, 2H), 3.84 (dq, *J =* 6.9, 5.4 Hz, 2H), 1.46 (d, *J =* 7.1 Hz, 6H). ^13^C-NMR (100 MHz, CDCl_3_): δ 160.14, 160.13, 141.77, 137.87, 136.94, 130.14, 127.88, 127.57, 125.99, 124.77, 124.04, 123.30, 86.08, 50.23, 17.62. HRMS (EI) calcd for C_30_H_24_S_4_N_2_O_2_ [M]+ 572.0721 found 572.0737.

### 3.8. Synthesis of 3,5-Bis(2,2'-bithiophen-5-yl)-4-methylisoxazoline (**15**)

To a stirred, cooled down to −25 °C solution of 2,2'-bithiophene-5-carbox-imidoyl chloride (2.37 g, 9.72 mmol) and (*E* + *Z*) 5-(1-propenyl)-2,2'-bithiophene (**10**) (1.88 g, 9.19 mmol) in tetrahydrofuran (250 mL), triethylamine (1.65 mL, 13.67 mmol) was added dropwise. After all amine was added, the reaction mixture was left to reach room temperature and the solvent was evaporated on a rotary evaporator. Crude product was purified using column chromatography (SiO_2_, CHCl_3_). Product **15** was isolated with 60% yield as a yellow solid. ^1^H-NMR (400 MHz, CDCl_3_): δ 7.30 (dd, *J =* 5.2 Hz, *J =* 1.1 Hz, 1H), 7.27 (dd, *J =* 3.6 Hz, *J =* 1.1 Hz, 1H), 7.23 (dd, *J =* 5.2 Hz, *J =* 1.0 Hz, 1H), 7.21 (d, *J =* 3.8 Hz, 1H), 7.16 (dd, *J =* 3.7 Hz, *J =* 1.5 Hz, 2H), 7.15 (d, *J =* 3.7 Hz, 2H), 7.07 (dd, *J =* 5.1 Hz, *J =* 3.7 Hz, 1H), 7.05 (d, *J =* 3.8 Hz, 1H), 7.02 (dd, *J =* 5.1 Hz, *J =* 3.7 Hz, 1H), 7.00 (d, *J =* 3.8 Hz, 1H), 5.48 (d, *J =* 5.3 Hz, 1H), 3.78 (qd, *J =* 7.1 Hz, *J =* 5.3 Hz, 1H), 1.55 (d, *J =* 7.1 Hz, 3H). ^13^C-NMR (125 MHz, CDCl_3_): δ 156.51, 141.79, 140.07, 138.55, 137.89, 135.71, 133.48, 129.00, 128.72, 127.82, 126.01, 125.72, 123.92, 123.51, 123.45, 123.26, 123.18, 85.84, 51.44, 15.98. HRMS (ESI) calcd for C_20_H_15_NOS_4_Na ([M+Na^+^]: 435,99287 found 435,99415.

### 3.9. Synthesis of 5-(2,2'-Bithiophen-5-yl)-3-(2,6-dichlorophenyl)-4-methylisoxazole (**16**) and 3,5-Bis(2,2'-bithiophene-5-yl)-4-methylisoxazole (**17**)

DDQ (575 mg, 2.54 mmol) was added to a solution of isoxazoline (**11**) (for product **16**) or (**15**) (for product **17**) (1.27 mmol) in toluene (140 mL), and the reaction mixture was heated under reflux for 24 h and vigorously stirred. After that time, the product was still observed in the reaction mixture with TLC, thus two more loads of DDQ (2 × 575 mg, 2 × 2.54 mmol) were added at an interval of 24 h (the progress of the reaction was monitored with TLC). After 72 h, the conversion was complete (TLC). Post-reaction mixture was concentrated to a small volume and loaded on a chromatography column (SiO_2_, toluene). Products **16** and **17** were obtained with 95 and 90% yields respectively, **16** as an orange-red solid, **17** as an orange solid.

*5-(2,2'-Bithiophene-5-yl)-3-(2,6-dichlorophenyl)-4-methylisoxazole* (**16**). ^1^H-NMR (400 MHz, CDCl_3_): δ 7.49–7.35 (m, 3H), 7.29 (dd, *J =* 5.1 Hz, *J =* 1.2 Hz, 1H), 7.28 (dd, *J =* 3.7 Hz, *J =* 1.2 Hz, 1H), 7.24 (d, *J =* 3.9 Hz, 1H), 7.19 (dd, *J =* 5.1 Hz, *J =* 1.2 Hz, 1H), 7.09 (dd, *J =* 3.6 Hz, *J =* 1.2 Hz, 1H), 7.06 (dd, *J =* 5.1 Hz, *J =* 3.7 Hz, 1H), 7.00 (d, *J =* 3.7 Hz, 1H), 6.98 (dd, *J =* 5.1 Hz, *J =* 3.6 Hz, 1H), 2.69 (s, 1H), 2.09 (s, 3H). ^13^C-NMR (100 MHz, CDCl_3_): δ 160.88, 139.80, 136.38, 135.98, 131.32, 128.17, 128.10, 127.95, 127.72, 127.38, 125.46, 124.63, 124.23, 109.70, 77.35, 77.04, 76.72, 8.09. HRMS (EI) calcd for C_18_H_11_S_2_NOCl_2_ [M]^+^ 390.9659 found 390.9663. m.p. 134.8 ± 0.5 °C.

*3,5-Bis(2,2'-bithiophene-5-yl)-4-methylisoxazole* (**17**). ^1^H-NMR (400 MHz, CDCl_3_): δ 7.47 (d, *J =* 3.9 Hz, 1H), 7.44 (d, *J =* 3.8 Hz, 1H), 7.34–7.29 (m, 2H), 7.26 (d, *J =* 3.9 Hz, 1H), 7.25 (d, *J =* 3.8 Hz, 1H), 7.10–7.09 (m, 1H), 7.08 (dd, *J =* 5.3 Hz, *J =* 3.5 Hz, 1H), 2.48 (s, 3H). ^13^C-NMR (100 MHz, CDCl_3_): δ 157.95, 139.89, 139.52, 136.60, 136.33, 128.90, 128.29, 128.09, 128.02, 127.74, 127.57, 126.33, 125.49, 125.24, 124.66, 124.60, 124.16, 124.02, 107.81, 77.33, 77.01, 76.69, 9.27. HRMS (EI) calcd for C_20_H_13_NOS_4_ [M]^+^ 410.9880 found 410.9891.

### 3.10. Synthesis of1,4-Bis[5-(2,2'-bithiophen-5-yl)isoxazol-3-yl]benzene (**18**) under high pressure

A solution of 5-ethynyl-2,2'-bithiophene (494 mg, 2.60 mmol) (**4**) and terephthalo-bis(nitrile *N*-oxide) (192 mg, 1.2 mmol) in CH_2_Cl_2_ (2 mL) was placed in a Teflon capsule and heated at 100 °C for 4 h under pressure of 1 GPa. After that, volatile fractions were removed using a rotary evaporator. Crude product was purified using column chromatography (SiO_2_, CH_2_Cl_2_/hexane 1:3). Product **18** was obtained as an orange solid with 99% yield.

### 3.11. Synthesis of 8,9-Bis(2,2'-bithiophen-5-yl)-7,10-(diphenyl)fluoranthene (**19**) and 9-(2,2'-Bithiophene-5-yl)-8-(5-ethynyl-2,2'-bithiophen-5-yl)-7,10-(diphenyl)fluoranthene (**20**)

1,8-Bis(phenylethynyl)naphthalene (11 mg, 0.03 mmol) and 5,5'-ethyne-1,2-diylbis(2,2'-bithiophene) (**5**) (59 mg, 0.17 mmol) for the synthesis of **19** or 5,5'-buta-1,3-diyne-1,4-diylbis(2,2'-bithiophene) (**6**) (61 mg, 0.16 mmol) for the synthesis of **20**, and Wilkinson’s catalyst (3 mg, 0.003 mmol) were placed in a screw capped glass vial. Xylene (3 mL) was added, and the vial was tightly capped and placed in thermostated oil bath in 130 °C and stirred for 72 h. After that, the vial was cooled down and xylene was evaporated on a rotary evaporator. Crude solid residue was purified on chromatography column (SiO_2_) using petroleum ether (for **19**) or hexane:dichloromethane 3:1 (for **20**) as an eluent. Products **19** and **20** were obtained with 60 and 70% yield, respectively. **19** as a yellow solid, **20** as a yellowish oil.

*8,9-Bis(2,2'-bithiophene-5-yl)-7,10-(diphenyl)fluoranthene* (**19**). ^1^H-NMR (400 MHz, CDCl_3_): δ 7.74 (d, *J =* 8.2 Hz, 2H), 7.36–7.44 (m, 10H), 7.29–7.33 (m, 2H), 7.09 (dd, *J =* 5.0 Hz, *J =* 1.1 Hz, 2H), 6.95 (dd, *J =* 3.6 Hz, *J =* 1.1 Hz, 2H), 6.90 (dd, *J =* 4.9 Hz, *J =* 3.6 Hz, 2H), 6.74 (d, *J =* 3.7 Hz, 2H), 6.59 (d, *J =* 7.1 Hz, 2H), 6.51 (d, *J =* 3.7 Hz, 2H). ^13^C-NMR (100 MHz, CDCl_3_): δ 139.84, 139.49, 138.65, 137.98, 137.86, 137.71, 136.13, 133.89, 133.44, 130.25, 129.90, 129.76, 128.57, 127.87, 127.70, 127.62, 127.11, 123.84, 123.81, 123.21, 122.79. HRMS (EI) calcd for C_44_H_26_S_4_ [M]^+^ 682.0917 found 682.0923.

*9-(2,2'-Bithiophene-5-yl)-8-(5-ethynyl-2,2'-bithiophene-5-yl)-7,10-(diphenyl)fluoranthene* (**20**). ^1^H-NMR (400 MHz, CDCl_3_): δ 7.75 (t, *J =* 7.7 Hz, 2H), 7.57–7.67 (m, 5H), 7.40–7.48 (m, 5H), 7.34–7.37 (m, 1H), 7.29–7.33 (m, 1H), 7.18 (dt, *J =* 5.0 Hz, *J =* 1.0 Hz, 2H), 7.12 (dd, *J =* 3.6 Hz, *J =* 1.1 Hz, 1H), 7.02 (dd, *J =* 3.6 Hz, *J =* 1.2 Hz, 1H), 6.98–7.01 (m, 2H), 6.96 (dd, *J =* 5.0 Hz, *J =* 3.6 Hz, 1H), 6.87–6.89 (m, 2H), 6.82 (d, *J =* 3.7 Hz, 1H), 6.59–6.61 (m, 2H). ^13^C-NMR (100 MHz, CDCl_3_): δ 139.72, 139.34, 139.16, 139.01, 138.27, 138.06, 137.94, 137.57, 137.18, 137.03, 136.16, 135.67, 134.90, 133.51, 132.38, 130.12, 129.95, 129.82, 129.77, 128.80, 128.73, 128.19, 127.97, 127.88, 127.26, 127.21, 124.90, 124.16, 124.12, 123.85, 123.83, 123.62, 123.54, 123.52, 123.03, 122.34, 94.44, 91.96. HRMS (ESI) calcd for C_46_H_27_S_4_ [M+H]^+^ 707.0996 found 707.1010.

### 3.12. Synthesis of Hexa(2,2'-bithiophen-5-yl)benzene (**21**)

5,5'-Ethyne-1,2-diylbis(2,2'-bithiophene) (**5**, 50 mg, 0.14 mmol) and Wilkinson’s catalyst (13 mg, 0.014 mmol) were placed in a screw capped glass vial. Xylene (3 mL) was added, and the vial was tightly capped and placed in a thermostated oil bath in 130 °C and was stirred for 72 h. During that time, the precipitation of grey solid in the reaction mixture was observed. After heating, the vial was cooled down, and the precipitate was filtered. Crude solid residue was washed with xylene and diethyl ether, and dried overnight at room temperature. Product 21 was isolated with 40% yield as a grey solid. 1.1 Hz, 6H), 6.99 (d, *J =* 3.7 Hz, 6H), 6.97 (dd, *J =* 5.1 Hz, *J =* 3.6 Hz, 6H), 6.77 (d, *J =* 3.7 Hz, 6H). ^13^C-NMR (125 MHz, DMSO-d_6_): δ 138.01, 137.78, 136.53, 135.99, 131.06, 128.25, 125.32, 123.80, 122.98. HRMS (EI) calcd for C_54_H_30_S_12_ [M]^+^ 1061.8996 found 1061.8977.

### 3.13. Synthesis of 1-(2,2'-Bithiophen-5-yl)-4-octyl-1,2,3-triazole (**22**)

A mixture of 5-iodo-2,2'-bithiophene (**3**, 0.29 g, 1 mmol), ethanol-water solution (4 mL, 7:3 v/v), sodium azide (130 mg, 2 mmol), CuI (19 mg, 0.1 mmol), sodium ascorbate (20 mg, 0.1 mmol), 1-decyne (0.18 mL, 1 mmol) and *N,N'*-dimethylethylenediamine (20 μL, 0.2 mmol) was vigorously stirred at 50 °C for 24h. After that time, the post-reaction mixture was cooled down to room temperature and 25% solution of ammonia (10 mL) and ethyl acetate (15 mL) were added. Organic layer was washed with water (3 times 15 mL) and dried over anhydrous MgSO_4_. Volatile fractions were removed on a rotary evaporator and a solid residue was purified on chromatography column (SiO_2_, CH_2_Cl_2_). Product **22** was obtained as a dark solid with 70% yield. ^1^H-NMR (400 MHz, CDCl_3_): δ 7.63 (s, 1H), 7.22 (dd, *J =* 5.2 Hz, *J =* 1.1 Hz, 1H), 7.20 (dd, *J =* 3.6 Hz, *J =* 1.1 Hz, 1H), 7.09 (d, *J =* 4.0 Hz, 1H), 7.06 (d, *J =* 4.0 Hz, 1H), 7.05 (dd, *J =* 5.1 Hz, *J =* 3.6 Hz, 1H), 2.78 (t, *J =* 7.7 Hz, 2H), 1.72 (dt, *J =* 15.3 Hz, *J =* 7.8 Hz, 2H), 1.32 (m, 10H), 0.88 (t, *J =* 6.8 Hz, 3H). ^13^C-NMR (100 MHz, CDCl_3_): δ 149.36, 136.96, 136.19, 134.59, 128.12, 126.37, 124.53, 122.32, 119.79, 117.80, 31.96, 29.44, 29.42, 29.36, 29.32, 25.72, 22.77, 14.21. HRMS (ESI) calcd for C_18_H_24_N_3_S_2_ [M+H]^+^ 346.1412 found 346.1412.

### 3.14. Synthesis of 1-Benzyl-4-(2,2'-bithiophen-5-yl)-1,2,3-triazole (**23**)

A mixture of 5-(trimethylsilyl)ethynyl-2,2'-bithiophene (150 mg, 0.571 mmol), benzylazide (110 mg, 0.857 mmol), K_2_CO_3_ (87 mg, 0.063 mmol), CuSO_4_·5H_2_O (110 mg, 0.046 mmol), sodium ascorbate (270 mg, 1.37 mmol), pyridine (0.6 mL, 7.46 mmol) and *tert*-butanol-water mixture (14 mL, 1:1 v/v) was stirred at room temperature for 48h. After that time, 25% solution of ammonia (10 mL) and ethyl acetate (15 mL) was added. Organic layer was washed with water (3 × 15 mL) and dried over anhydrous MgSO_4_. Volatile fractions were removed on a rotary evaporator and a solid residue was purified on chromatography column (SiO_2_, CH_2_Cl_2_/CH_2_Cl_2_ + 5% MeOH). Product **23** was obtained as a dark solid with 50% yield. ^1^H-NMR (400 MHz, CDCl_3_): δ 7.56 (s, 1H), 7.43–7.37 (m, 3H), 7.33–7.30 (m, 2H), 7.23 (d, *J =* 8.8 Hz, 1H), 7.22 (dd, *J =* 5.1 Hz, *J =* 1.1 Hz, 1H), 7.18 (dd, *J =* 3.6 Hz, *J =* 1.1 Hz, 1H), 7.11 (d, *J =* 3.8 Hz, 1H), 7.02 (dd, *J =* 5.1 Hz, *J =* 3.6 Hz, 1H). ^13^C-NMR (100 MHZ, CDCl_3_) δ 143.12, 137.22, 137.09, 134.53, 131.69, 129.33, 129.01, 128.24, 128.02, 124.84, 124.68, 124.21, 123.98 119.04, 53.43. HRMS (ESI) calcd for C_17_H_14_N_3_S_2_ [M+H]^+^ 324.0629 found 324.0629.

### 3.15. Synthesis of 1-Decyl-4-(2,2'-Bithiophen-5-yl)-1,2,3-triazole (**24**) under High Pressure

A solution of 5-ethynyl-2,2'-bithiophene (**4**) (100 mg, 0.525 mmol) and decylazide (144 mg, 0.788 mmol) in CH_2_Cl_2_ (2 mL) was placed in a teflon capsule and heated at 80 °C for 24h under pressure of 1 GPa. After that, volatile fractions were removed using a rotary evaporator. Crude product was purified using column chromatography (SiO_2_, CH_2_Cl_2_). Product **24** was obtained as a dark solid with 60% yield. ^1^H-NMR (400 MHz, CDCl_3_): δ 7.65 (s, 1H), 7.26 (d, *J =* 3.8 Hz, 1H), 7.22 (dd, *J =* 5.1 Hz, *J =* 1.1 Hz, 1H), 7.20 (dd, *J =* 3.6 Hz, *J =* 1.1 Hz, 1H), 7.13 (d, *J =* 3.8 Hz, 1H), 7.02 (dd, *J =* 5.1 Hz, *J =* 3.6 Hz, 1H), 4.36 (t, *J =* 7.2 Hz, 2H), 1.93 (m, 2H), 1.29 (m, 14H), 0.87 (t, *J =* 6.8 Hz, 3H). ^13^C-NMR (100 MHz, CDCl_3_): δ 142.57, 137.27, 136.93, 131.96, 127.99, 124.66, 124.62, 124.21, 123.92, 118.99, 50.63, 31.95, 30.39, 29.56, 29.48, 29.35, 29.10, 26.58, 22.76, 14.20. HRMS (ESI) calcd for C_20_H_28_N_3_S_2_ [M+H]^+^ 374.1725 found 374.1727.

### 3.16. Synthesis of 1-Decyl-4,5-bis(2,2'-bithiophen-5-yl)-1,2,3-triazole (**25**)

The mixture of decylazide (61 mg, 0.33 mmol), bbta(**5**) (150 mg, 0.42 mmol), benzene (7 mL) and [RuCl(cp)(PPh_3_)_2_] (8 mg, 0.011 mmol) was stirred at 80 °C for 24 h. After that, volatile fractions were removed using a rotary evaporator. Crude product was purified using column chromatography (SiO_2_, hexane:ethyl acetate 9:1). Product **25** was obtained as a dark solid with 60% yield. ^1^H-NMR (400 MHz, CDCl_3_): δ 7.31 (dd, *J =* 5.1 Hz, *J =* 1.1 Hz, 1H), 7.29 (d, *J =* 3.7 Hz, 1H), 7.27 (dd, *J =* 3.6 Hz, *J =* 1.1 Hz, 1H), 7.20 (dd, *J =* 5.1 Hz, *J =* 1.1 Hz, 1H), 7.16 (dd, *J =* 3.6 Hz, *J =* 1.1 Hz, 1H), 7.13–7.11 (m, 2H), 7.07 (dd, *J =* 5.1 Hz, *J =* 3.6 Hz, 1H), 7.04 (d, *J =* 3.8 Hz, 1H), 6.99 (dd, *J =* 5.1 Hz, *J =* 3.6 Hz, 1H), 4.28 (m, 2H), 1.87 (m, 2H), 1.26 (m, 14H), 0.86 (t, *J =* 6.8 Hz, 3H). ^13^C-NMR (100 MHz, CDCl_3_): δ 141.99, 141.71, 137.49, 137.27, 136.24, 131.94, 131.63, 128.23, 127.98, 125.69, 125.66, 125.56, 124.91, 124.66, 124.33, 124.26, 124.15, 124.08, 48.88, 31.99, 30.44, 29.60, 29.48, 29.40, 29.07, 26.59, 22.79, 14.25. HRMS(EI) calcd for C_28_H_31_N_3_S_4_ [M]^+^ 537.14009 found: 537.13955.

### 3.17. Synthesis of N-phenyl-2,5-bis(2,2'-bithiophen-5-yl)pyrrole (**26**), N-(p-decyloxy)phenyl-2,5-bis(2,2'-bithiophen-5-yl)pyrrole (**27**) and N-(2-carbazolylethyl)-2,5-bis(2,2'-bithiophen-5-yl)pyrrole (**28**)

A mixture of 1,4-bis(2,2'-bithiophen-5-yl)buta-1,3-diyne (**6**, 50 mg, 0.132 mmol), CuCl (1.31 mg, 0.0132 mmol) and aniline (0.12 mL, 1.32 mmol) (for **26**) or *p*-(decyloxyphenyl)aniline (330 mg, 2.64 mmol) (for **27**) or *N*-ethyl-3-aminocarbazole (560 mg, 2.64 mmol) (for **28**) was stirred at 110 °C for 48 h. After that time, post-reaction mixture was cooled down to room temperature and CH_2_Cl_2_ (50 mL) was added. Mixture was filtered and the filtrate was washed with 0.5 M solution of NaCN in water (3 × 20 mL). The organic layer was dried over anhydrous MgSO_4_ and volatile fractions were removed using a rotary evaporator. Crude residue was dissolved in hexane:CH_2_Cl_2_ mixture (5:1) and loaded on chromatography column (SiO_2_). Compounds **26**, **27** and **28** were isolated as yellow solids with 30, 31 and 74% yield, respectively. For the isolation of pure products, the following eluents were used: hexane:CH_2_Cl_2_ (5:1) for **26** and **27**; hexane:CHCl_3_ (5:2) with 1% of triethylamine for **28**.

*N-phenyl-2,5-bis(2,2'-bithiophene-5-yl)pyrrole* (**26**). ^1^H-NMR (400 MHz, CDCl_3_): δ 7.51 (m, 3H), 7.40 (dd, *J =* 8.1 Hz, *J =* 1.4 Hz, 2H), 7.17 (dd, *J =* 5.1 Hz, *J =* 1.1 Hz, 2H), 7.06 (dd, *J =* 3.6 Hz, *J =* 1.1 Hz, 2H), 6.98 (dd, *J =* 5.1 Hz, *J =* 3.6 Hz, 2H), 6.89 (d, *J =* 3.8 Hz, 2H), 6.60 (s, 2H), 6.36 (d, *J =* 3.8 Hz, 2H). ^13^C-NMR (100 MHz, CDCl_3_): δ 138.44, 137.42, 135.67, 133.88, 130.25, 130.10, 129.63, 129.56, 127.89, 124.72, 124.23, 123.90, 123.45, 110.24. HRMS (EI) calcd for C_26_H_17_NS_4_ [M]^+^ 471.0244 found 471.02434.

*N-(p-decyloxy)phenyl-2,5-bis(2,2'-bithiophene-5-yl)pyrrole* (**27**). ^1^H-NMR (400 MHz, CDCl_3_): δ 7.27 (m, 2H), 7.15 (dd, *J =* 5.1 Hz, *J =* 1.1 Hz, 2H), 7.04 (dd, *J =* 3.6 Hz, *J =* 1.1 Hz, 2H), 6.97 (m, 4H), 6.88 (d, *J =* 3.8 Hz, 2H), 6.56 (s, 2H), 6.40 (d, *J =* 3.8 Hz, 2H), 4.03 (t, *J =* 6.6 Hz, 2H), 1.84 (m, 2H), 1.33 (s, 15H), 0.88 (t, *J =* 6.6 Hz, 3H). ^13^C-NMR (100 MHz, CDCl_3_): δ 160.04, 137.53, 135.50, 134.11, 131.07, 130.76, 130.55, 127.88, 124.48, 124.17, 123.96, 123.42, 115.32, 109.88, 68.57, 32.06, 29.74, 29.72, 29.59, 29.48, 29.37, 26.21, 22.83, 14.26. HRMS (EI) calcd for C_36_H_37_NOS_4_ [M]^+^ 627.1758 found 627.1757.

*N-(2-carbazolylethyl)-2,5-bis(2,2'-bithiophen-5-yl)pyrrole* (**28**). ^1^H-NMR (400 MHz, CDCl_3_): δ 8.12 (d, *J =* 1.8 Hz, 1H), 8.05 (d, *J =* 7.8 Hz, 1H), 7.50 (m, 5H), 7.09 (dd, *J =* 5.1 Hz, *J =* 1.1 Hz, 2H), 6.94 (dd, *J =* 3.6 Hz, *J =* 1.1 Hz, 2H), 6.90 (dd, *J =* 5.0 Hz, *J =* 3.6 Hz, 2H), 6.77 (d, *J =* 3.9 Hz, 2H), 6.63 (s, 2H), 6.30 (d, *J =* 3.9 Hz, 2H), 4.46 (q, *J =* 7.2 Hz, 2H), 1.53 (t, *J =* 7.2 Hz, 3H). ^13^C-NMR (100 MHz, CDCl_3_): δ 140.88, 140.17, 137.49, 135.15, 134.47, 130.98, 129.56, 127.80, 127.22, 126.57, 124.22, 124.04, 124.00, 123.57, 123.33, 122.83, 122.16, 121.16, 119.52, 109.74, 109.11, 109.05, 38.07, 14.05. HRMS (EI) calcd for C_34_H_24_N_2_S_4_ [M]^+^ 588.0822 found 588.0837.

## 4. Conclusions

This work reports several highly effective new catalytically or high-pressure-activated reactions and routes leading to diverse 2,2**'**-bithiophene derivatives. New Pd/Cu-mediated coupling reactions, Mn-, Rh-, Cu- or Ru-catalyst mediated various type of cycloaddition, Ru-complex or 15-crown-5/NaOH promoted double bond migration in allylic systems and Cu/NEt_3_-mediated complexation of metal centre were presented. Thanks to these new catalytic reactions and catalytic routes (combined with non-catalytic processes), we obtained novel acetylene, butadiyne, isoxazole, 1,2,3-triazole, pyrrole, benzene and fluoranthene derivatives bearing one, two or six 2,2'-bithiophen-5-yl (bt) moieties. The obtained compounds are of general formulas: bt-A, bt-A-bt and (bt)_6_A, where 2,2'-bithiophen-5-ylic fragments are connected by -C≡C-, -C≡C-C≡C-, aryl and heteroaryl. The “first generation” substrate containing bithiophene motif was 5-lithio-2,2'-bithiophene, which was converted into 5-iodo- and 5-allyl-2,2'-bithiophenes (“the second generation” substrate). In the next step, the above mentioned compounds were transformed catalytically to 5-ethynyl-2,2'-bithiophene, 1,2-bis(2,2'-bithiophene-5-yl)acetylene, 5-(1-propenyl)-2,2'-bithiophene, and 1,4-bis(2,2'-bithiophene-5-yl)buta-1,3-diyne (“the third generation” substrates). Moreover, *in situ* generated 2,2'-bithiophen-5-carbonitrile oxide and 2,2'-bithiophen-5-yl azide belonging to the third-generation substrates were used. For the synthesis of 1,2-bis(2,2'-bithiophene-5-yl)acetylene and 1,4-bis(2,2'-bithiophene-5-yl)buta-1,3-diyne gaseous acetylene and butadiyne and very effective cascade reactor systems were used. All the obtained compounds including bithiophene motif can be applied in organic electronics as monomers for new conducting polythiophenes or as luminescence materials. Moreover, some of them, for instance triazole derivatives, can play the role of cyclometalated ligands, while others should interest scientists searching for new medical probes and pharmaceutics, *i.e*., antifungal ones. Our preliminary examination, presented in the supporting information, has shown that some of the obtained compounds have very interesting luminescence properties and the derivatives which contain at least two bt motifs can be easily electropolymerized into conducting polymers.

## Supplementary Materials

Supplementary materials can be accessed at: http://www.mdpi.com/1420-3049/20/03/4565/s1.
