# Supplementary material for: Multifaceted Strategy for the Synthesis of Diverse 2,2'-Bithiophene Derivatives
Source: Molecules. 2015 Mar 12;20(3):4565–93. doi: 10.3390/molecules20034565 (PMC6272589; doi:10.3390/molecules20034565)
Supplement: Supplementary file 1 [file molecules-20-04565-s001.pdf]

Supplementary Materials

1. X-ray of 2-Pyridinecarbonitrile Oxide Dimer, 1,4-bis(2,2'-Bithiophene-5-yl)buta-1,3-Diyne and 5,5'-Diiodo-2,2'-Bithiophene

1.1. X-ray of 2-Pyridinecarbonitrile Oxide Dimer

Table S1. Crystal data and structure refinement details of 2-pyridinecarbonitrile oxide dimer.

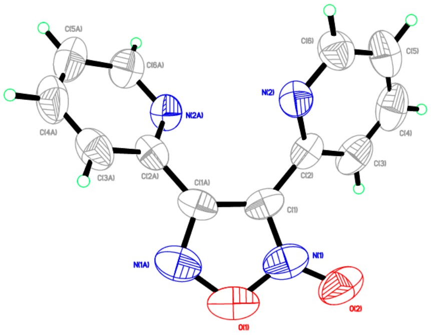

2-pyridinecarbonitrile Oxide Dimer	
Empirical formula	$C_{12}H_8N_4O_2$
Formula weight	240.22
Temperature [K]	295.0(2) K
Crystal system	orthorhombic
Space group	*F*dd2
Unit cell dimensions	
a [Å]	21.6726(17)
b [Å]	12.9433(11)
c [Å]	8.0819(8)
	90
	90
	90
Volume [Å^3]	2267.1(3)
Z	8
Calculated density [Mg/m^3]	1.408
Absorption coefficient [mm^{-1}]	0.101
F(000)	992
Crystal dimensions [mm]	0.35 × 0.11 × 0.06
θ range for data collection [°]	3.67 to 25.05
Index ranges	$-24 \leq h \leq 25$ $-11 \leq k \leq 15$ $-9 \leq l \leq 7$
Reflections collected	3495
Independent reflections	943 [$R_{(int)}$ = 0.0298]
Data/restraints/parameters	942/1/87
Flack parameter	0.03(5)
Goodness-of-fit on F^2	0.945
Final R indices [I > 2σ(I)]	R_1 = 0.0313 wR_2 = 0.0654
R indices (all data)	R_1 = 0.0399 wR_2 = 0.0679
Largest diff. Peak and hole	0.082 and −0.110
CCDC number	973,854

1.2. X-ray of 1,4-bis(2,2'-Bithiophene-5-yl)buta-1,3-Diyne

Table S2. Crystal data and structure refinement details of 1,4-bis(2,2'-bithiophen-5-yl)-1,3-butadiyne (**6**).

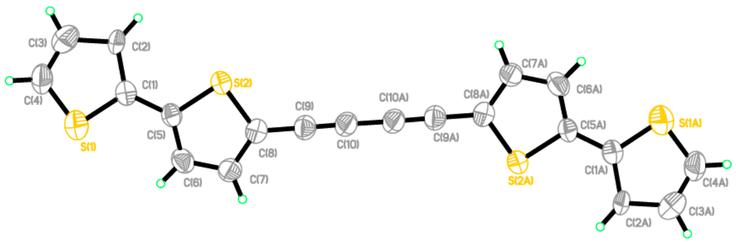

**1,4-bis(2,2'-bithiophen-5-yl)-1,3-butadiyne**	
Empirical formula	$C_{20}H_{10}S_4$
Formula weight	378.52
Temperature [K]	295.0(2) K
Crystal system	monoclinic
Space group	$P2_1/n$
Unit cell dimensions	
a [Å]	5.6618(5)
b [Å]	15.1071(17)
c [Å]	10.3902(10)
	90
	99.823(8)
	90
Volume [Å^3]	875.68(15)
Z	2
Calculated density [Mg/m^3]	1.436
Absorption coefficient [mm^{-1}]	0.540
F(000)	388
Crystal dimensions [mm]	0.38 × 0.19 × 0.11
θ range for data collection [°]	3.35 to 25.05
	$-6 \leq h \leq 6$
Index ranges	$-17 \leq k \leq 17$
	$-10 \leq l \leq 12$
Reflections collected	4174
Independent reflections	1551 [$R_{(int)}$ = 0.0229]
Data/restraints/parameters	1551/0/109
Goodness-of-fit on F^2	1.052
Final R indices [I > 2σ(I)]	R_1 = 0.0480
	wR_2 = 0.0835
R indices (all data)	R_1 = 0.0612
	wR_2 = 0.1145
Largest diff. Peak and hole	0.835 and −0.570
CCDC number	922476

1.3. X-ray of 5,5'-diiodo-2,2'-Bithiophene

Table S3. Crystal data and structure refinement details of 5,5'-diiodo-2,2'-bithiophene.

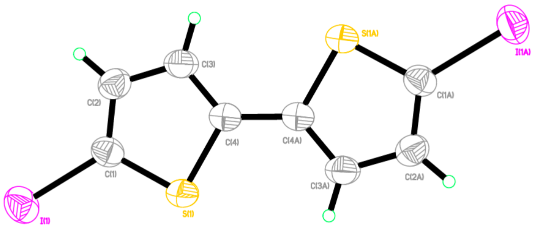

5,5'-diiodo-2,2'-bithiophene	
Empirical formula	$C_8H_4I_2S_2$
Formula weight	418.03
Temperature [K]	295.0(2) K
Crystal system	orthorhombic
Space group	*P*ccn
Unit cell dimensions	
a [Å]	23.4532(15)
b [Å]	7.6546(6)
c [Å]	5.9622(4)
	90
	90
	90
Volume [Å^3]	1070.36(13)
Z	4
Calculated density [Mg/m^3]	2.594
Absorption coefficient [mm^{-1}]	6.210
F(000)	760
Crystal dimensions [mm]	0.22 × 0.11 × 0.04
θ range for data collection [°]	3.47 to 25.05
Index ranges	$-27 \leq h \leq 27$
	$-8 \leq k \leq 9$
	$-5 \leq l \leq 7$
Reflections collected	2820
Independent reflections	943 [$R_{(int)}$ = 0.0258]
Data/restraints/parameters	943/0/55
Goodness-of-fit on F^2	1.012
Final R indices [I > 2σ(I)]	R_1 = 0.0280
	wR_2 = 0.0648
Goodness-of-fit on F^2	1.012
R indices (all data)	R_1 = 0.0343
	wR_2 = 0.0671
Largest diff. Peak and hole	0.430 and −0.631
CCDC number	922,580

2. ^{1}H and ^{13}C-NMR Spectra

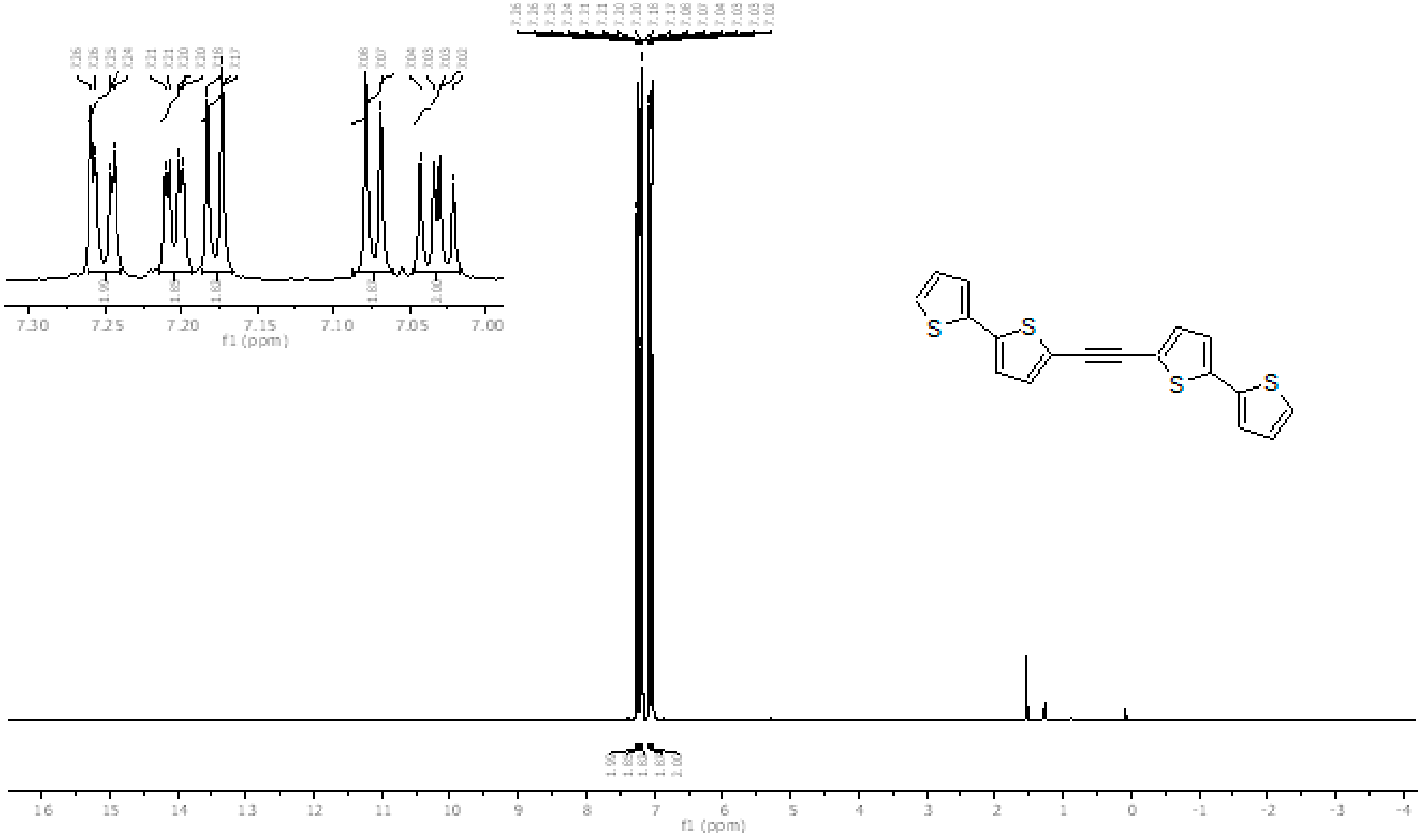

Figure S1. ^{1}H-NMR spectrum of compound (**5**).

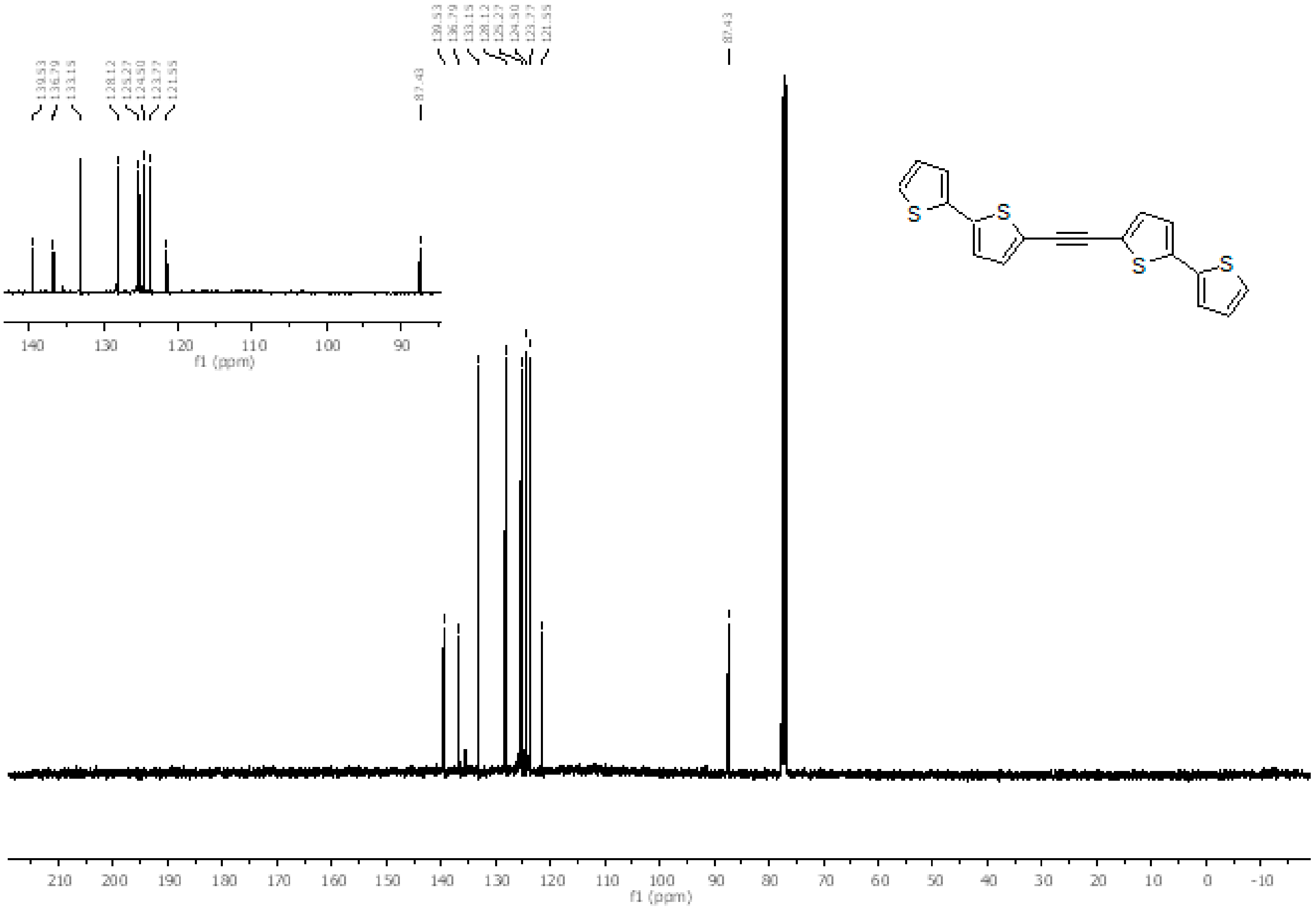

Figure S2. ^{13}C-NMR spectrum of compound (**5**).

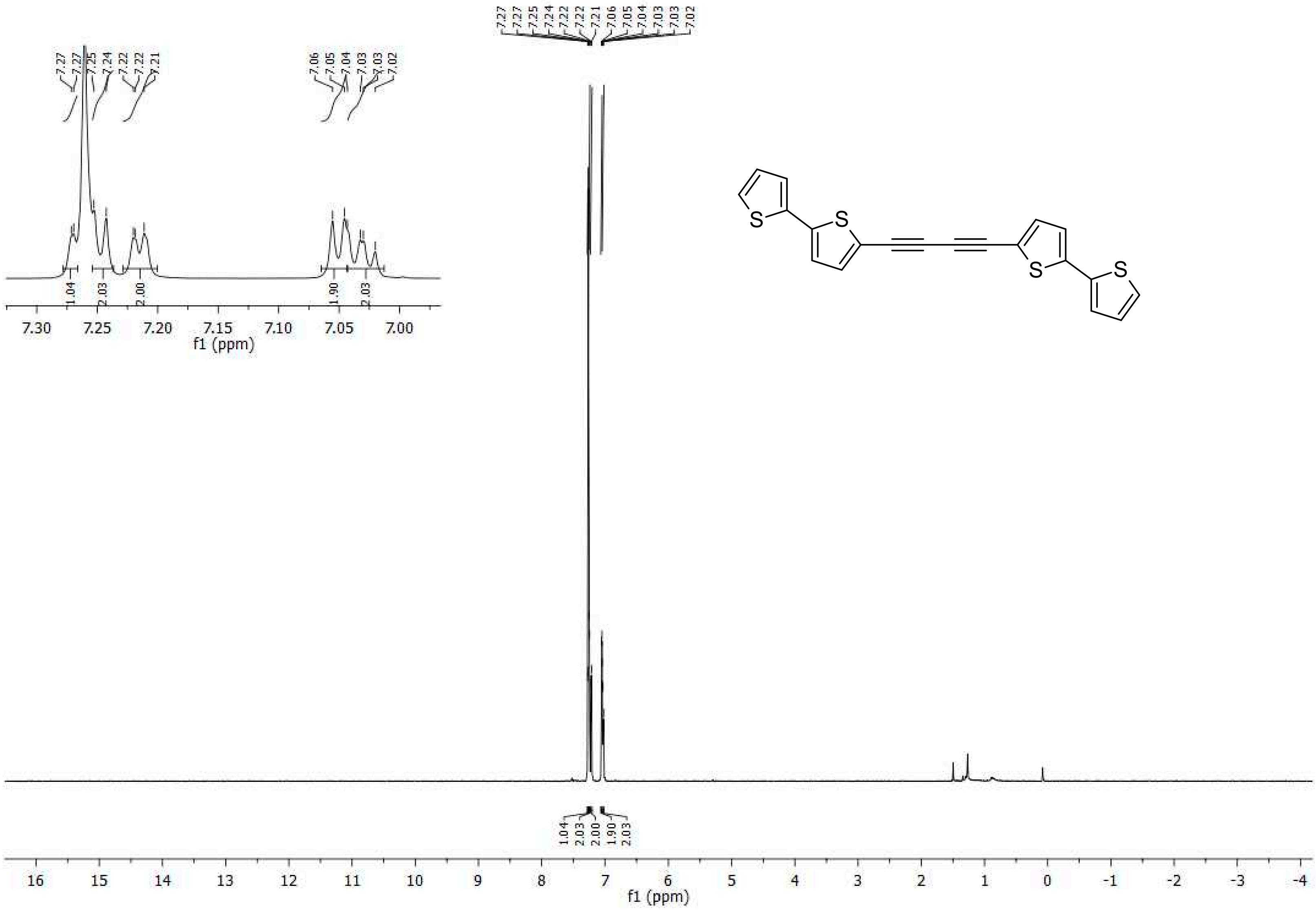

Figure S3. ^{1}H-NMR spectrum of compound (**6**).

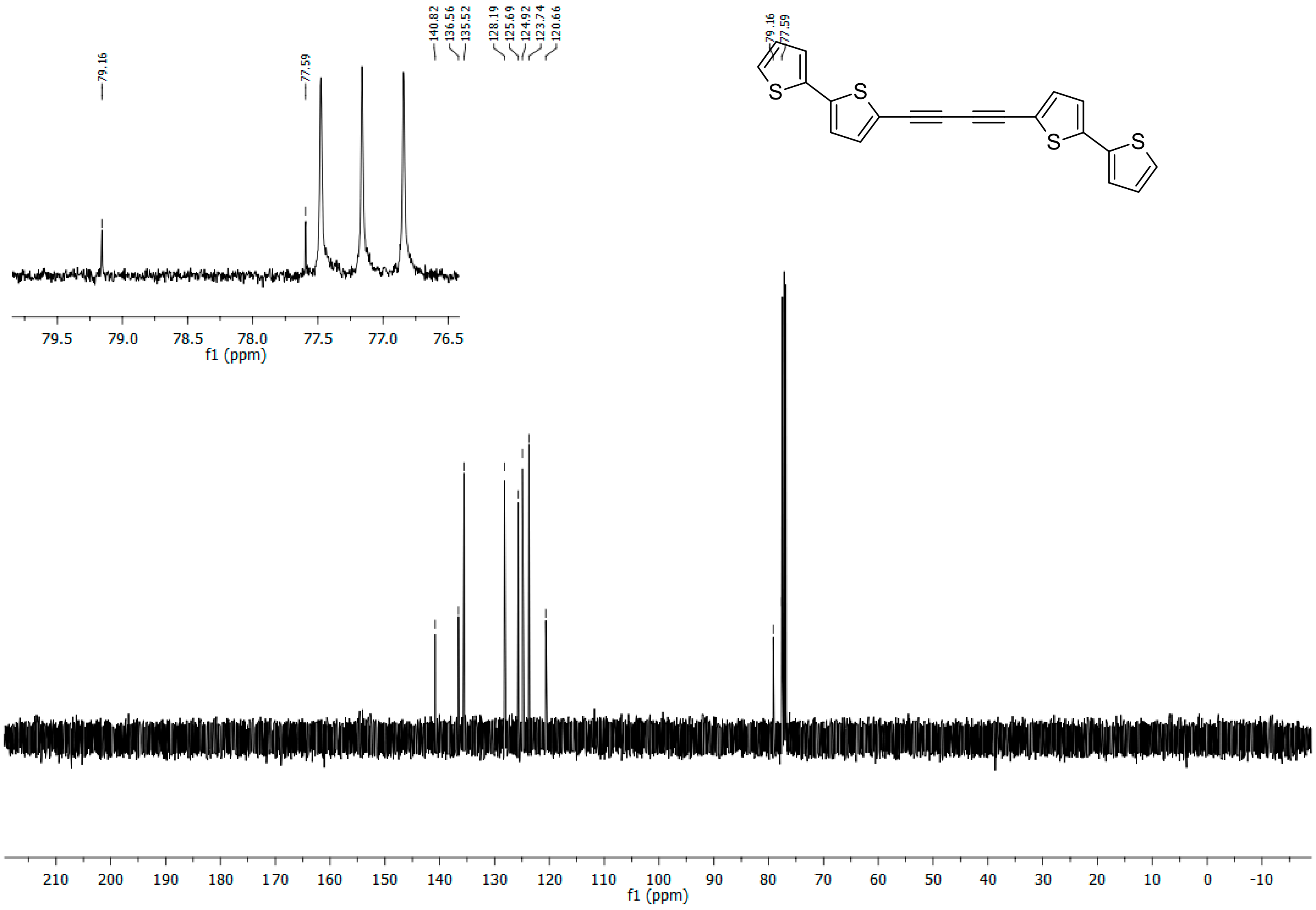

Figure S4. ^{13}C-NMR spectrum of compound (**6**).

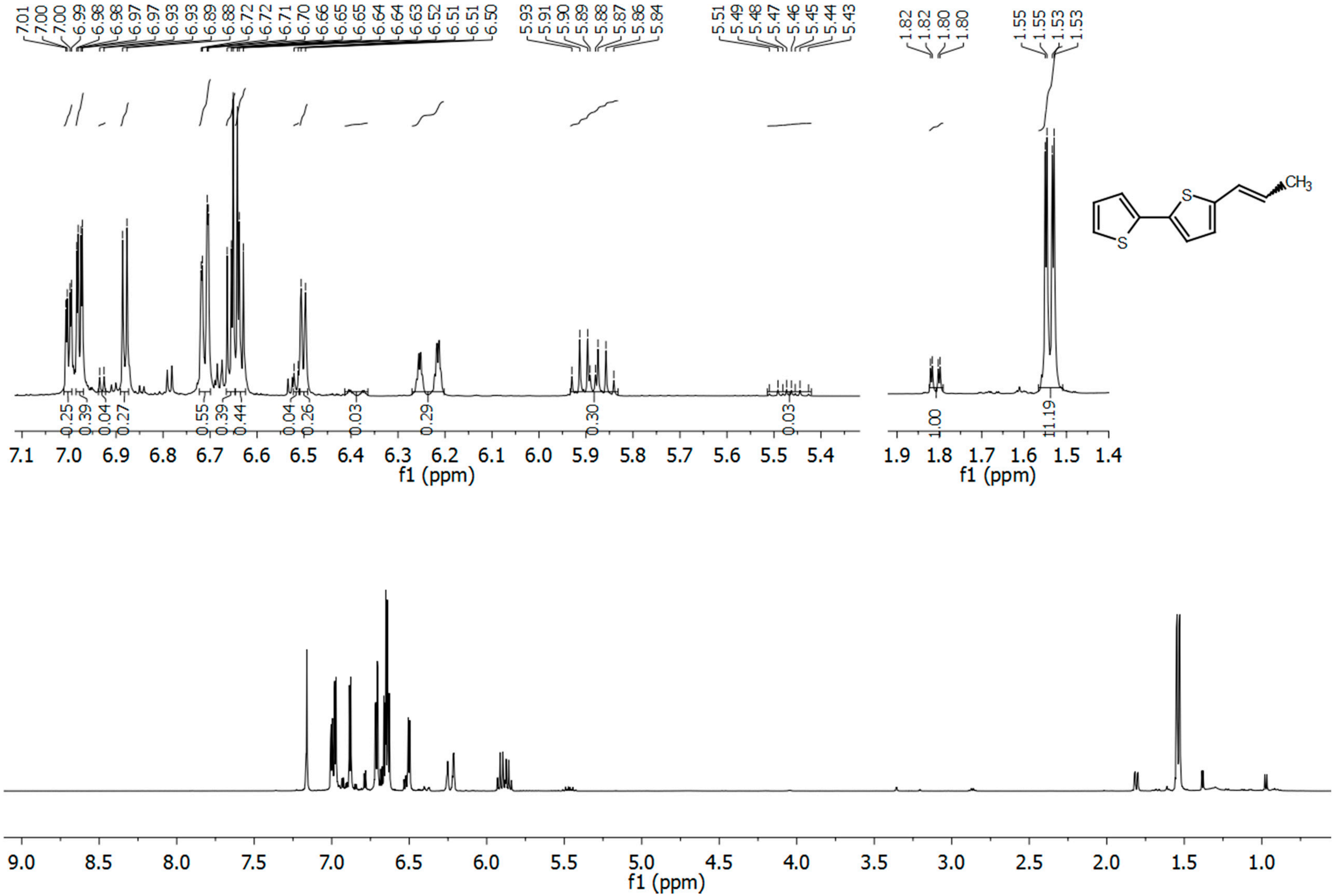

Figure S5. ^{1}H-NMR spectrum of compound (**10**).

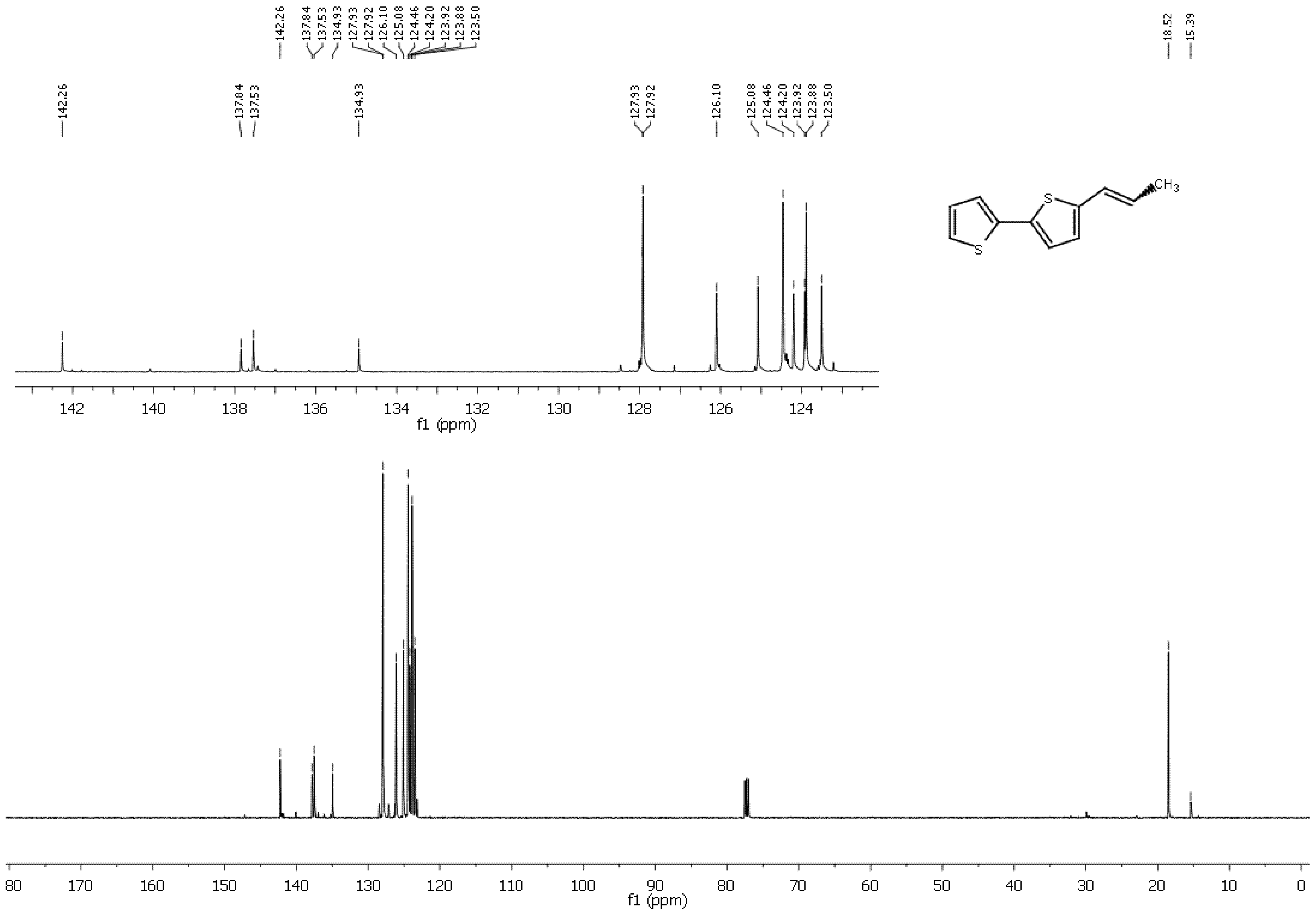

Figure S6. ^{13}C-NMR spectrum of compound (**10**).

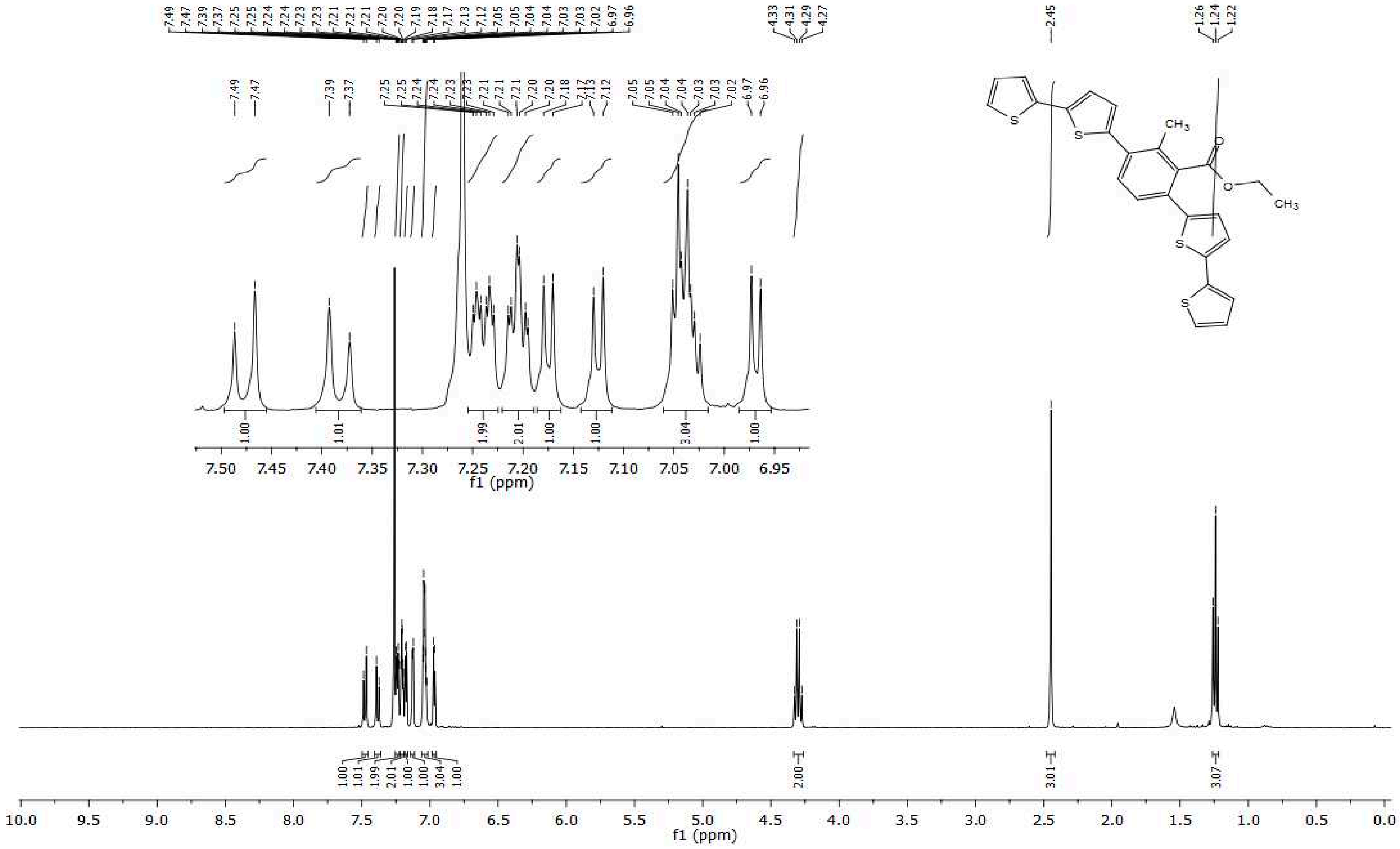

Figure S7. ^{1}H-NMR spectrum of compound (**7**).

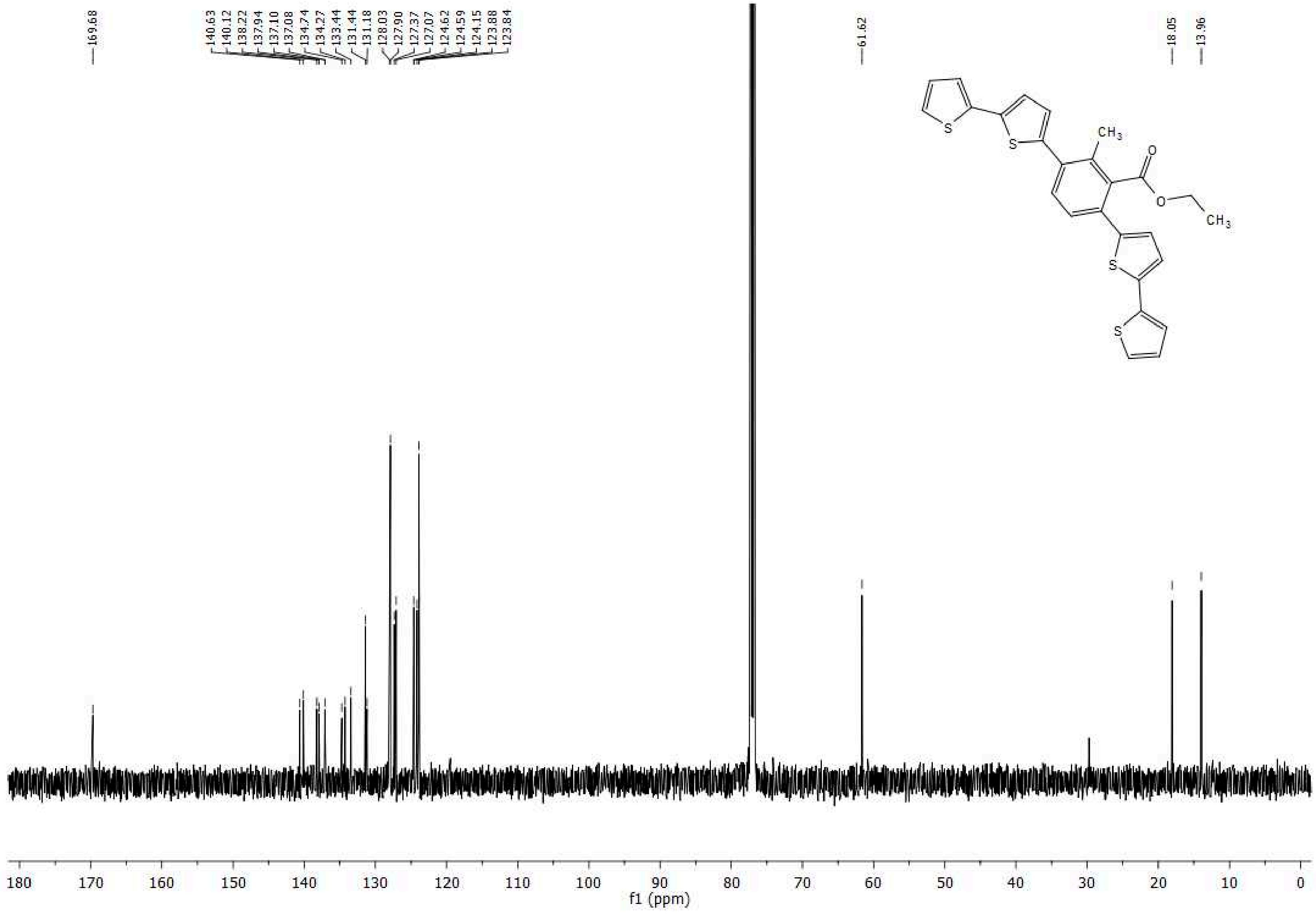

Figure S8. ^{1}H-NMR spectrum of compound (**7**).

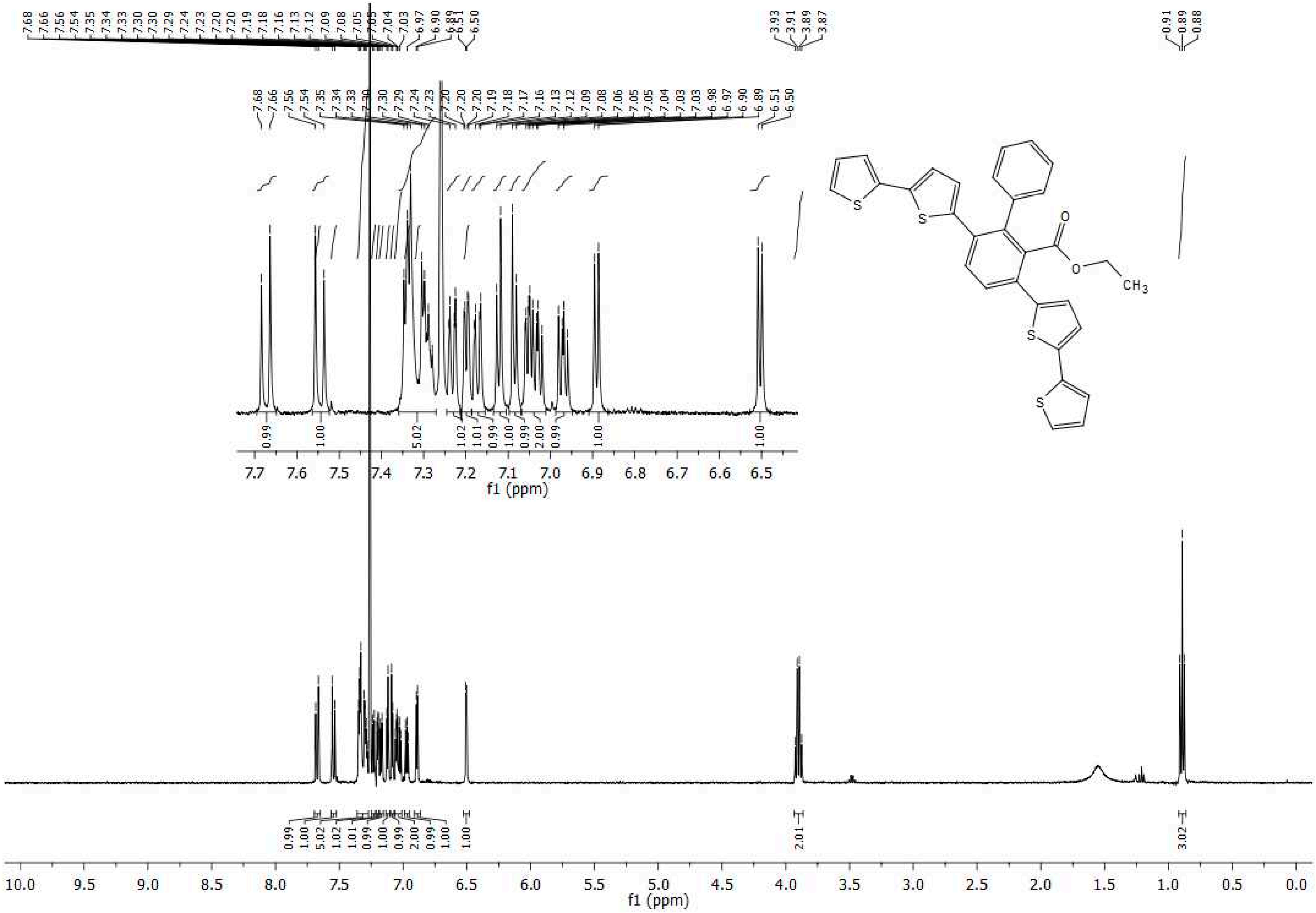

Figure S9. ^{1}H-NMR spectrum of compound (**8**).

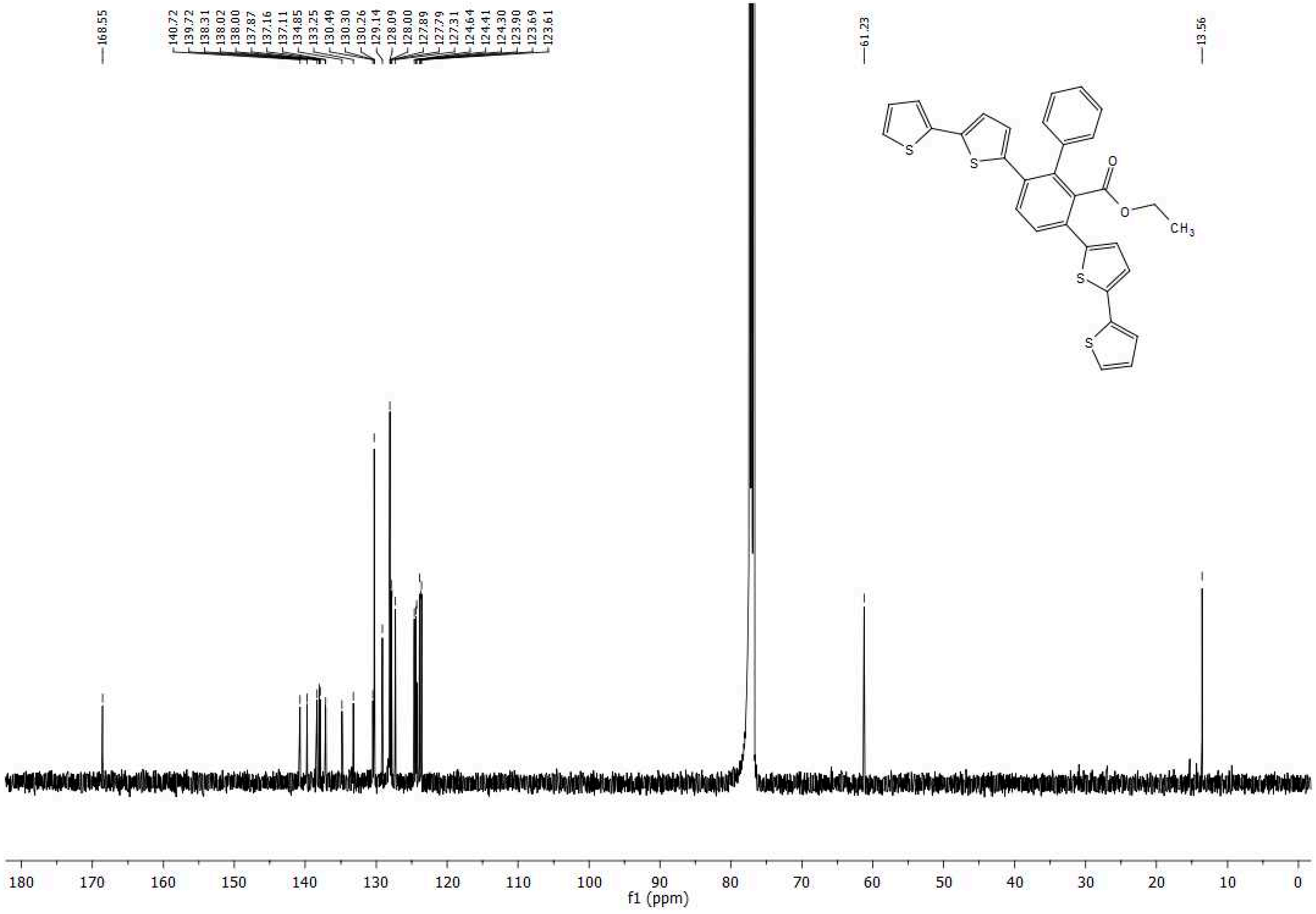

Figure S10. ^{13}C-NMR spectrum of compound (**8**).

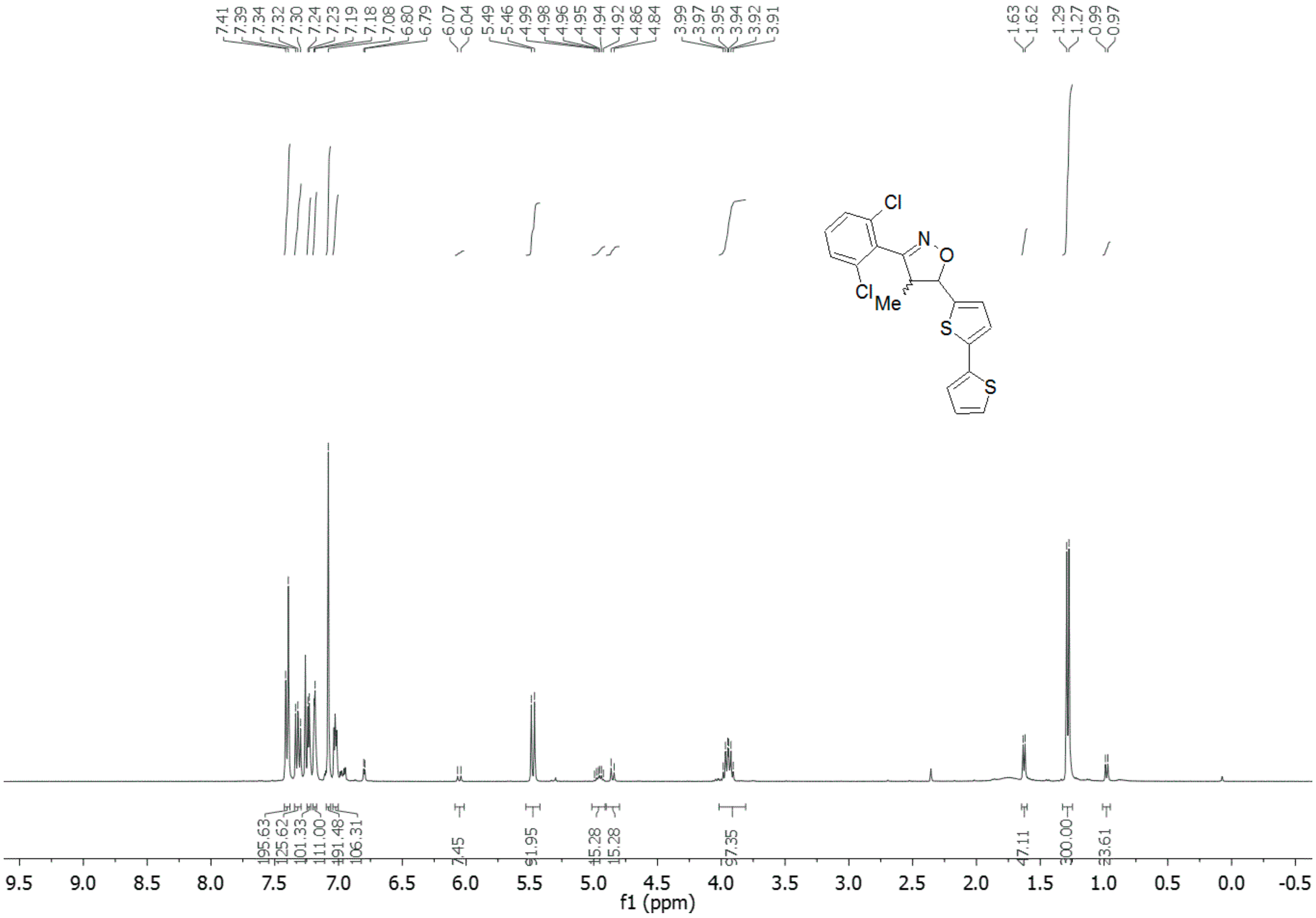

Figure S11. ^{1}H-NMR spectrum of compound (**11**).

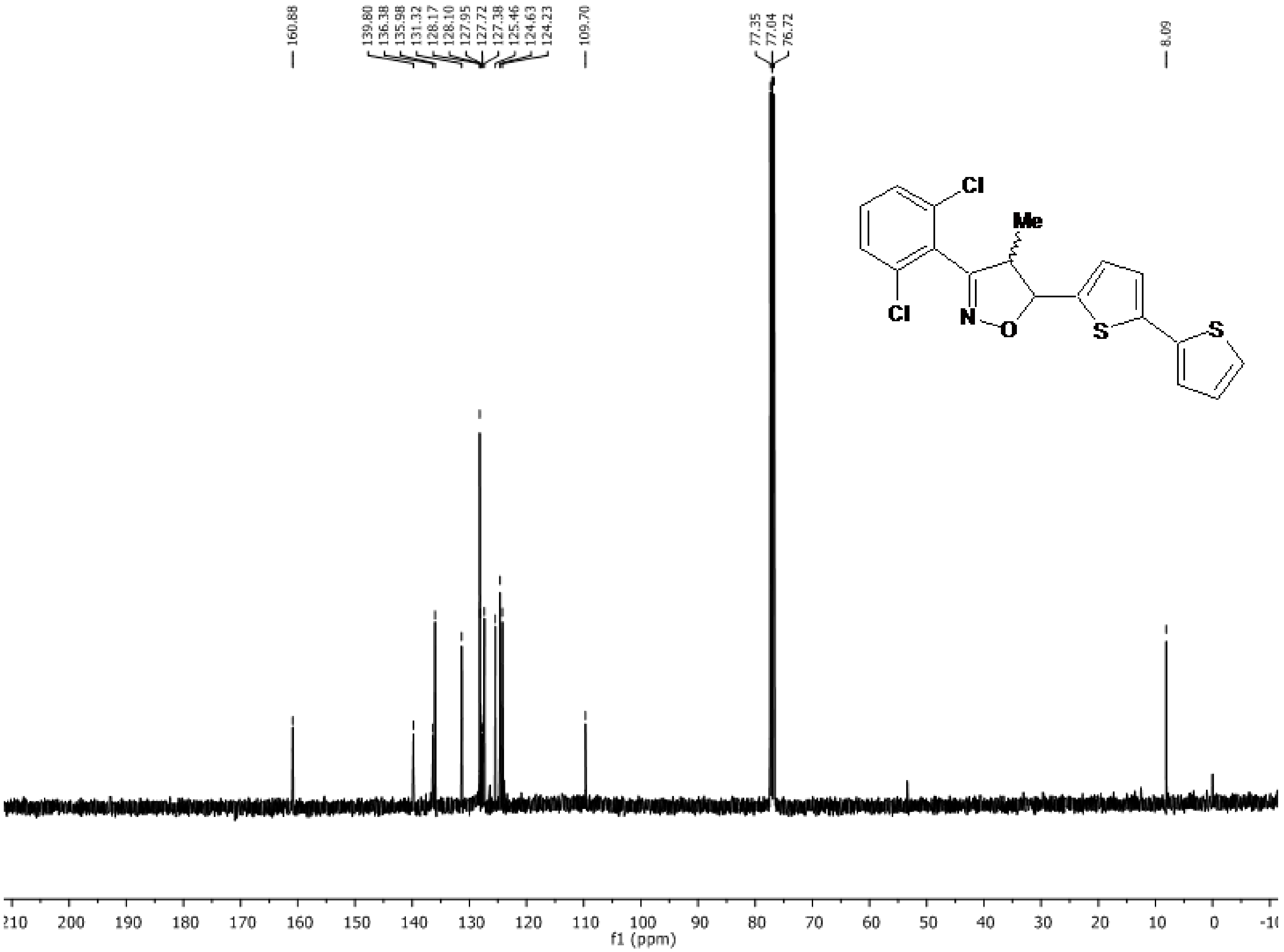

Figure S12. ^{13}C-NMR spectrum of compound (**11**).

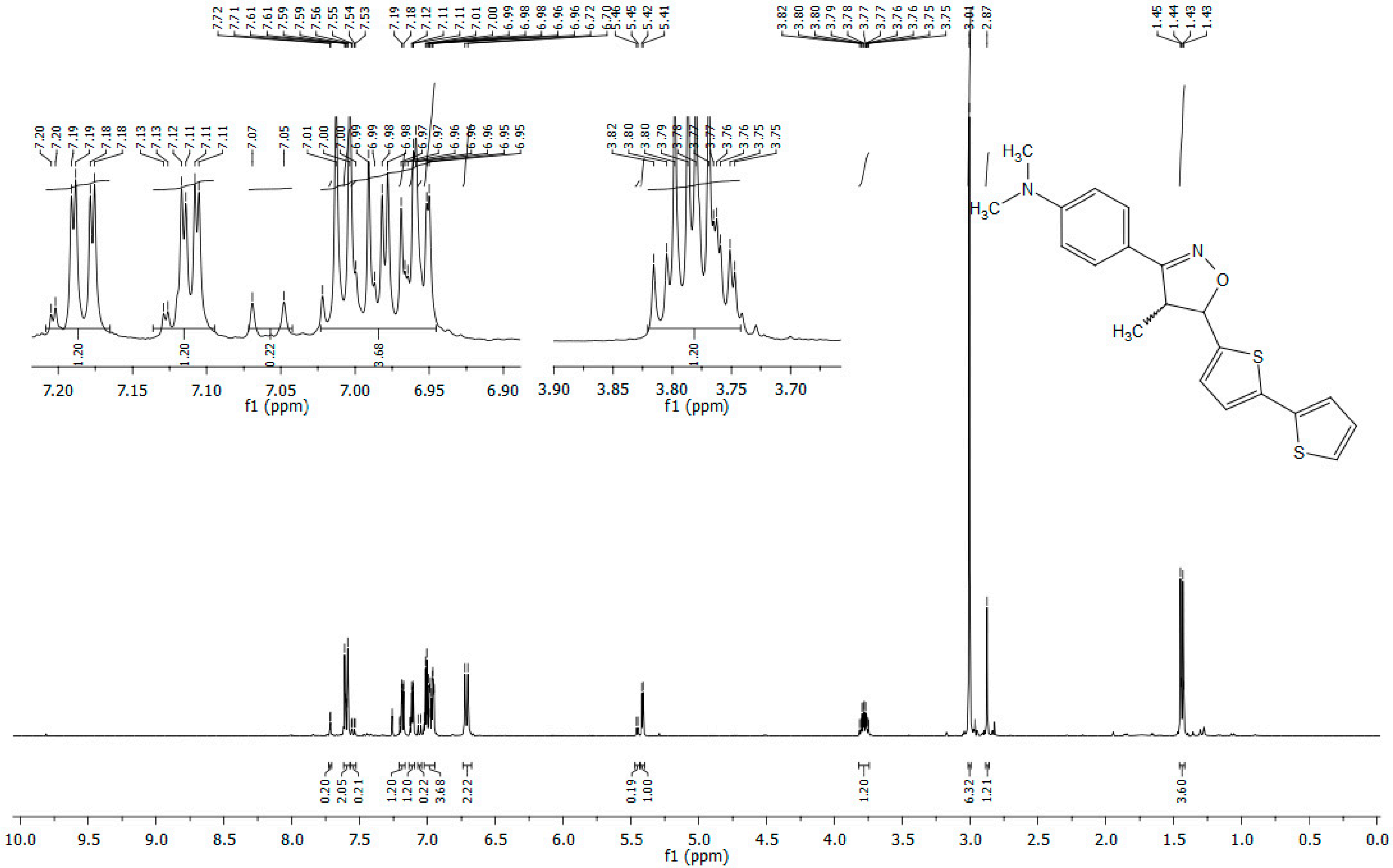

Figure S13. ^{1}H-NMR spectrum of compound (**12**).

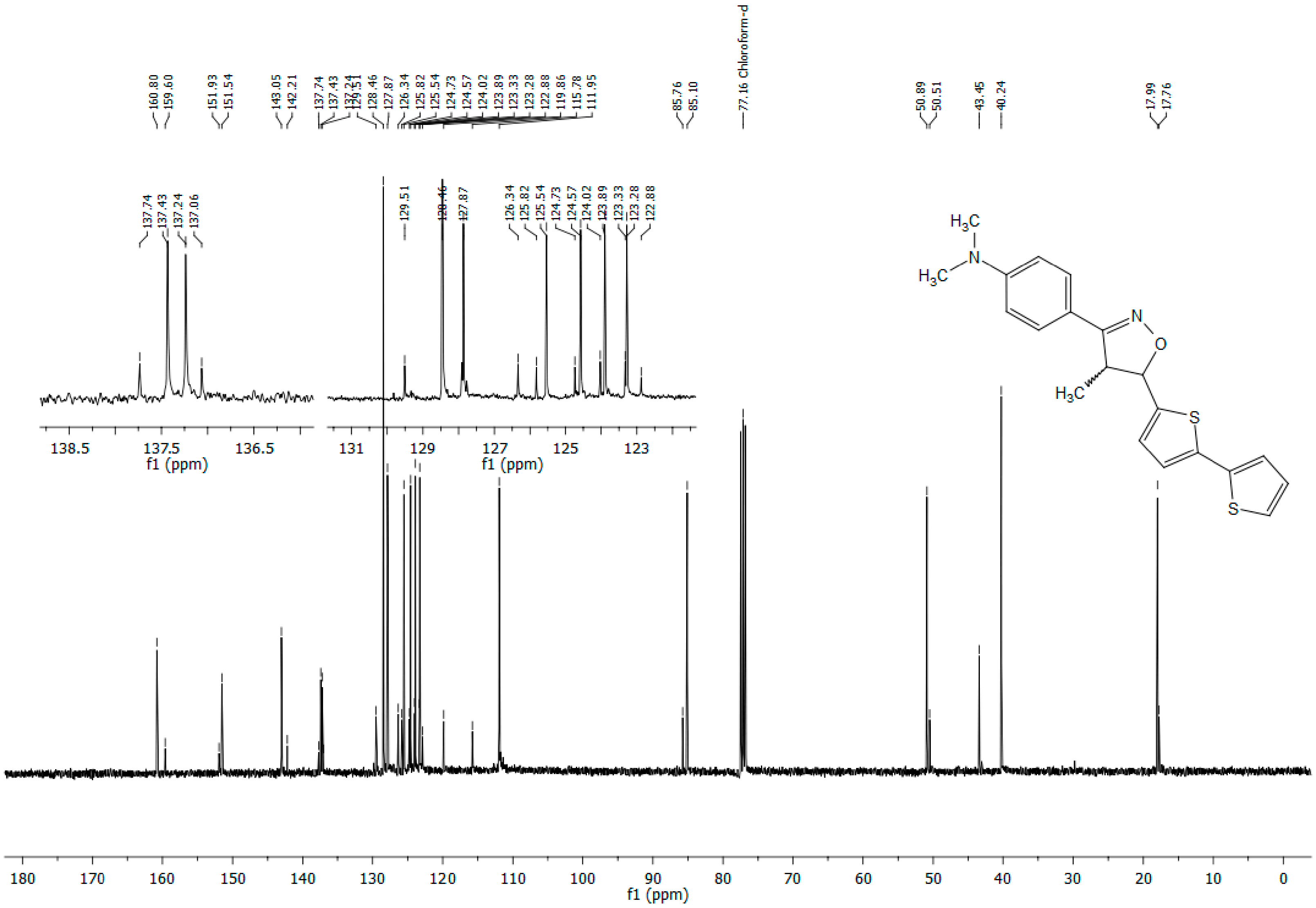

Figure S14. ^{13}C-NMR spectrum of compound (**12**).

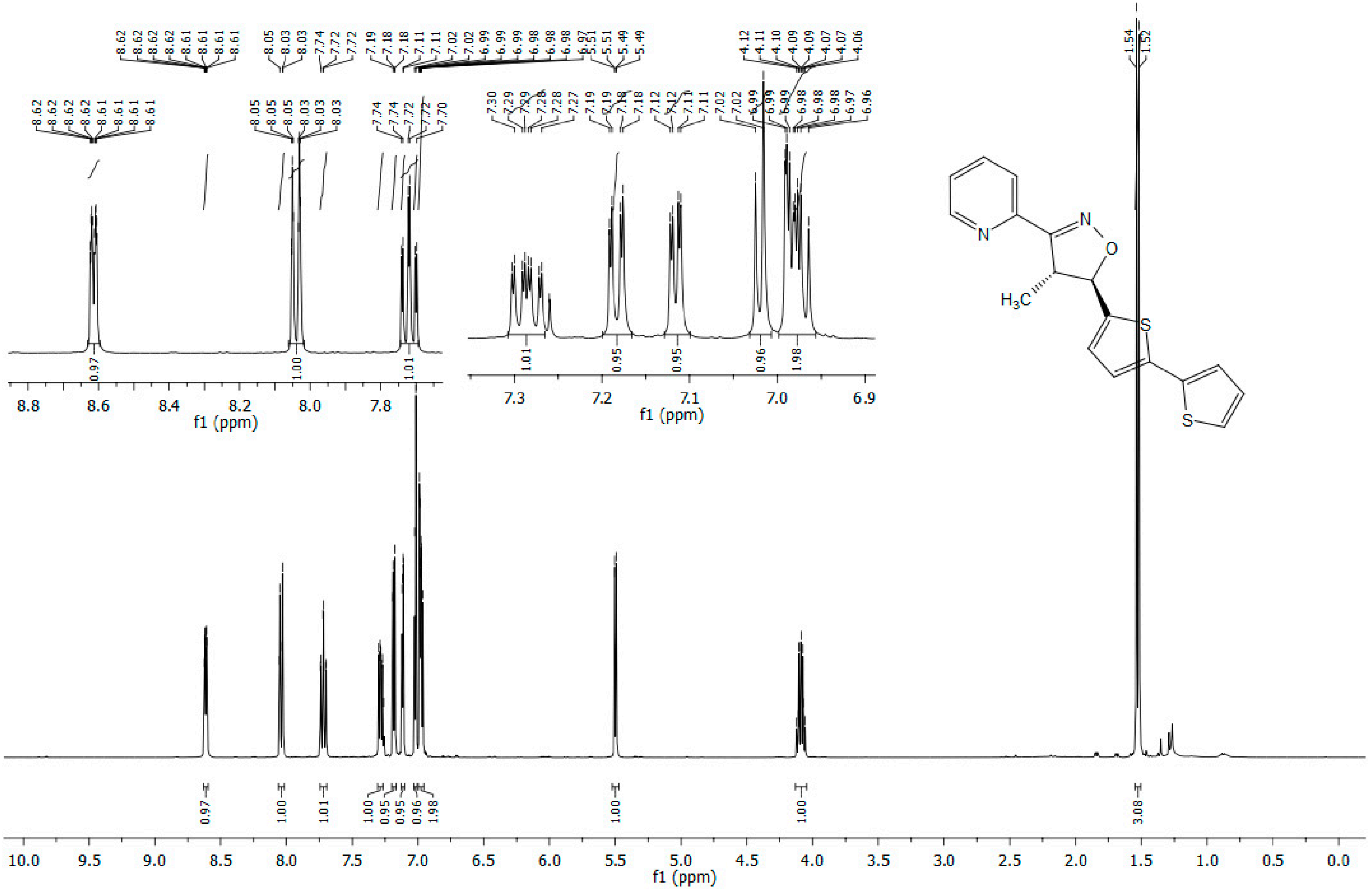

Figure S15. ^{1}H-NMR spectrum of compound (**13**).

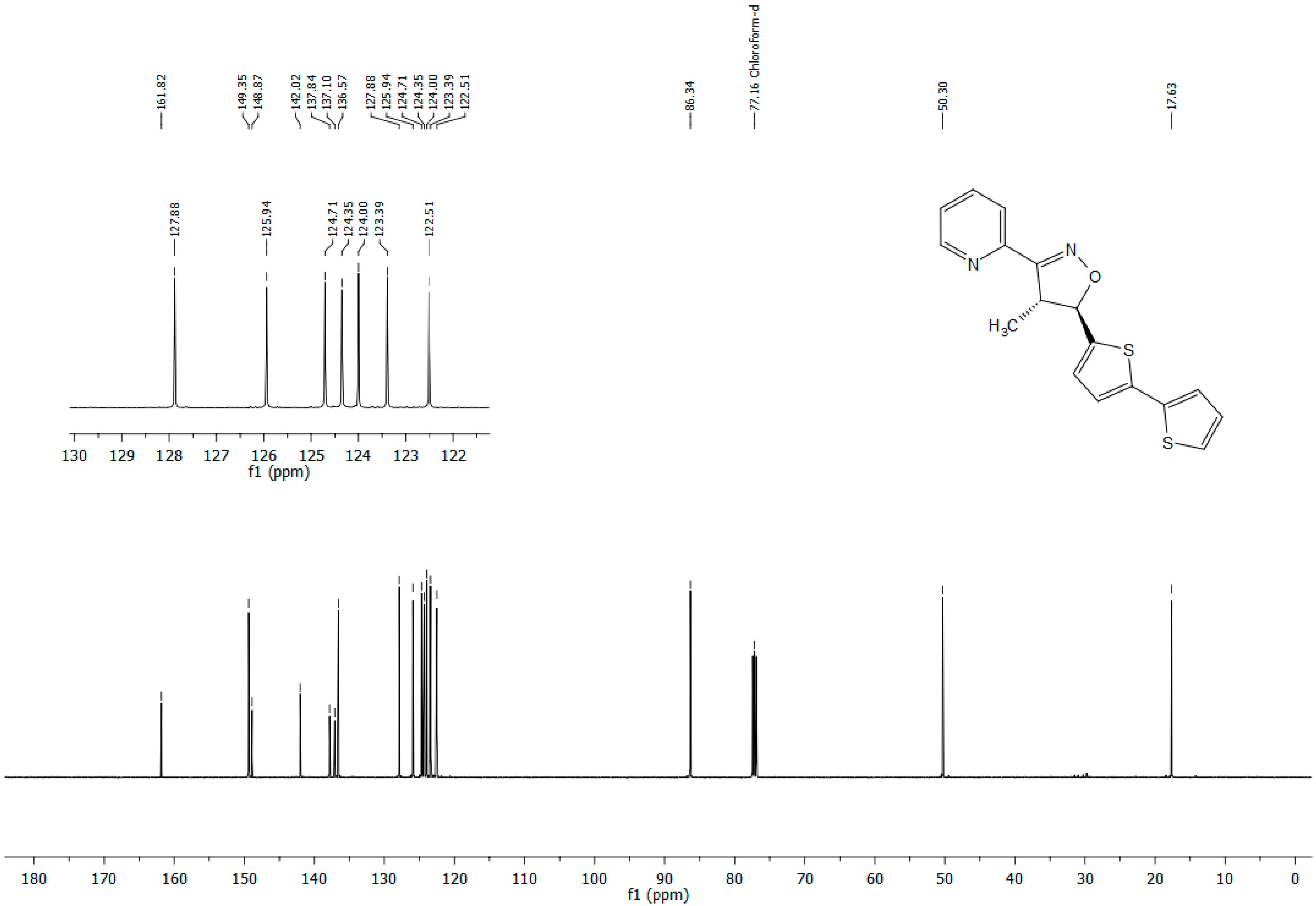

Figure S16. ^{13}C-NMR spectrum of compound (**13**).

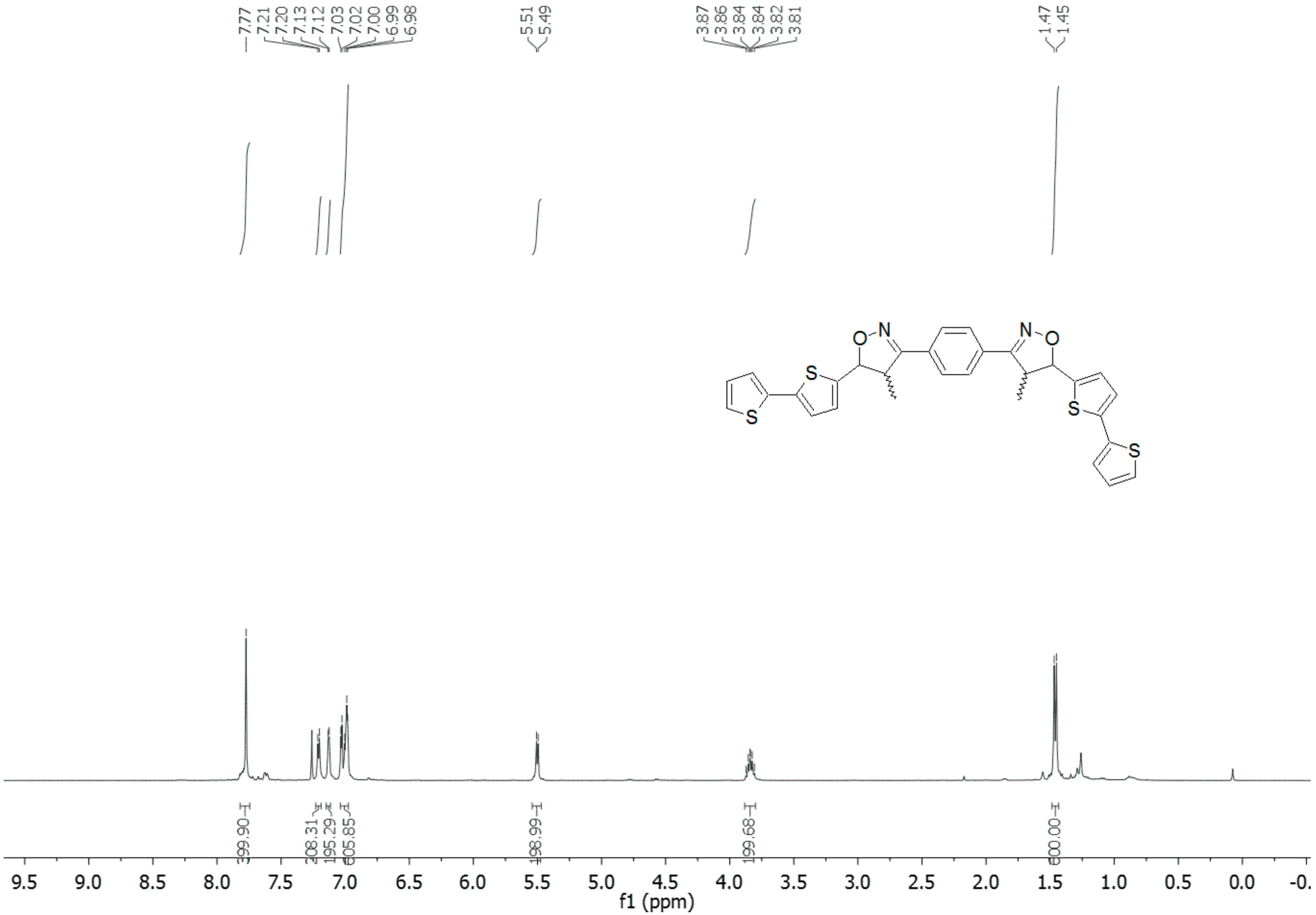

Figure S17. ^{1}H-NMR spectrum of compound (**18**).

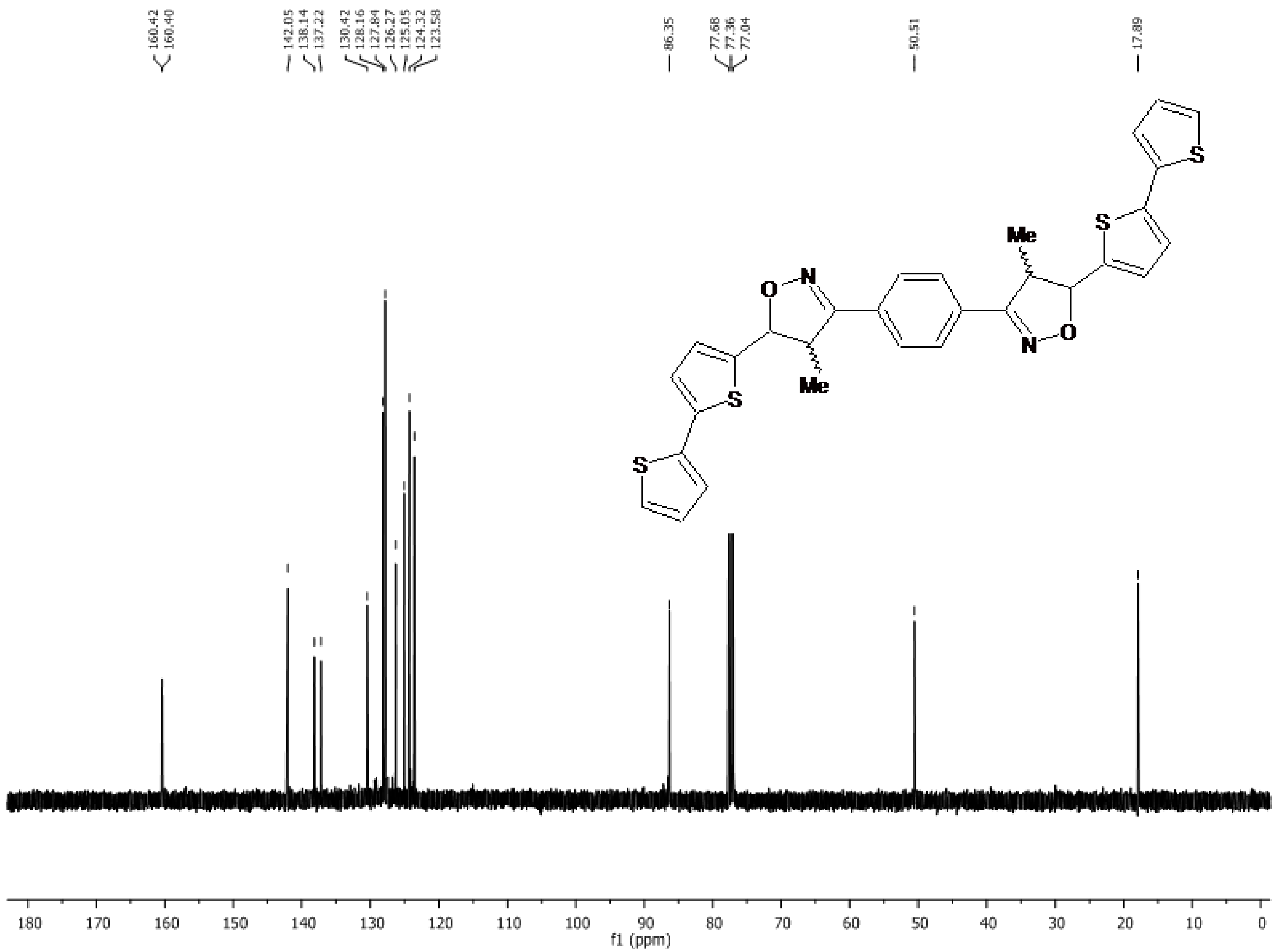

Figure S18. ^{13}C-NMR spectrum of compound (**18**).

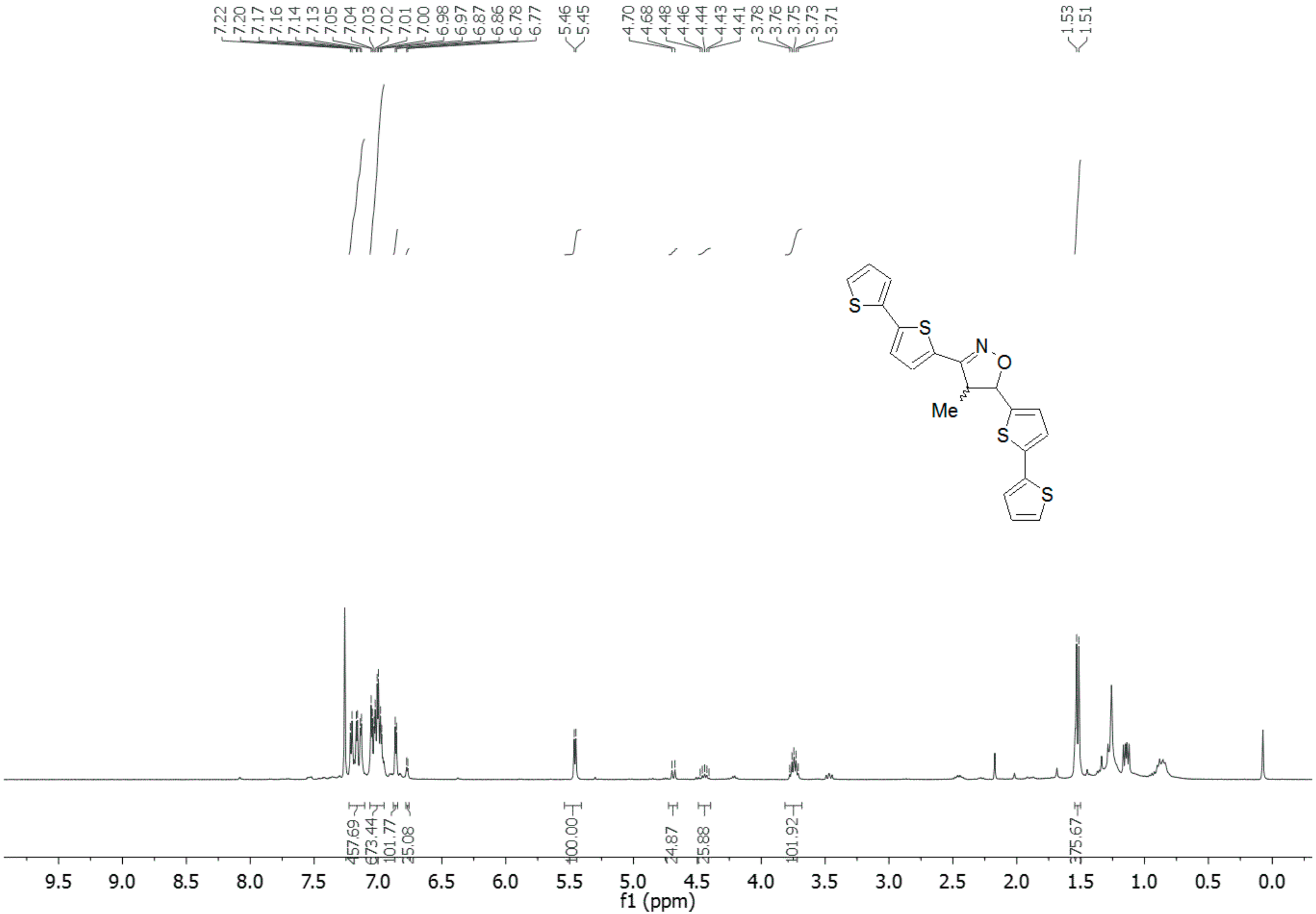

Figure S19. ^{1}H-NMR spectrum of compound (**14**).

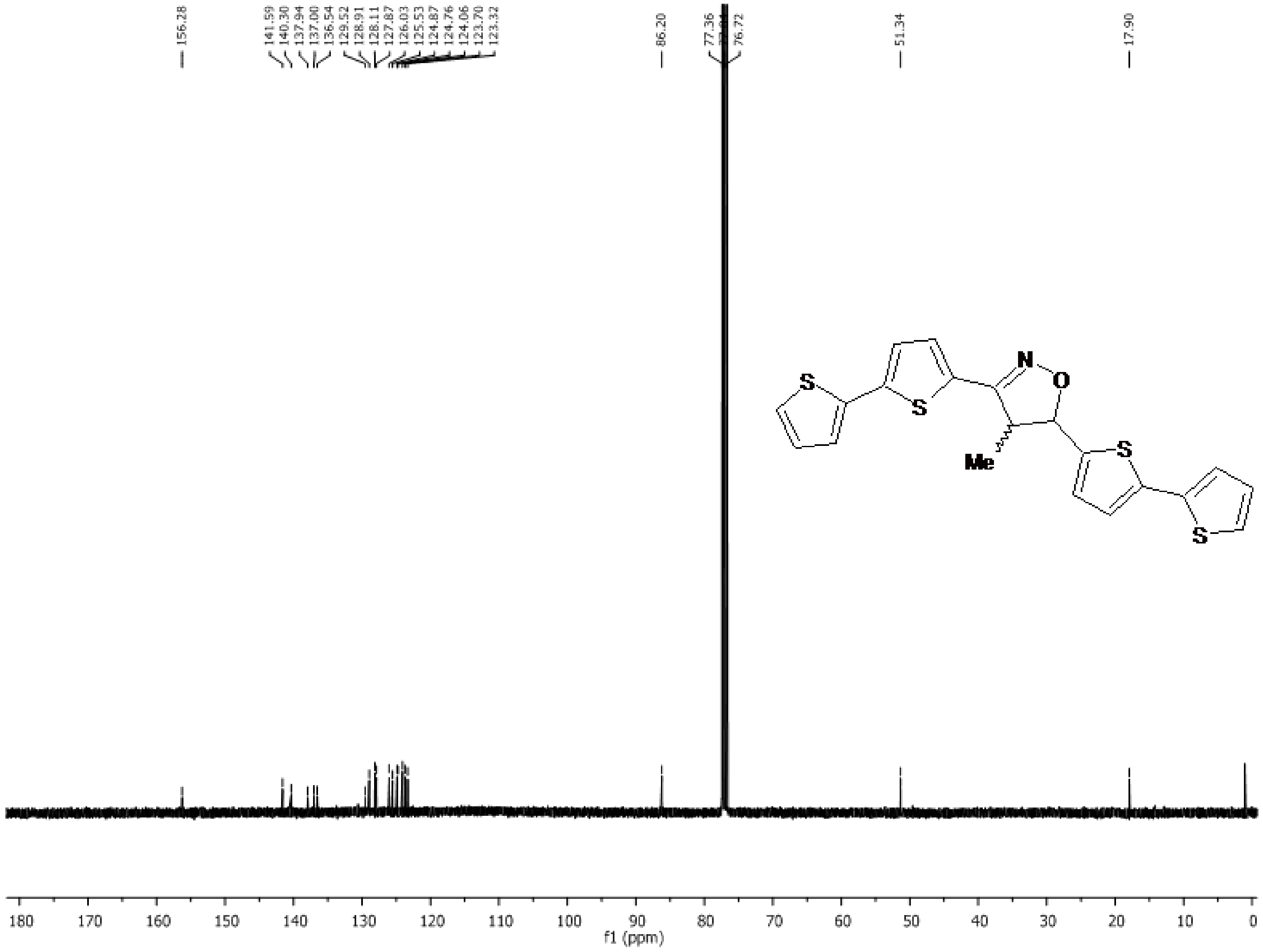

Figure S20. ^{13}C-NMR spectrum of compound (**14**).

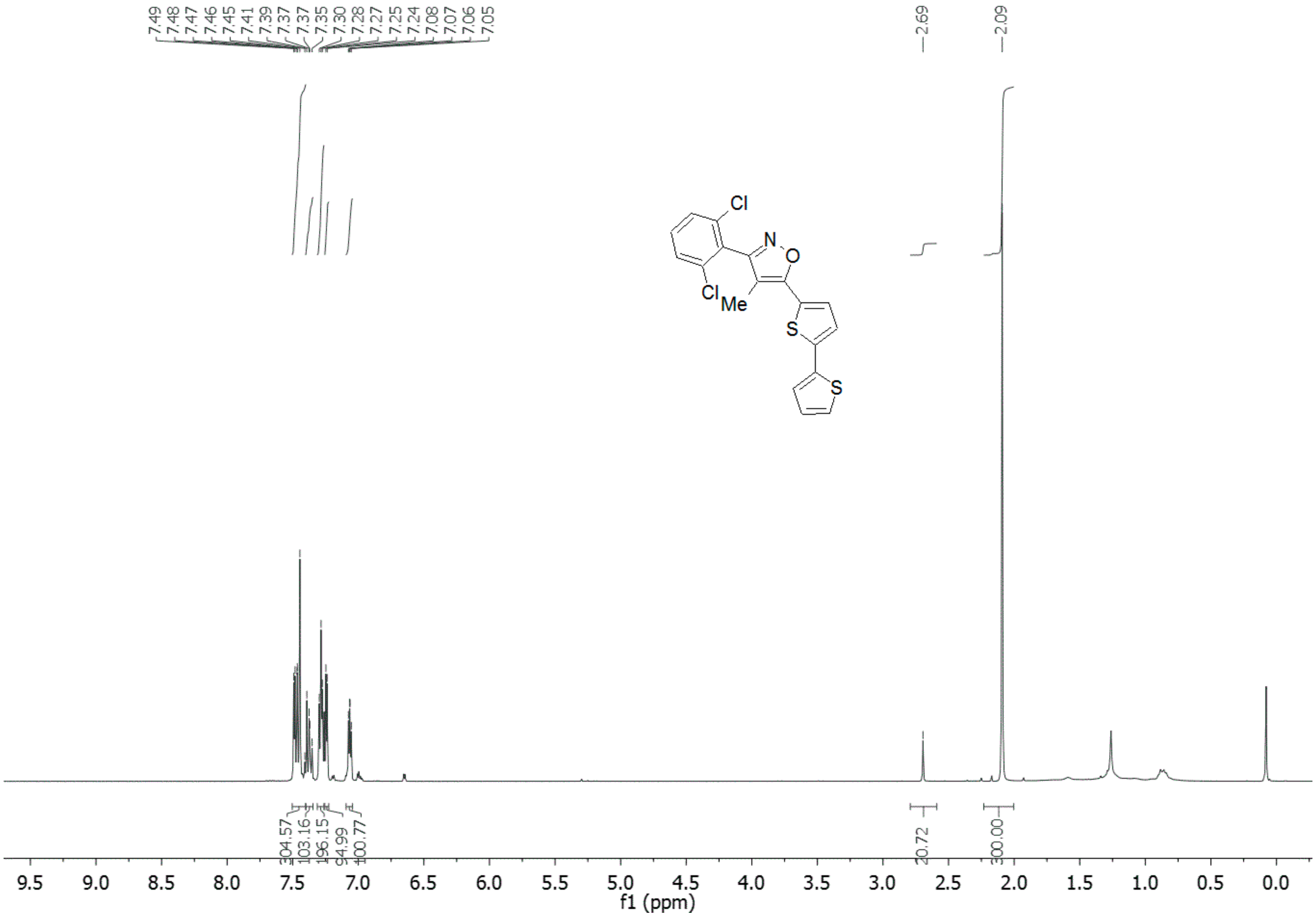

Figure S21. ^{1}H-NMR spectrum of compound (**16**).

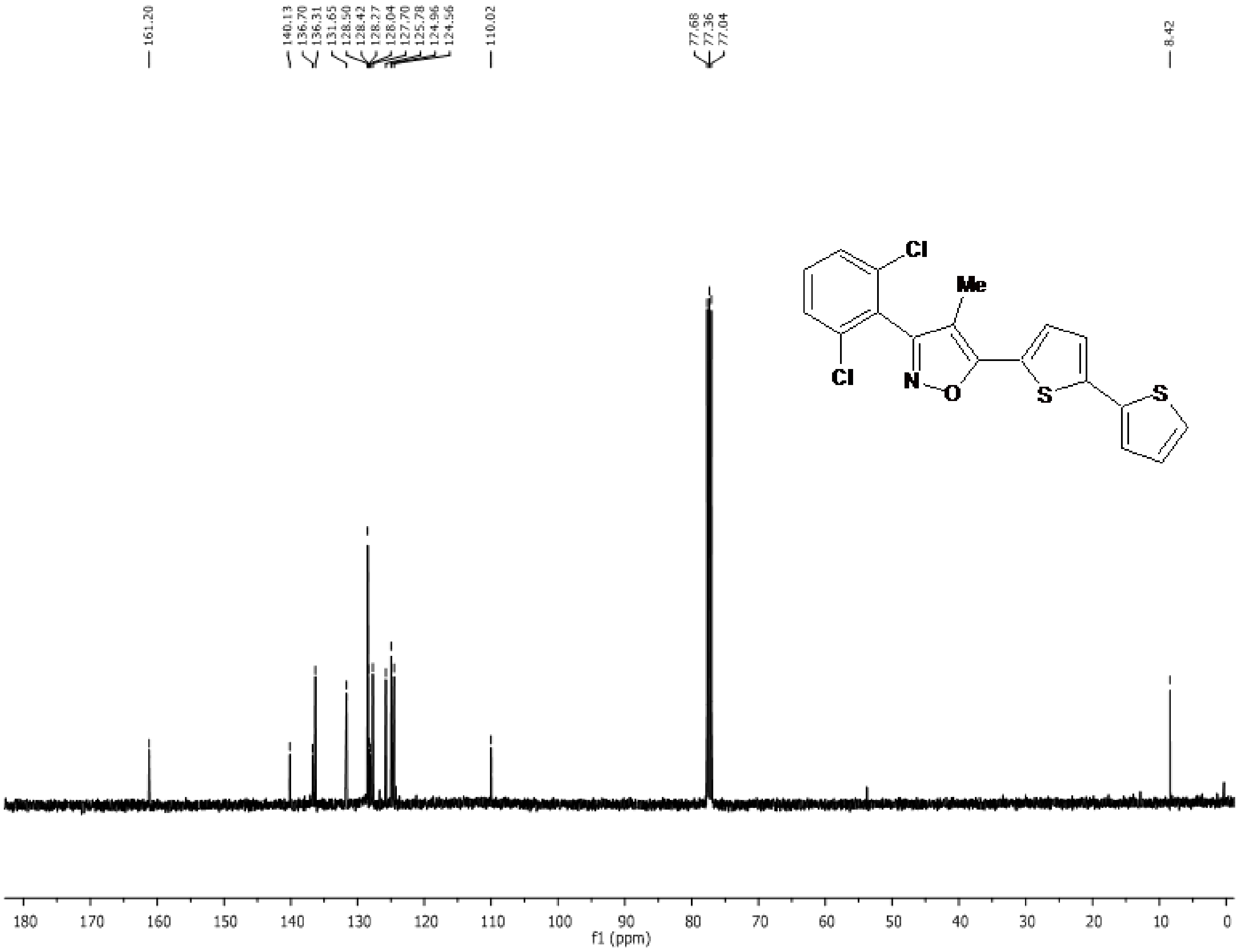

Figure S22. ^{13}C-NMR spectrum of compound (**16**).

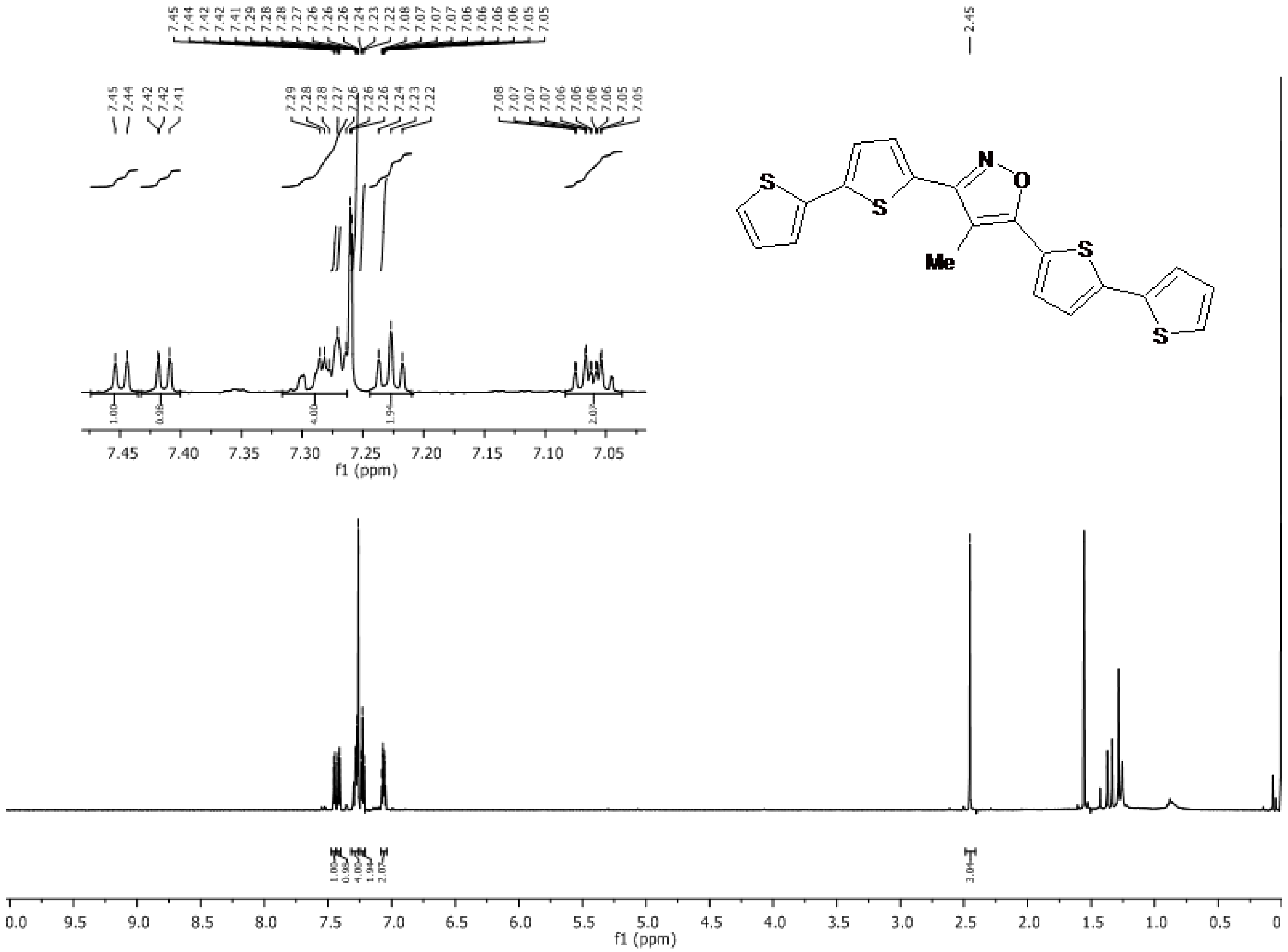

Figure S23. ^{1}H-NMR spectrum of compound (**17**).

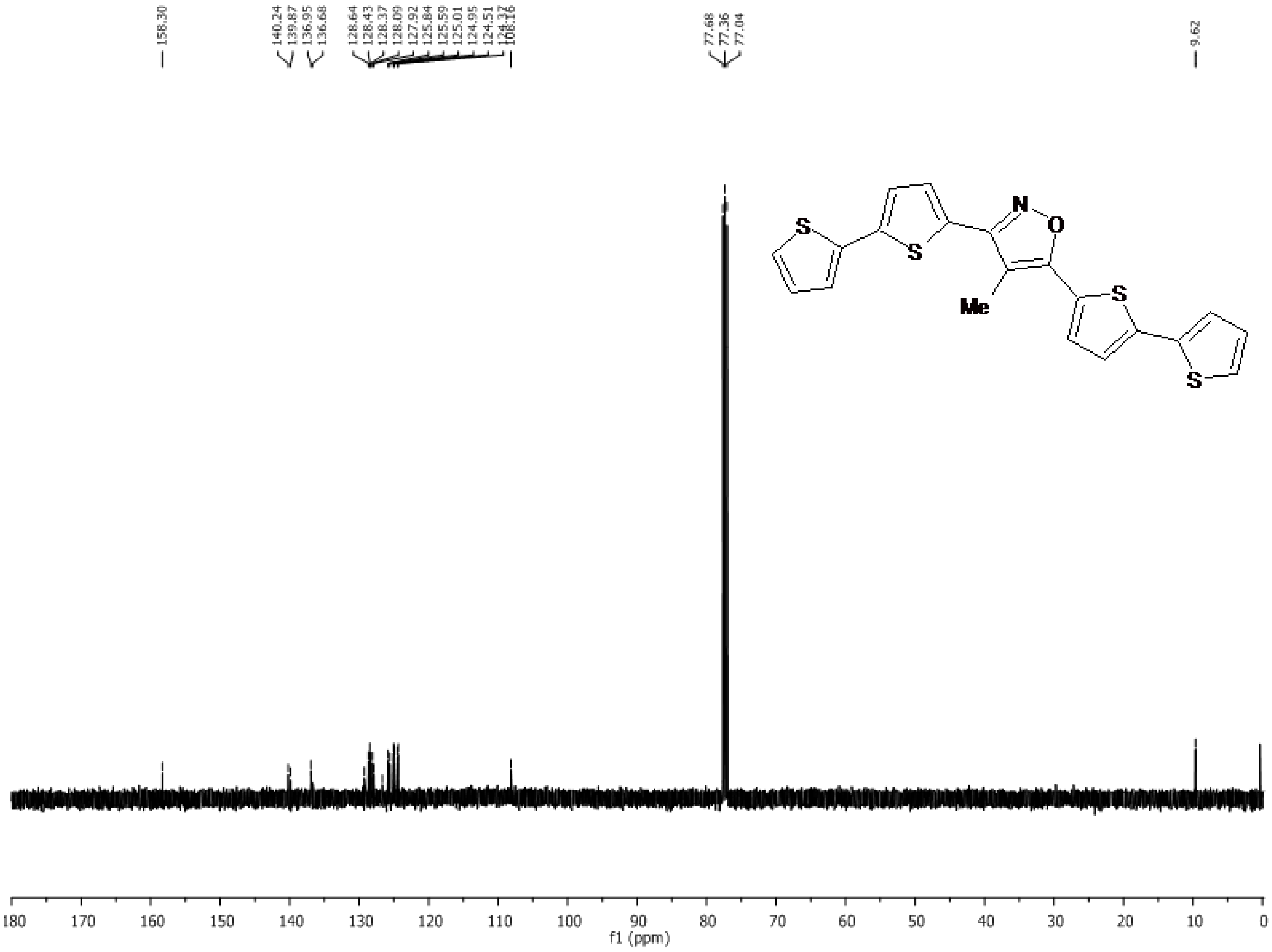

Figure S24. ^{13}C-NMR spectrum of compound (**17**).

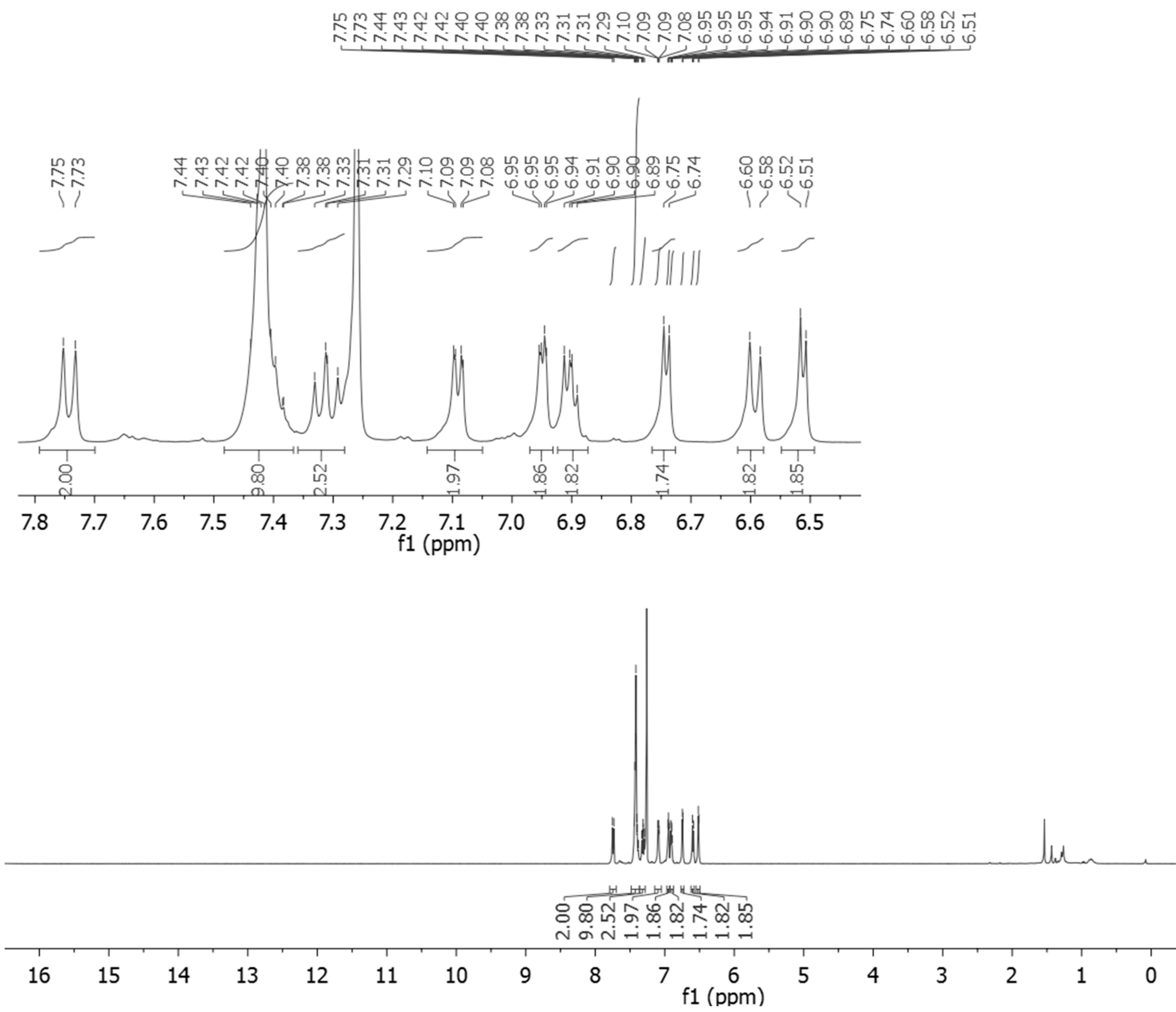

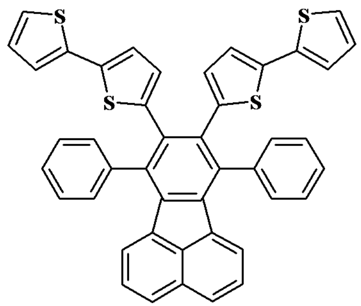

Figure S25. ^{1}H-NMR spectrum of compound (**19**).

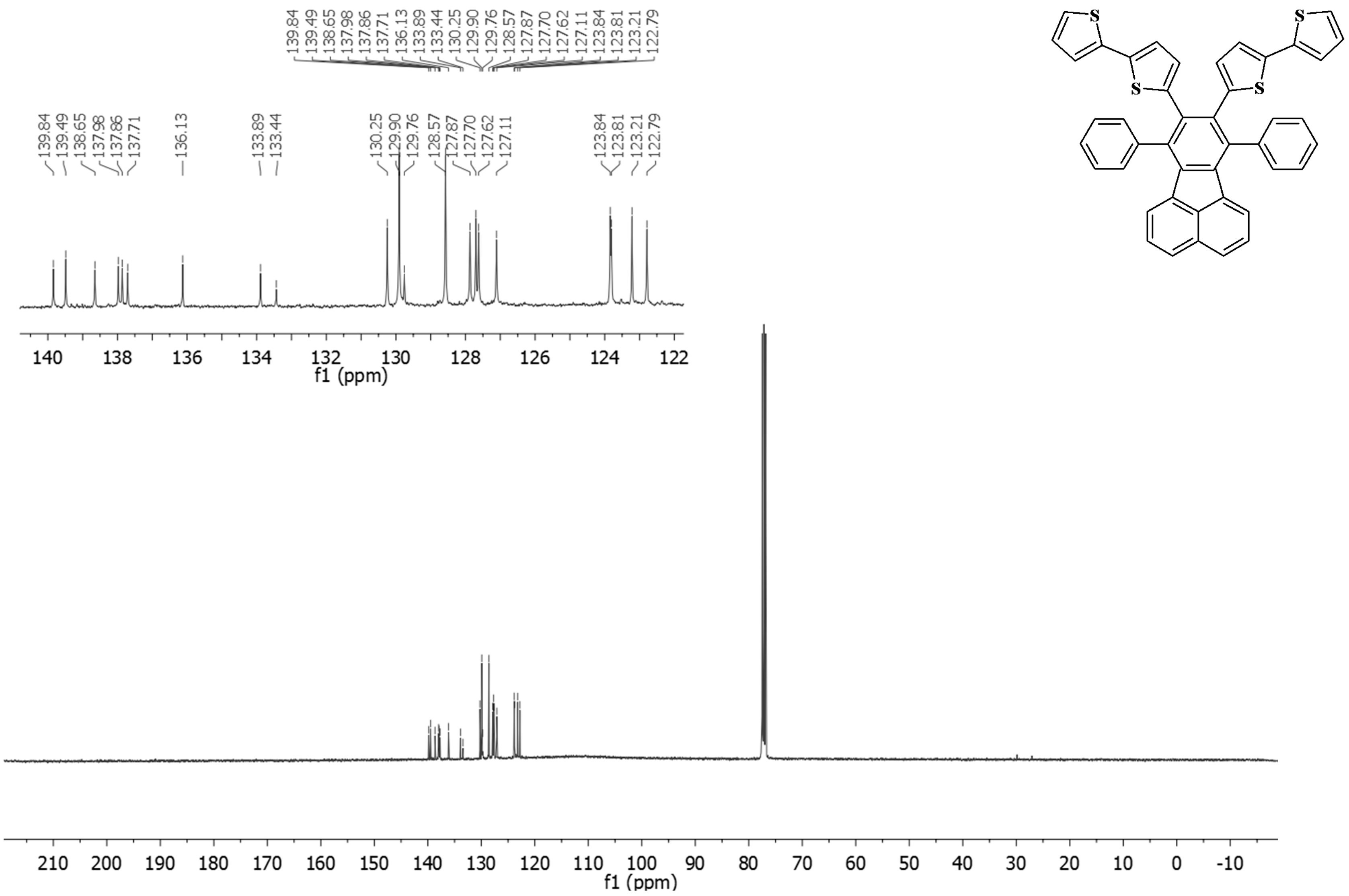

Figure S26. ^{13}C-NMR spectrum of compound (**19**).

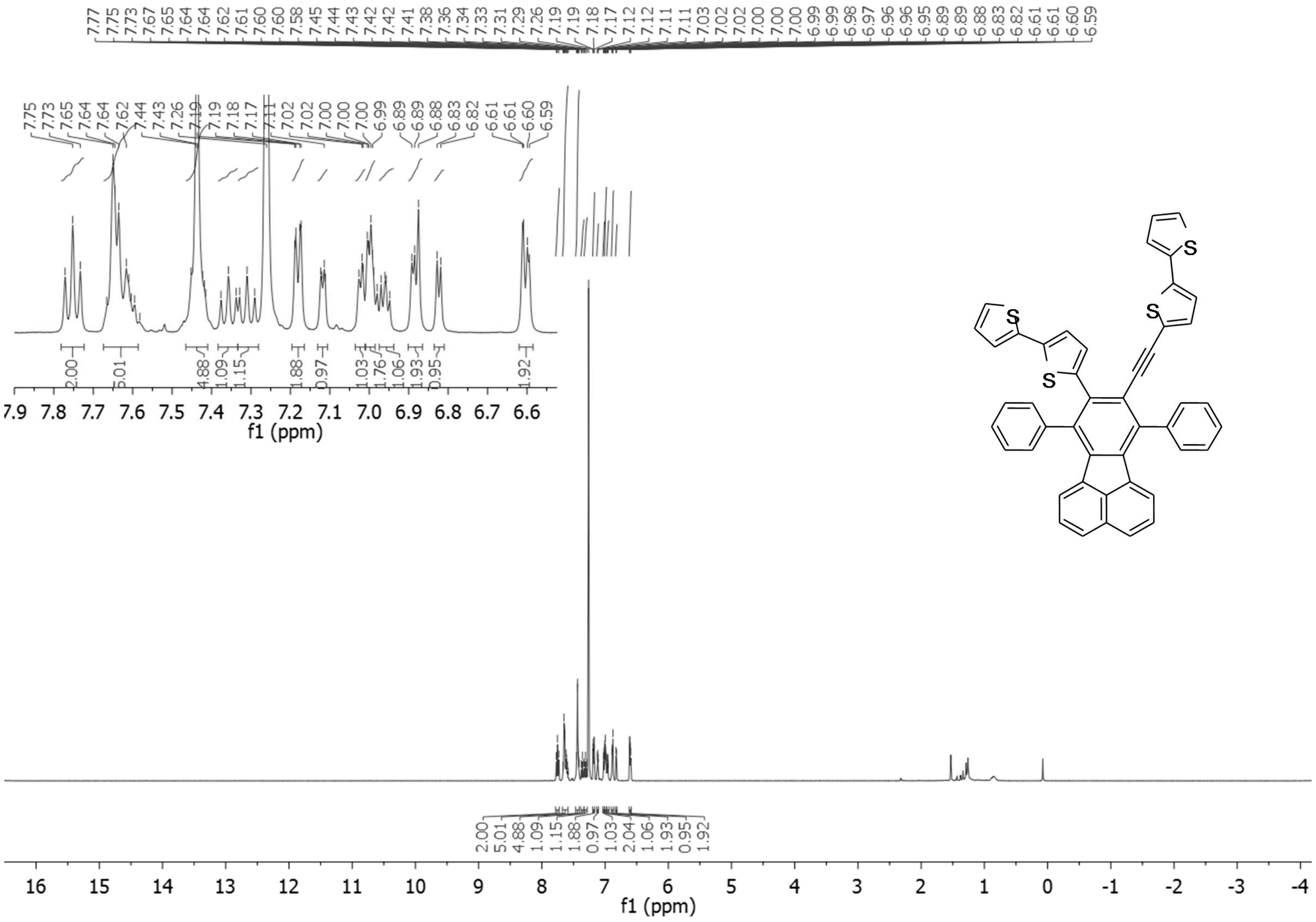

Figure S27. ^{1}H-NMR spectrum of compound (**20**).

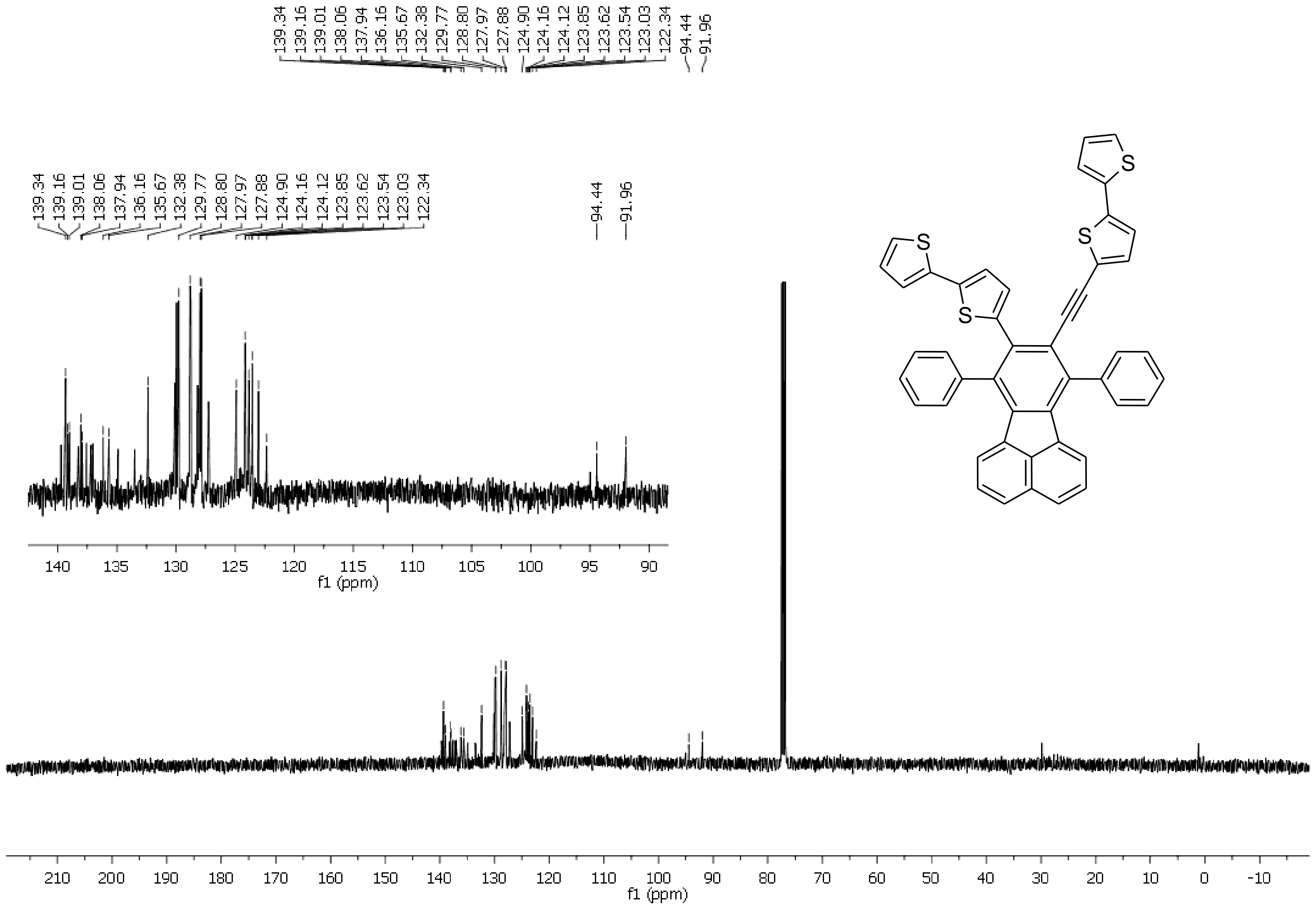

Figure S28. ^{13}C-NMR spectrum of compound (**20**).

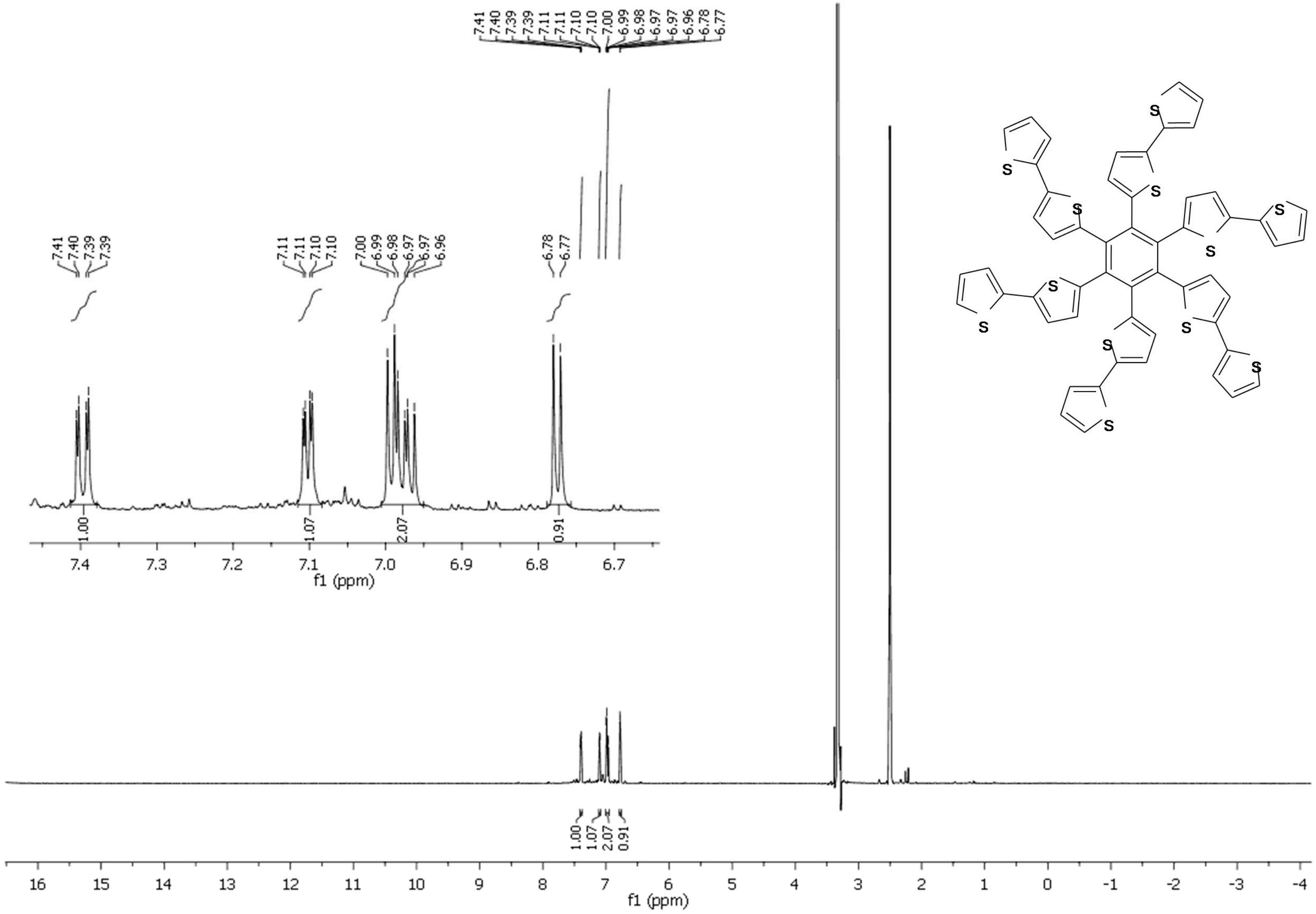

Figure S29. ^{1}H-NMR spectrum of compound (**21**).

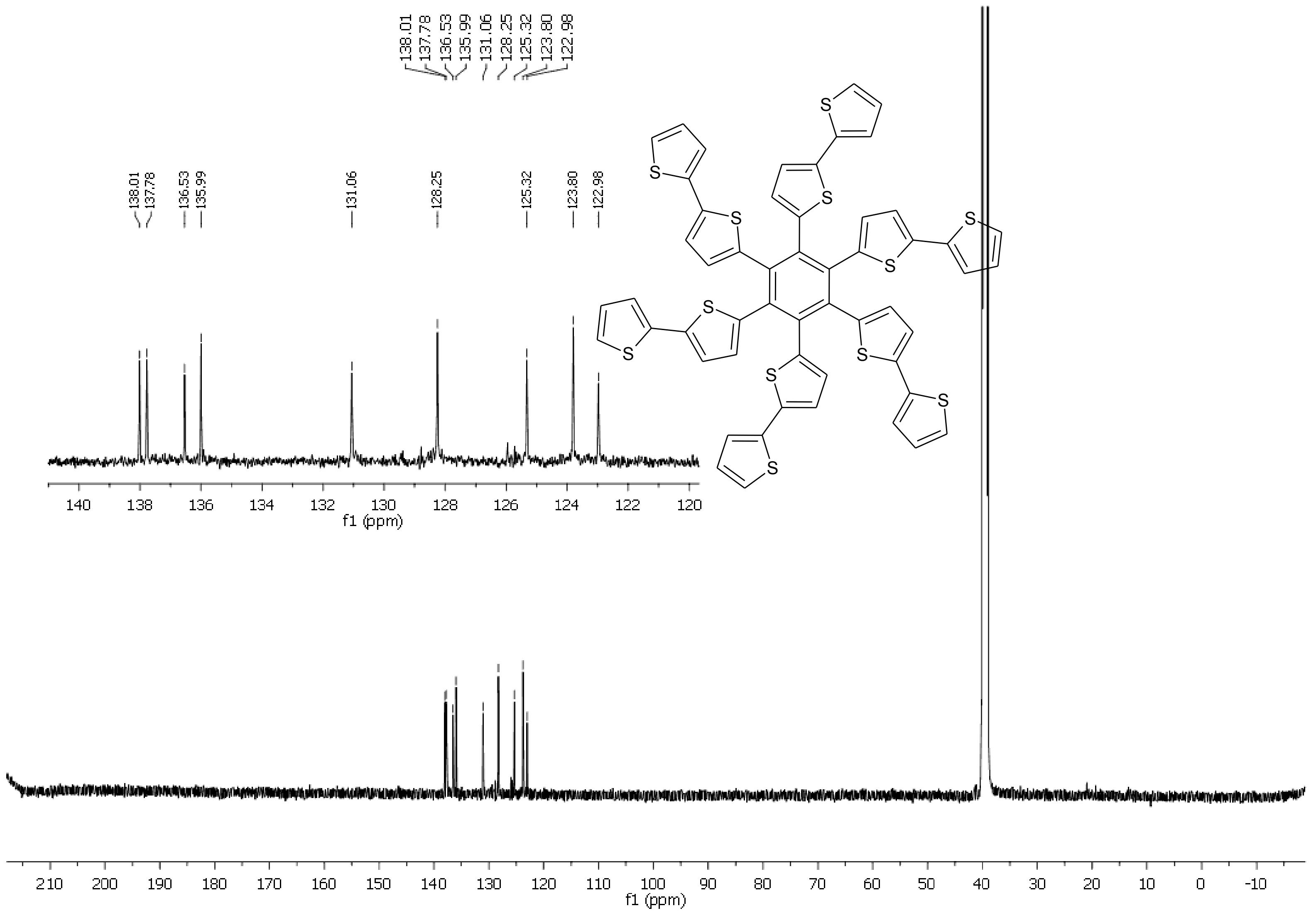

Figure S30. ^{1}H-NMR spectrum of compound (**21**).

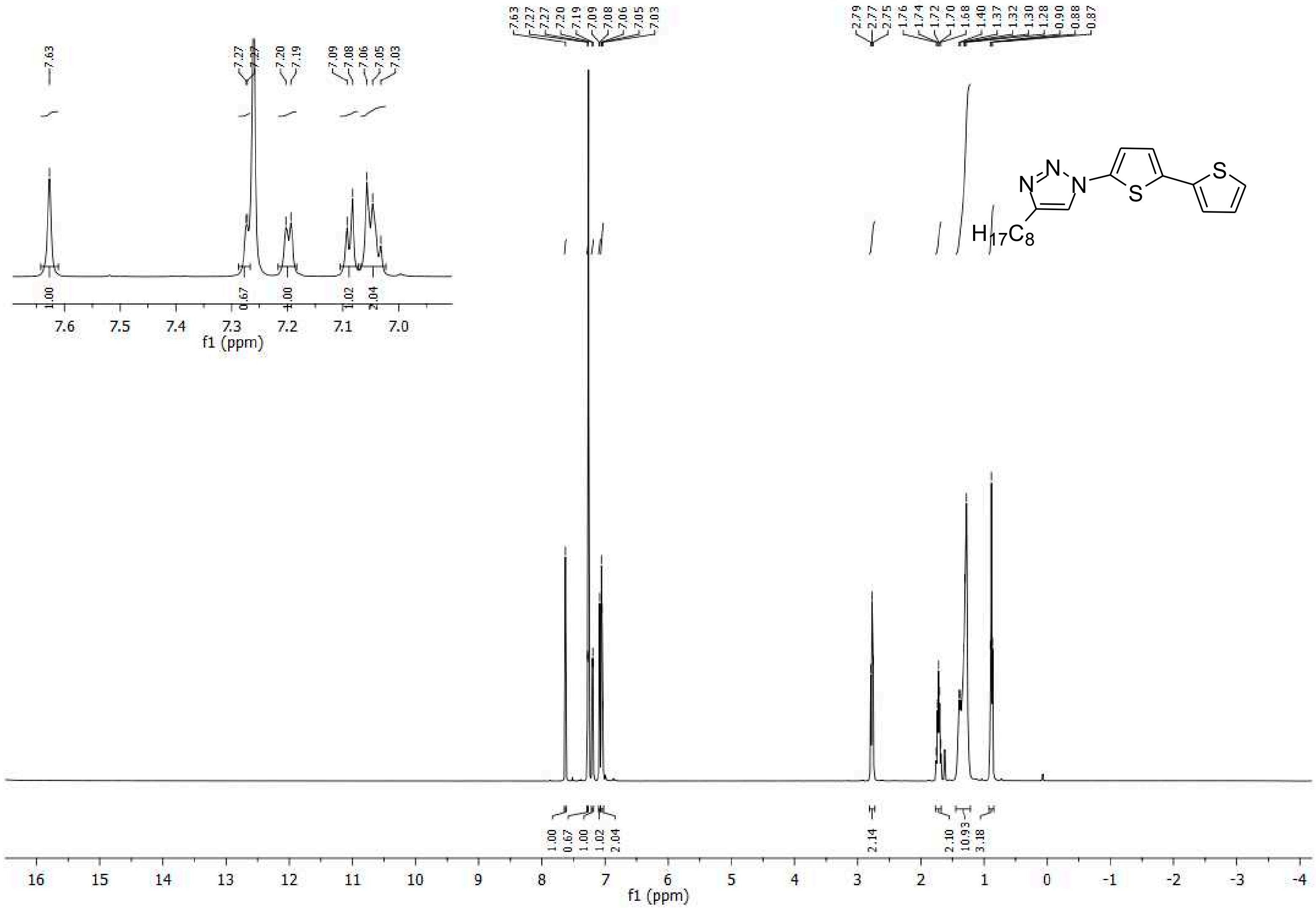

Figure S31. ^{1}H-NMR spectrum of compound (**22**).

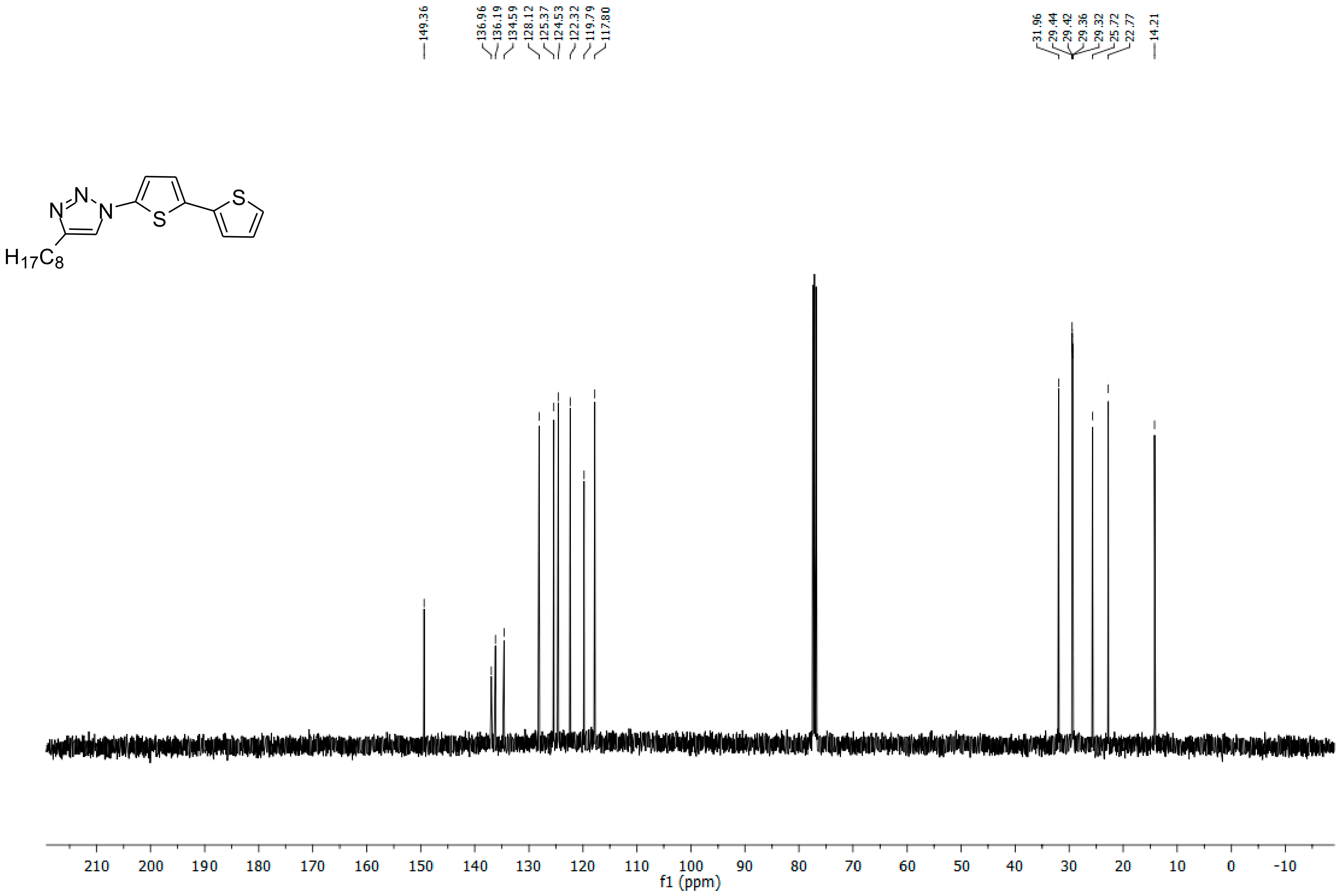

Figure S32. ^{13}C-NMR spectrum of compound (**22**).

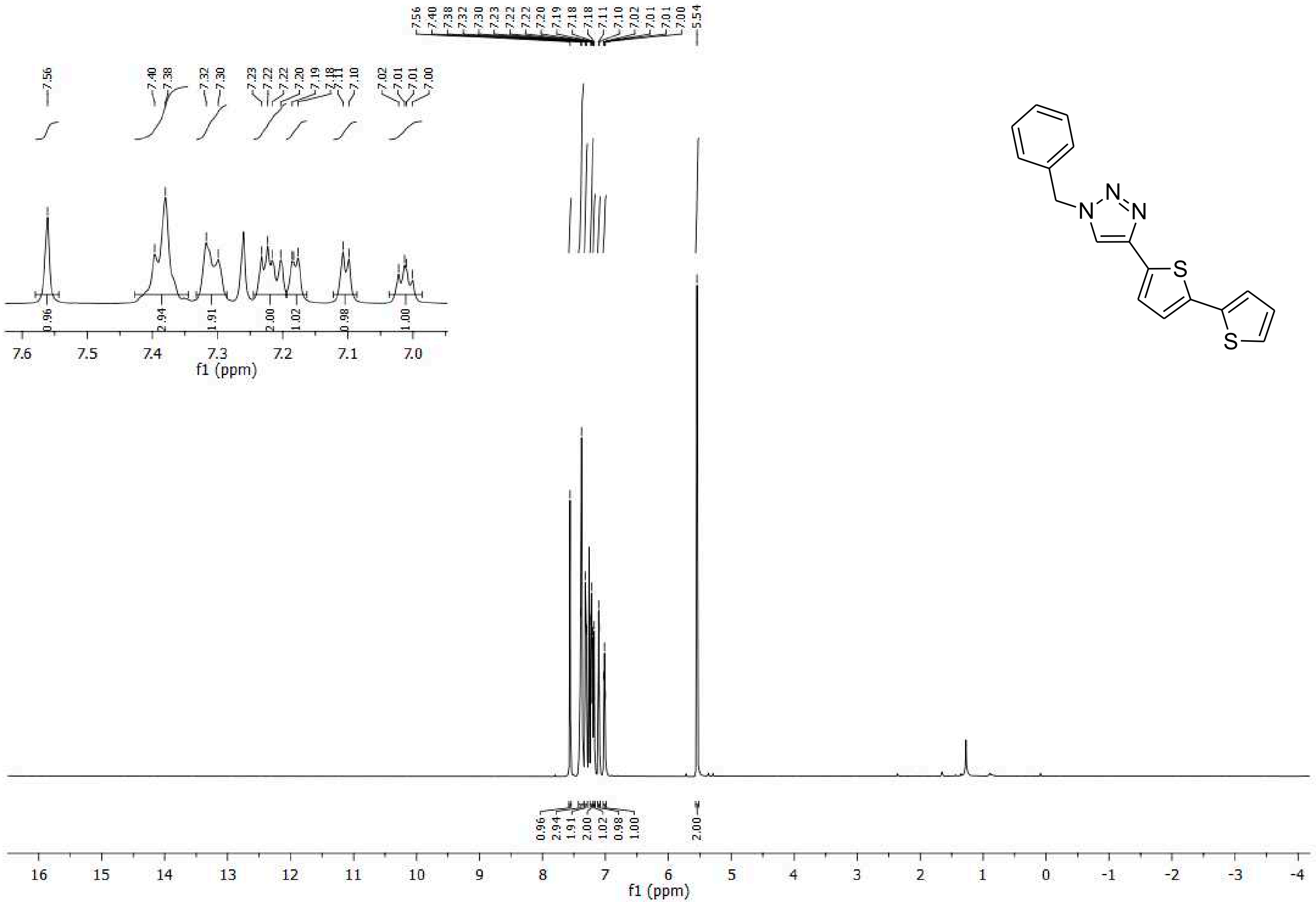

Figure S33. ^{1}H-NMR spectrum of compound (**23**).

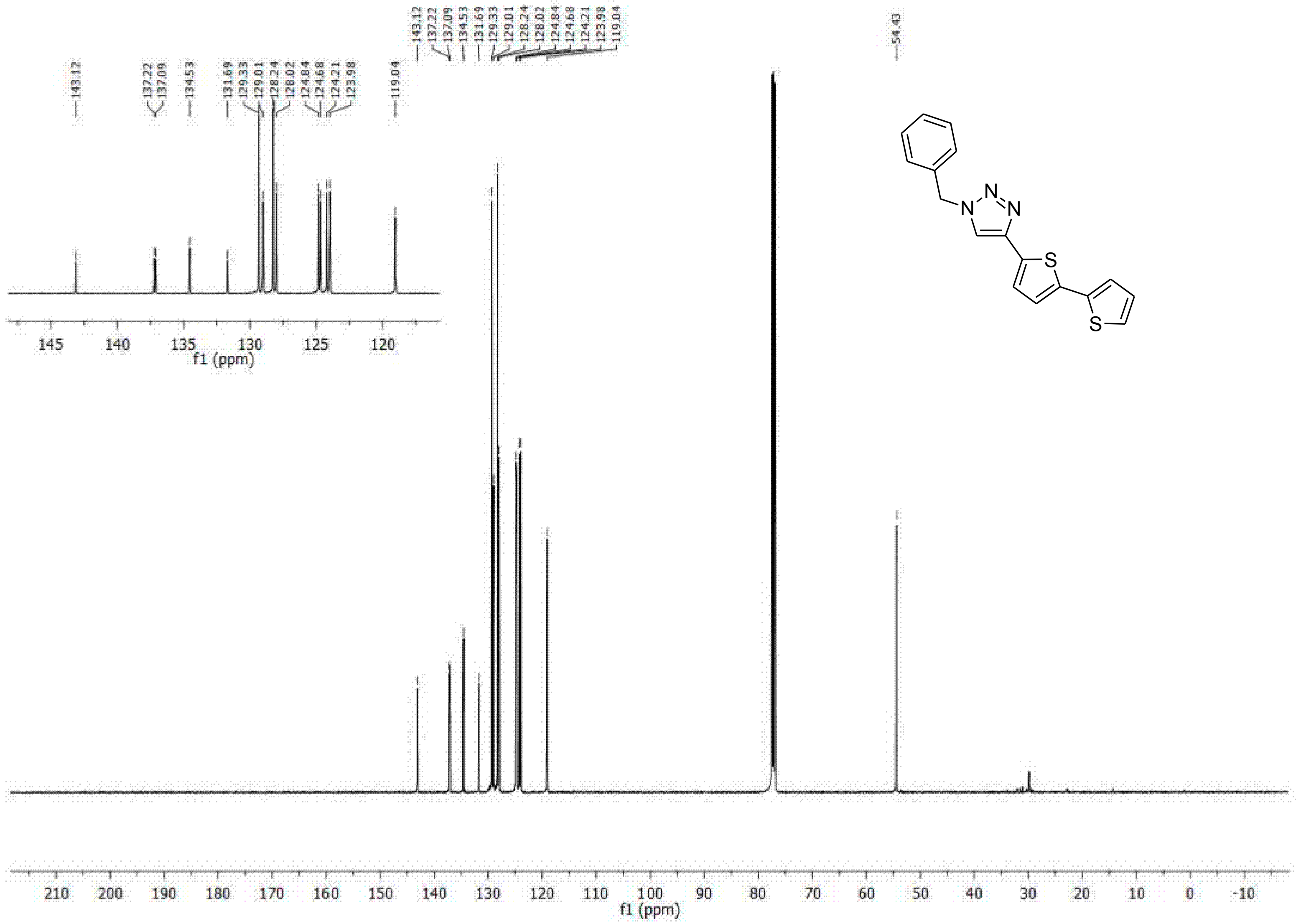

Figure S34. ^{13}C-NMR spectrum of compound (**23**).

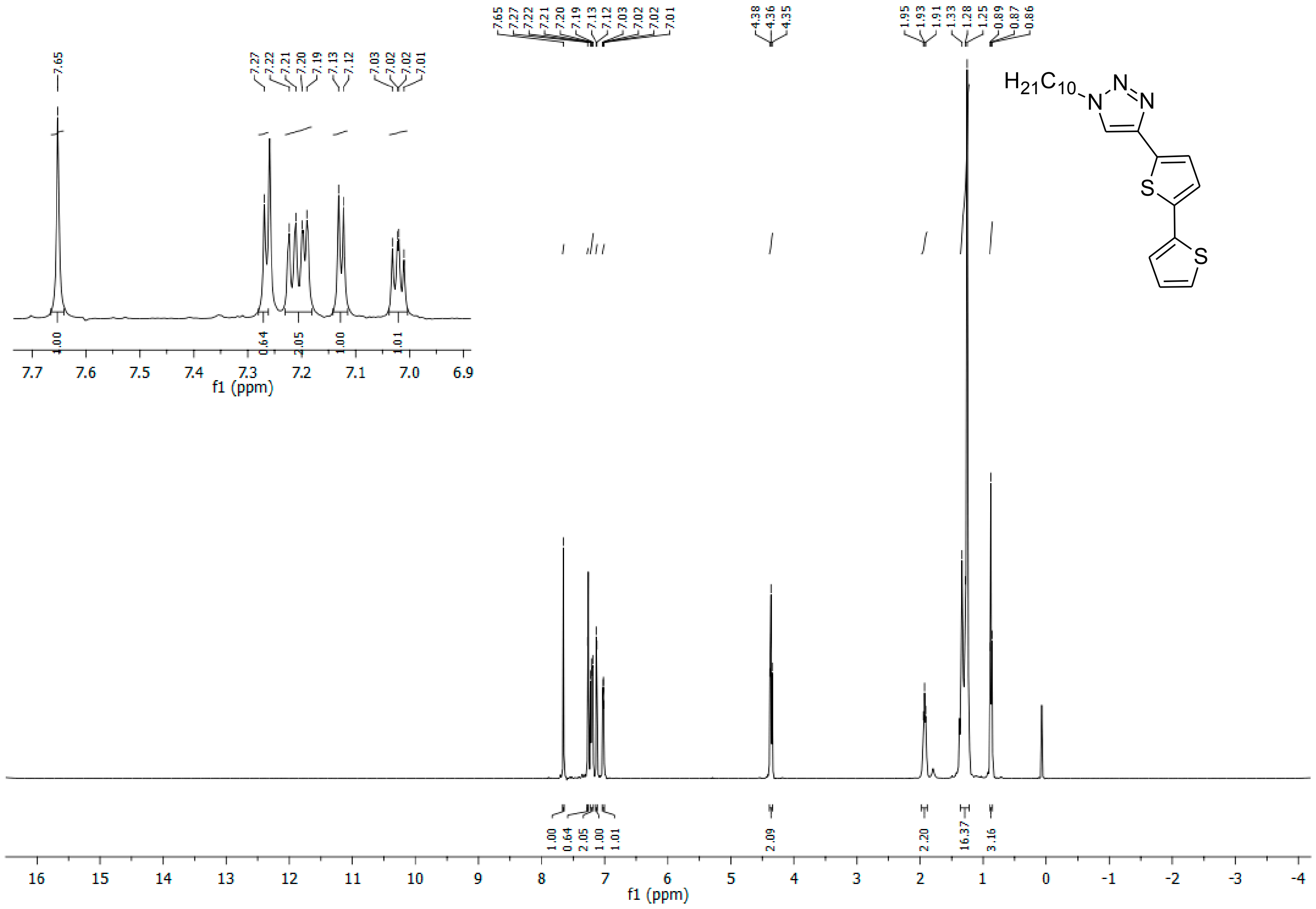

Figure S35. ^{1}H-NMR spectrum of compound (**24**).

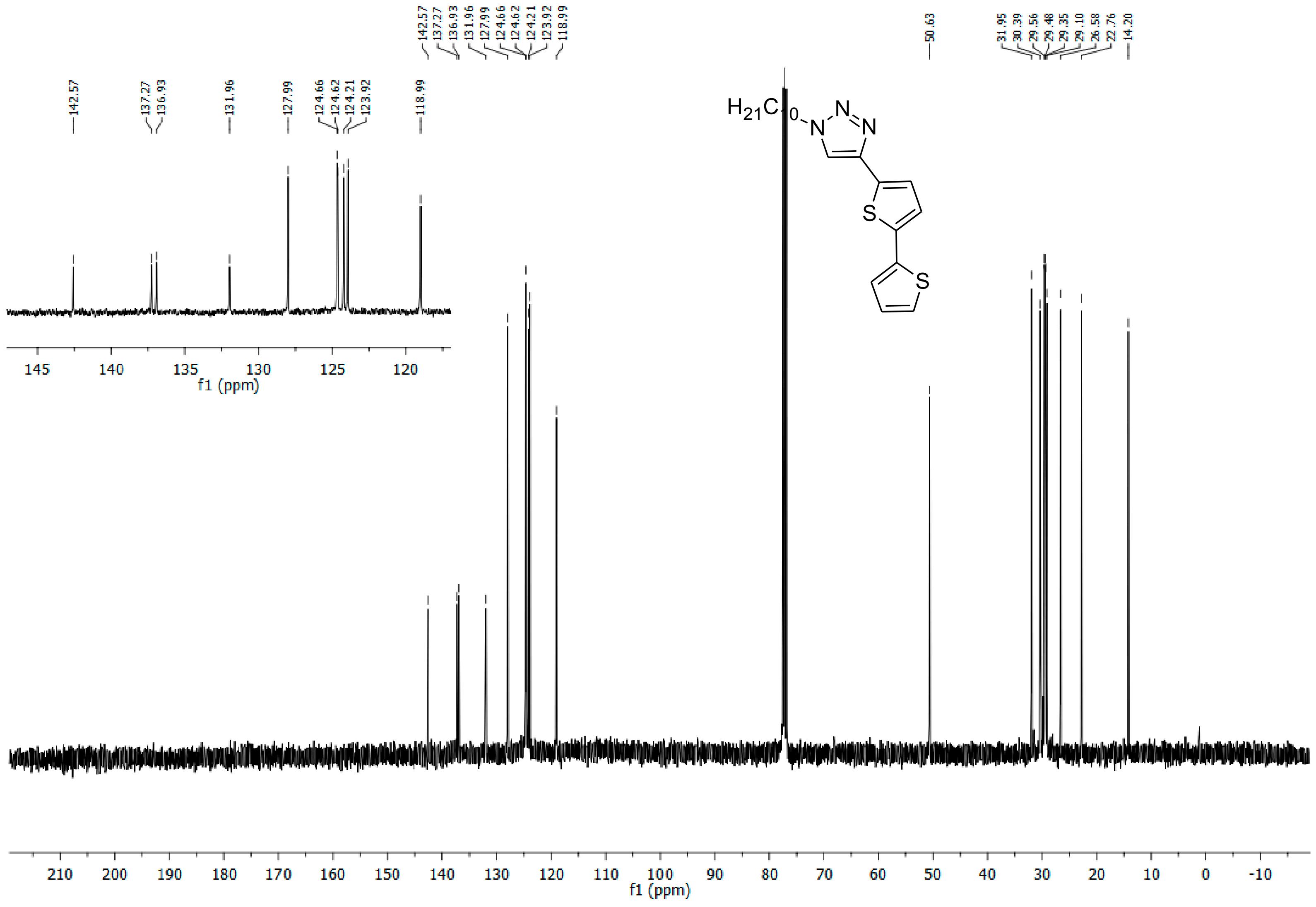

Figure S36. ^{13}C-NMR spectrum of compound (**24**).

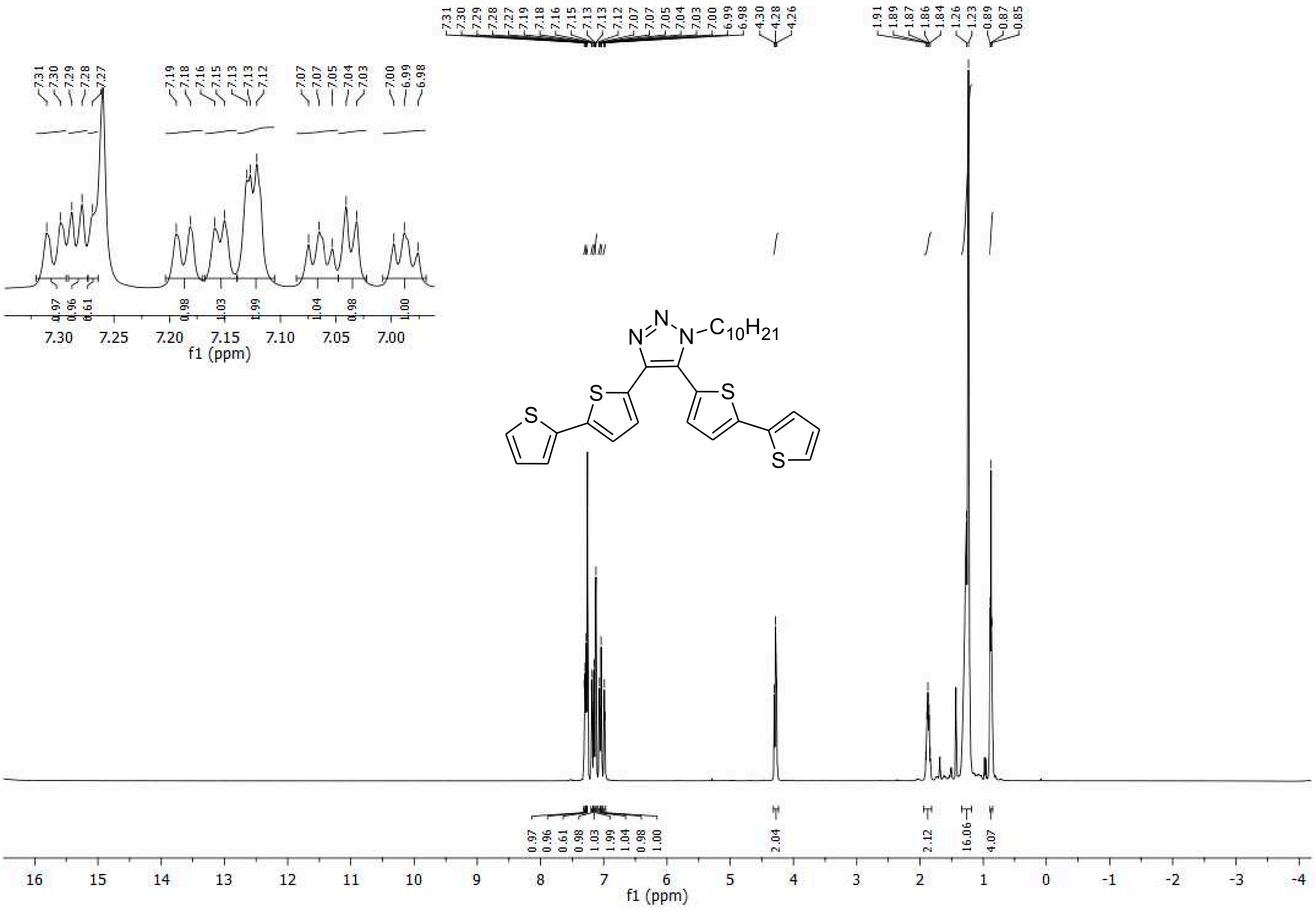

Figure S37. ^{1}H-NMR spectrum of compound (**25**).

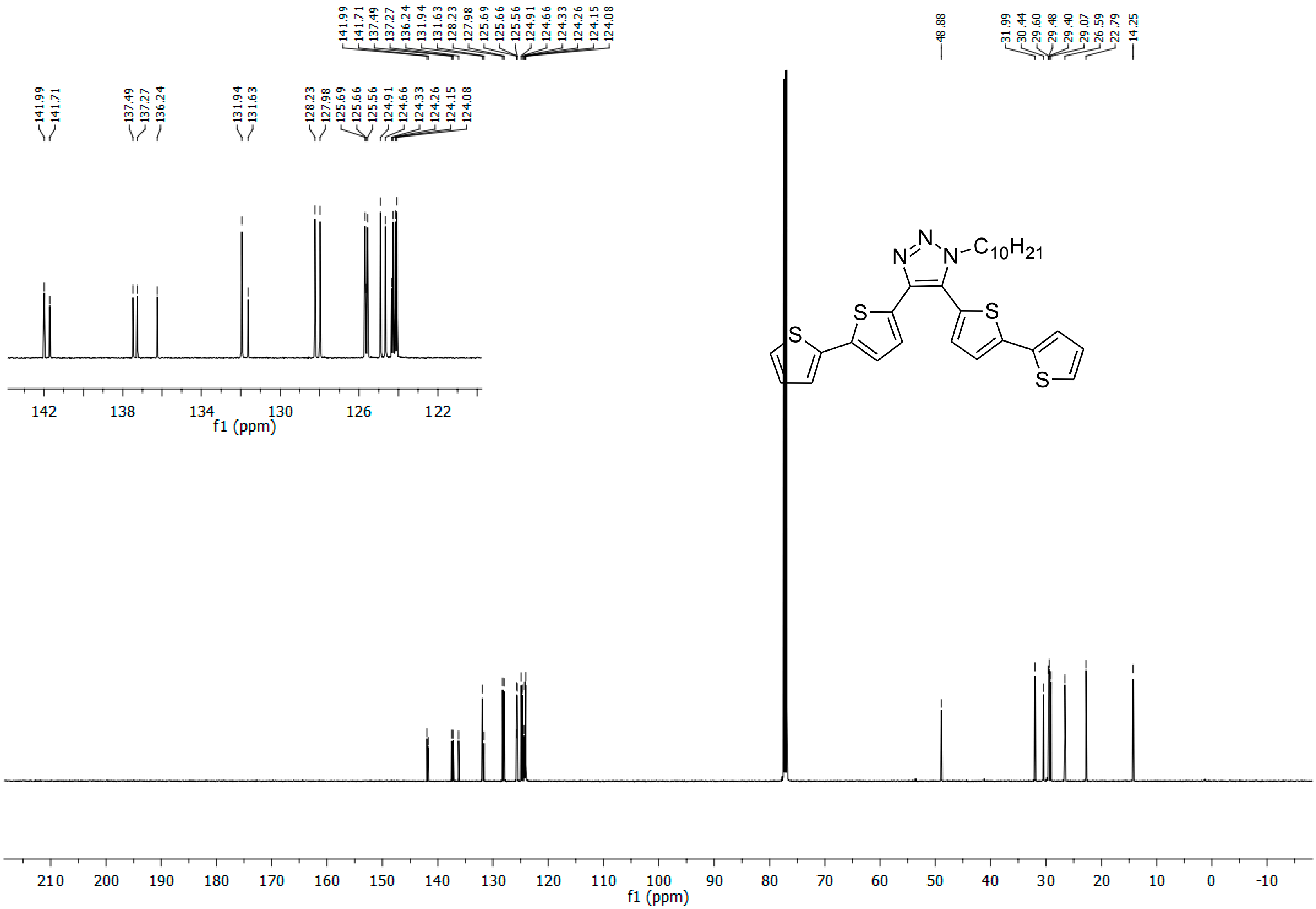

Figure S38. ^{13}C-NMR spectrum of compound (**25**).

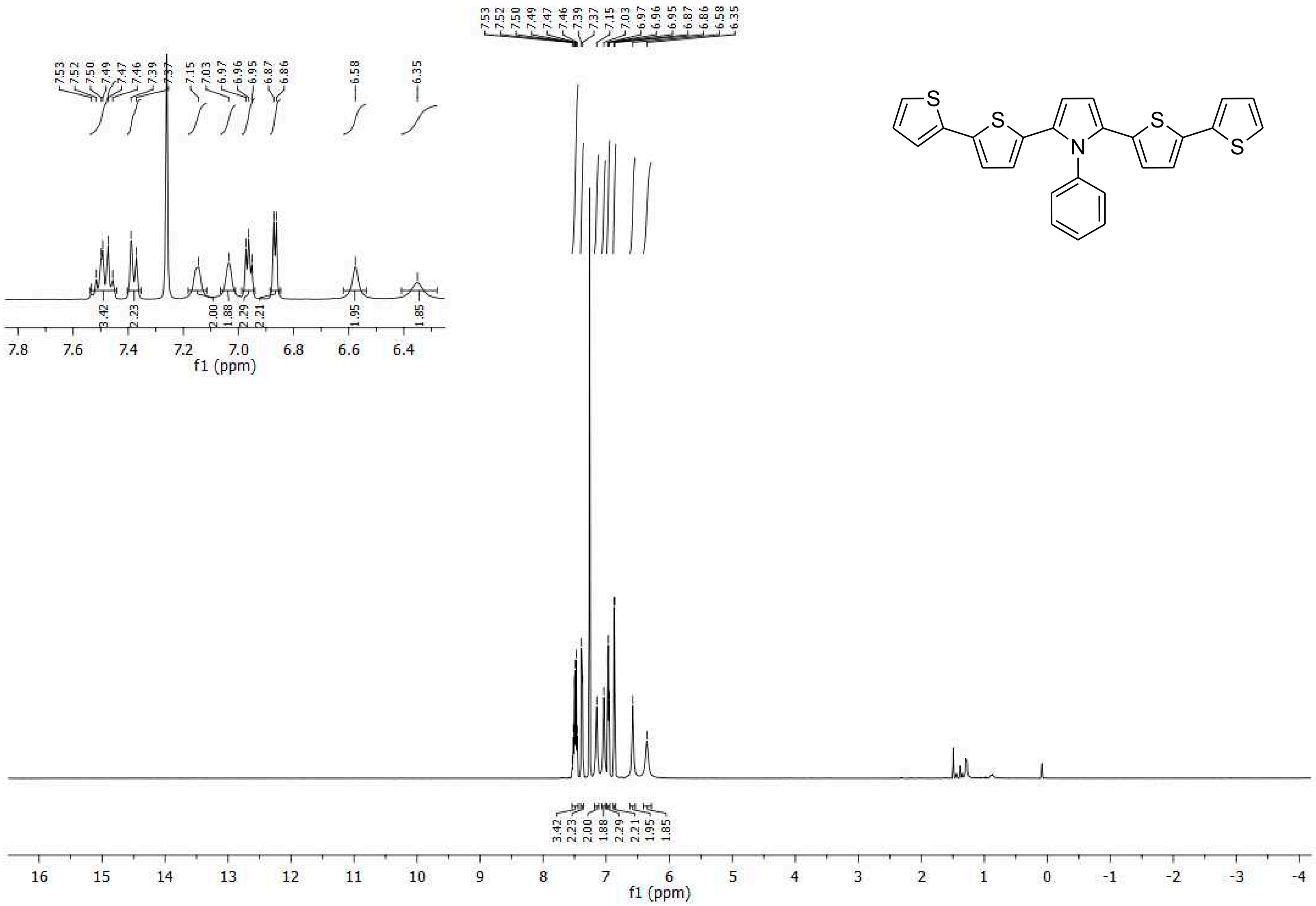

Figure S39. ^{1}H-NMR spectrum of compound (**26**).

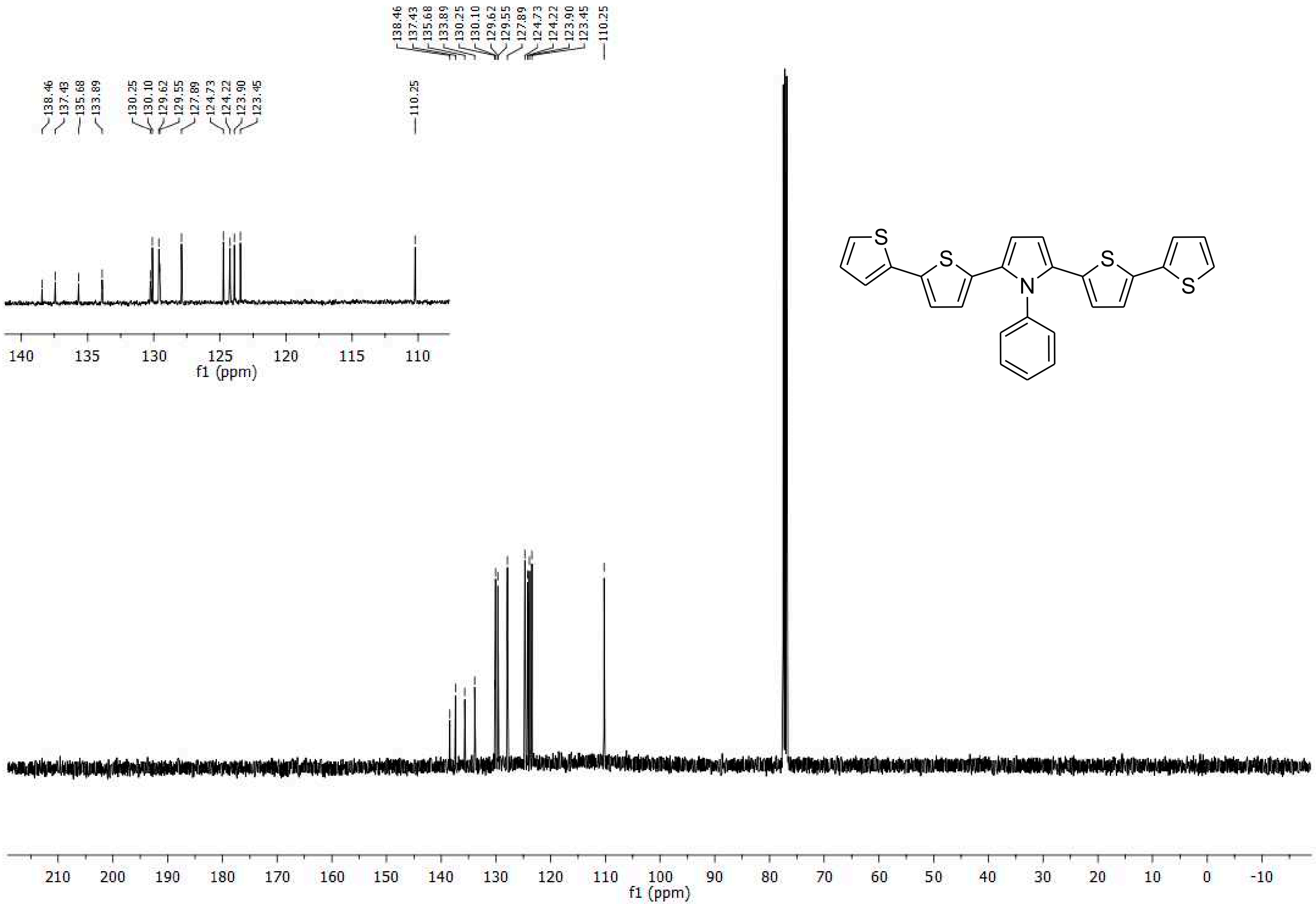

Figure S40. ^{13}C-NMR spectrum of compound (**26**).

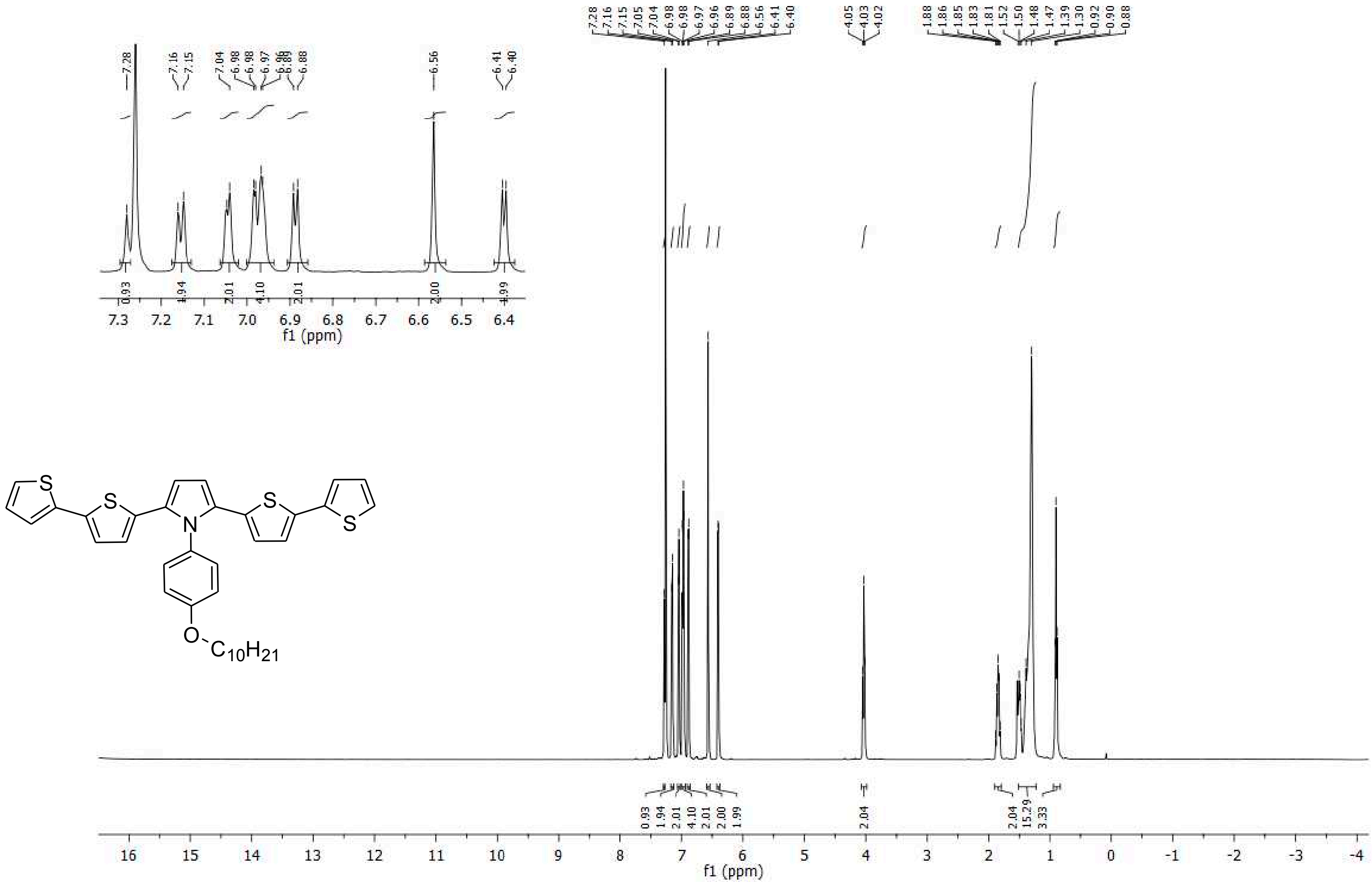

Figure S41. ^{1}H-NMR spectrum of compound (**27**).

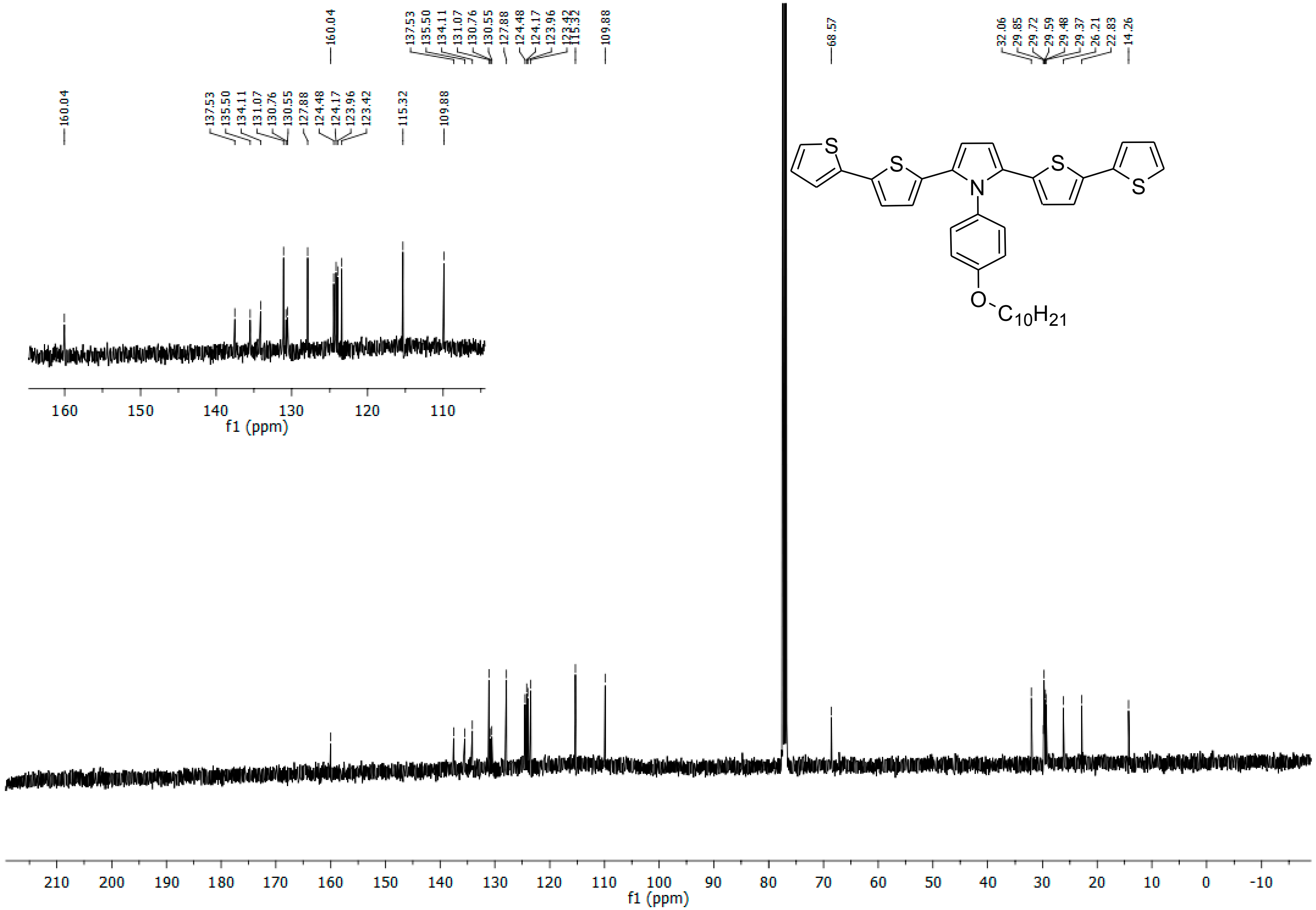

Figure S42. ^{13}C-NMR spectrum of compound (**27**).

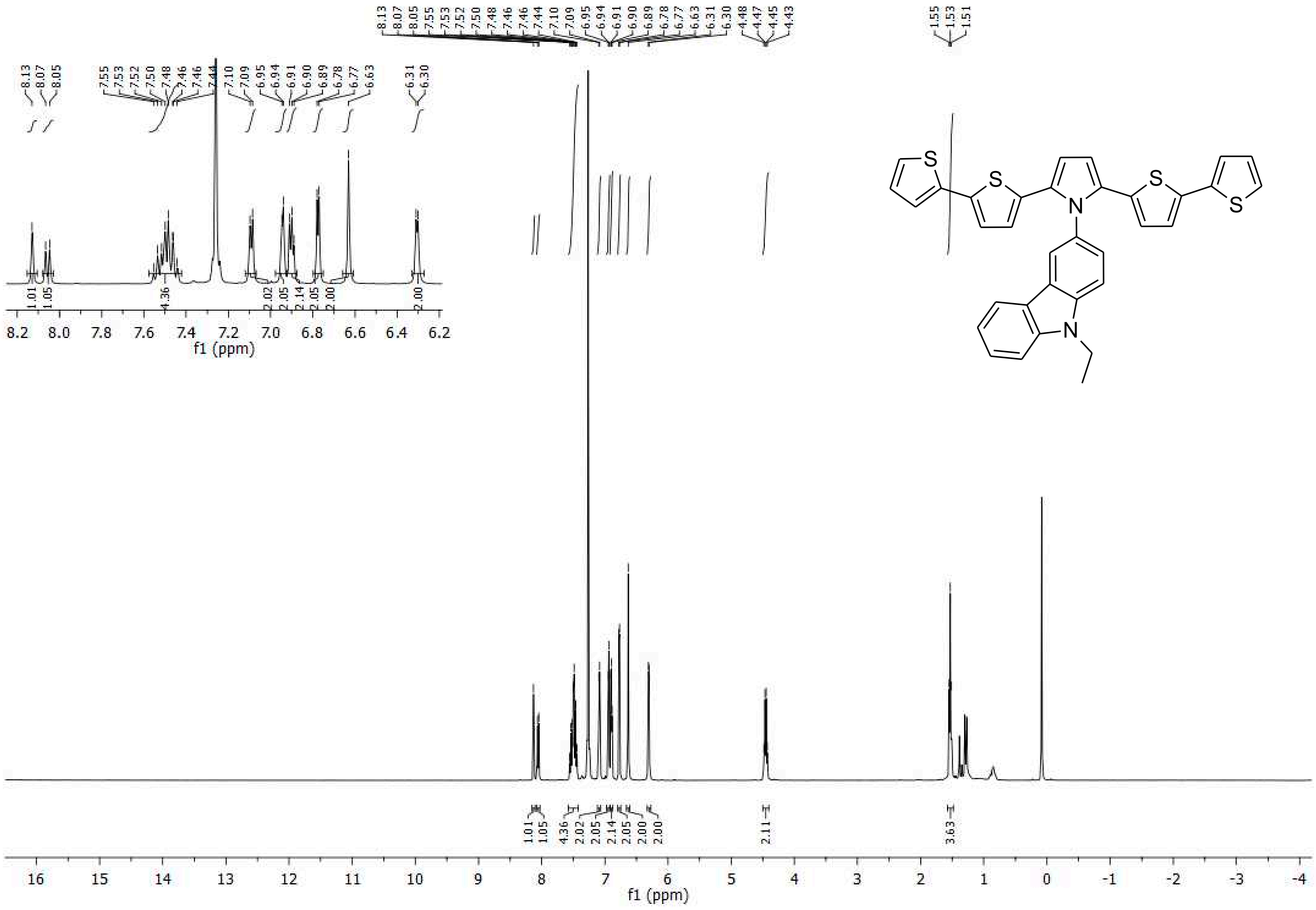

Figure S43. ^{1}H-NMR spectrum of compound (**28**).

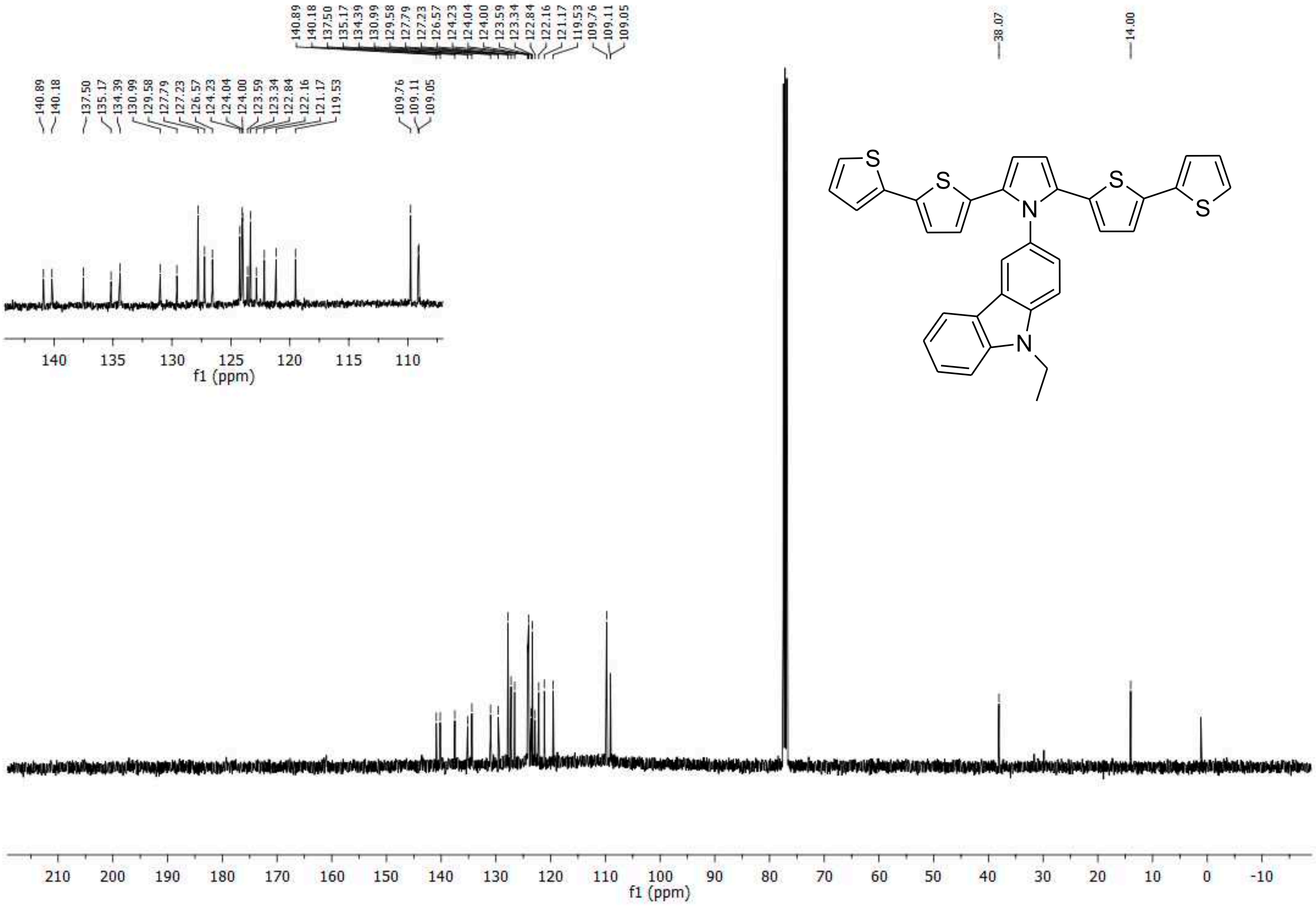

Figure S44. ^{13}C-NMR spectrum of compound (**28**).

3. Cyclic Voltammogramms of Selected Products

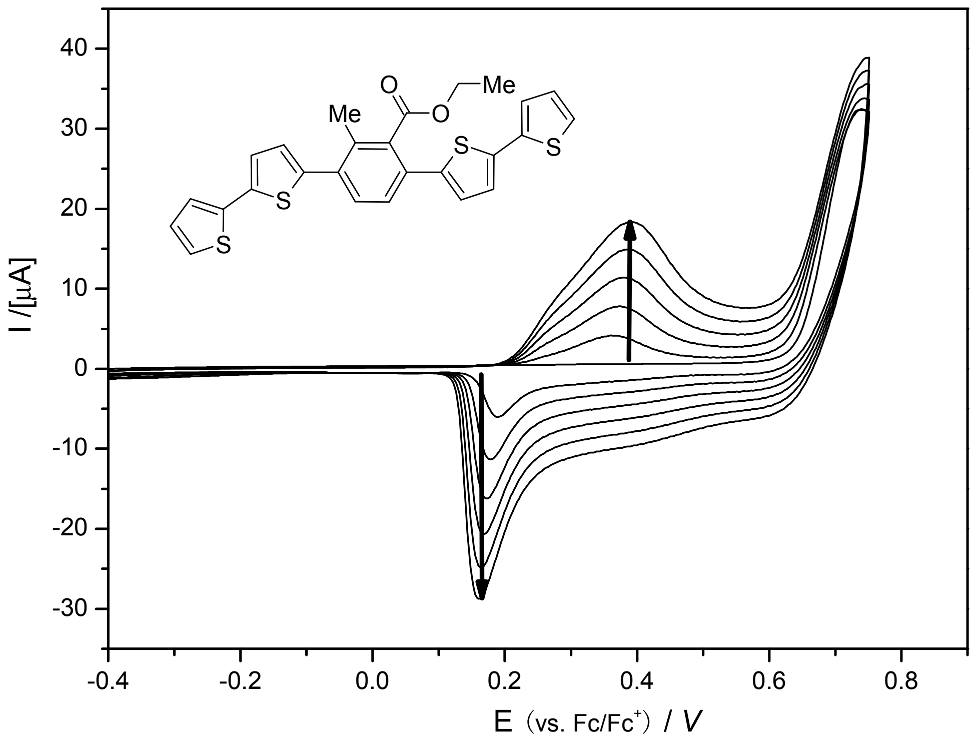

Figure S45. Cyclic voltammogramms of **7** obtained in the range of −0.4–0.75 V *vs*. Fc/Fc^+ on a platinum electrode; sweep rate ν = 100 mV/s, 0.2 mM in 0.1 M Bu4NPF6 in CH_2Cl_2.

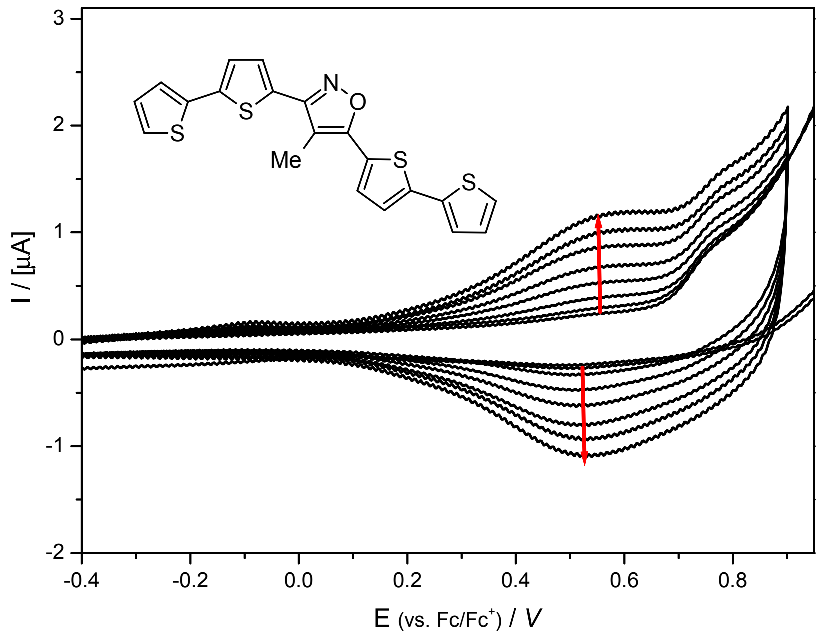

Figure S46. Cyclic voltammogramms of **17** obtained in the range of −0.4–0.95 V *vs*. Fc/Fc^+ on a platinum electrode; sweep rate ν = 100 mV/s, 0.2 mM in 0.1 M Bu4NPF6 in CH_2Cl_2.

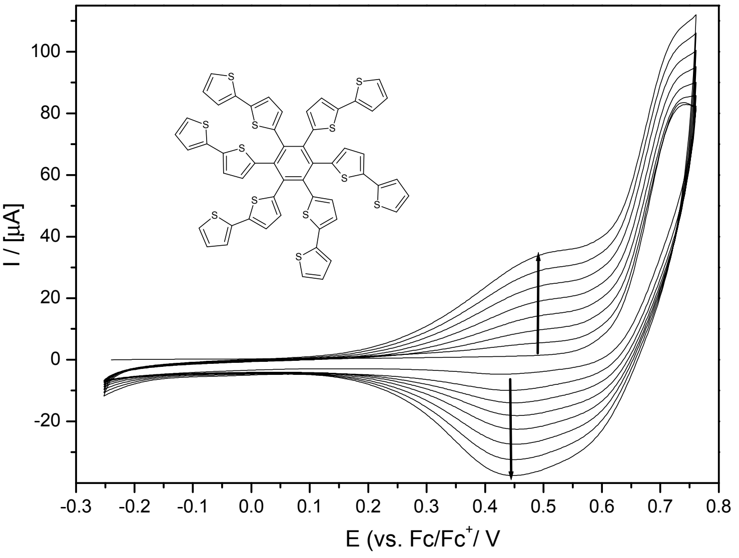

Figure S47. Cyclic voltammogramms of **21** obtained in the range of −0.25–0.75 V *vs.* Fc/Fc$^+$ on a platinum electrode; sweep rate ν = 100 mV/s, 0.2 mM in 0.1 M Bu4NPF6 in CH_2Cl_2.

4. Multistep Electrochromism of *poly-7* (Photos)

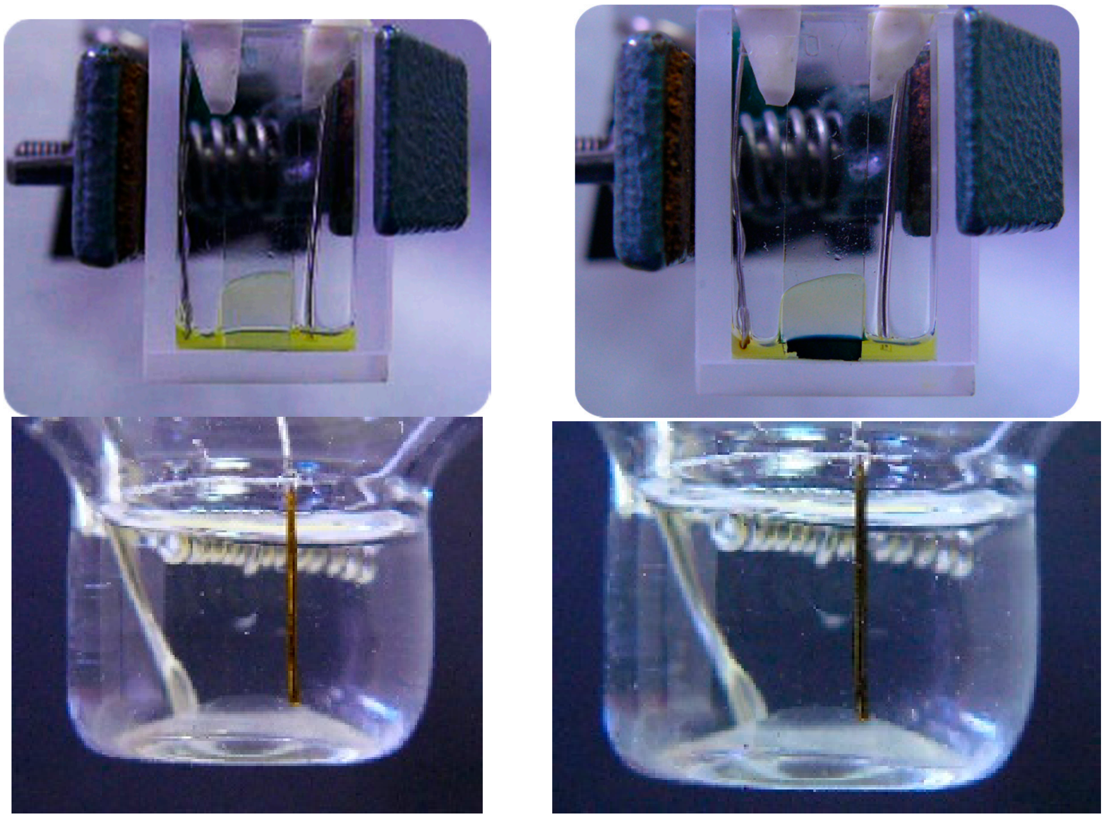

Photo S1. Multistep electrochromism of ***poly-7*** at: 0 V (top, left); 0.5 V (top, right); −1.9 V (immediately after oxidation, bottom, left); −1.9 V (bottom, right). ***Poly-7*** on ITO (top) or Pt (bottom) electrode in 0.1 M Bu4NPF6 in CH_2Cl_2.

5. Photovoltaic Performances of BHJ Solar Cells

Solar cells were fabricated on an indium tin oxide (ITO)-coated glass substrate with the structure ITO/PEDOT:PSS/P3HT:PCBM:26 (27)/Al, in air atmosphere. PEDOT:PSS was spin cast (5000 turns per minute, 25 sec.) from aqueous solution to form a film on the ITO substrate and placed in oven and annealed at the temperature of 130 °C for 10 min. A solution containing a mixture of P3HT:PCBM:26 (or 27) in chloroform solution with weight ratio 1:0.7:0.09 was then spin cast on top of the PEDOT:PSS layer. For thermal annealing, the blend films were placed in oven and annealed at the temperature of 130 °C for 30 min, before the deposition of Al electrode. Then, an aluminum electrode was deposited by thermal evaporation in a vacuum of about 5×10^{-5} Torr. Current density–voltage (J–U) characteristics of the devices were measured using a Solar Symulator Model SS100AAA with AM 1.5G. For solar cell performance a xenon lamp with an irradiation intensity of 100 mW/cm^2 was used. The area of one photovoltaic pixel was about 4.5 mm^2.

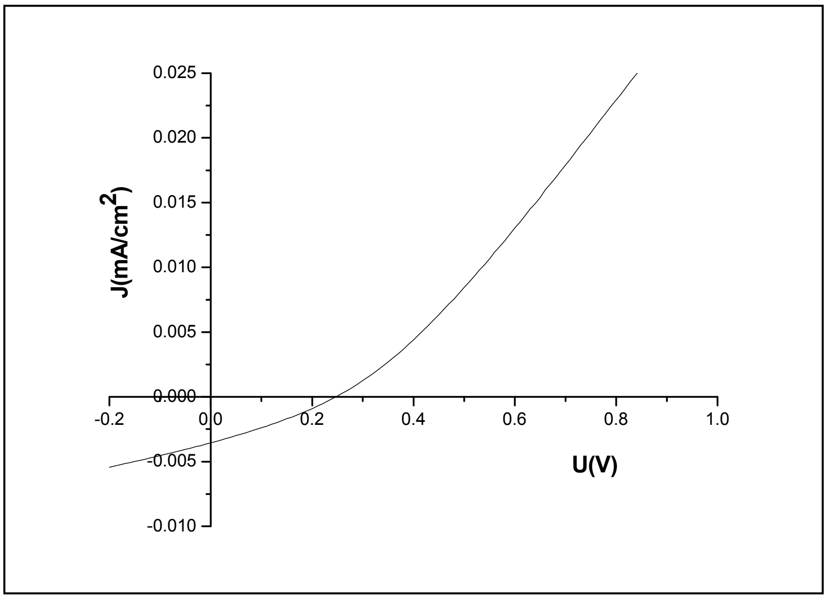

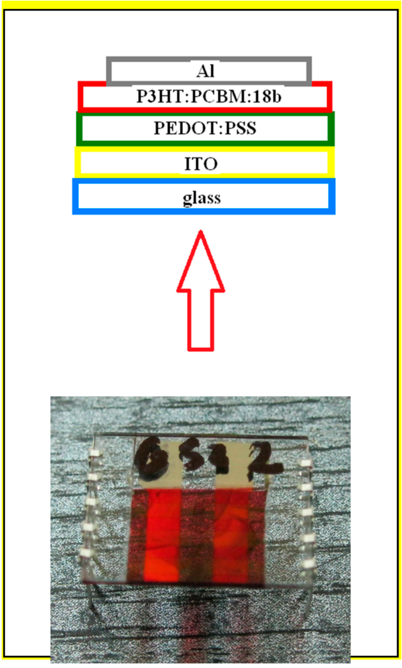

Figure S48. J–U curve of solar cell with active layer P3HT:PCBM:(**27**) under illumination 100 mW/cm^2 along with architecture and picture of constructed polymer solar cell with (**27**).

6. Novel 2,2'-Bithiophene Derivatives In Organic Electronics–Preliminary Examination

The series of novel compounds with 1–6 bt moieties was designed to be apply as materials in organic electronics. It was shown that some of them possess interesting properties. They can be considered as the precursors of a new conducting polythiophenes and compounds showing attractive luminescent properties. Thus, electrochemically obtained ***poly*-17** is the first ever reported example of polythiophene which contains isoxazole ring in conduction band (Figure S46). Obtained polymer ***poly*-17** was stable in multiple p-doping cycles. It is worth noting, that for (***poly*-17**) the bithienyl substituents in 1,3 positions to each other did not influence negatively on conductivity as in case of 1,3-disubstituted benzene derivatives. "Cutting of the π-conjugation" phenomena for 1,3-disubstituted

benzene derivatives, which is described in the literature, results in a significant increase of energy gap and oxidation potential. In the case of ***poly*-17**, this effect was not observed, indicating that the electrochemical properties of benzene and isoxazole derivatives (generally five- and six-membered hetero- and carboaromatic rings) cannot be directly compared. The electropolymerization of **7** is presented and its voltamogramm is shown in SI (Figure S45). The obtained ***poly*-7** is stable during p- and n-doping. Moreover, it shows a multistep electrochromism giving various colors: dark gold, blue, yellow and dark green, depending on the applied potential (Photo S1). In addition, compound **21** turned out to be a very interesting monomer as well (Figure S47).

However, the easiness of polymerization and formation of stable polymer during p-doping suggests, that in the electrochemical term a properties of this molecule are similar to those for 1,4-disubstituted benzene, like as for **7**. Presumably, in this case, a charge is stabilized based on toroidal delocalization—as it was similarly observed for hexa-substituted benzene derivative containing two thiophene moieties (in 1,4 or 1,2 positions) and four phenyl substituents in other positions. Moreover, compounds **26** and **27** were preliminary investigated as an additive in bulk heterojunction (BHJ) polymer solar cells based on poly(3-hexylthiophene-2,5-diyl) (P3HT) and [6,6]-phenyl C_{61} butyric acid methyl ester (PCBM). Photovoltaic properties were found for the BHJ devices with the architecture ITO/PEDOT:PSS/P3HT:PCBM:(**27**)/Al. The device with the active layer P3HT:PCBM: **27** showed an open circuit voltage U_{OC} of 0.24 V, a short circuit current density J_{SC} of 3.56 mA/cm^2, and a fill factor FF of 0.30, giving a power-conversion efficiency PCE of 0.25%. On the other hand, BHJ device with compound **14a** in active layer did not exhibit PV properties measured under 100 mW/cm^2 AM 1.5G solar illumination. Our study showed that chemical structure of additive in an active layer influence on the performance of polymer solar cells. The presented photovoltaic experiments suggested that pyrrole with bithiophene and p-decyloxyphenyl moieties **27** are better for the photovoltaic applications than compound **26** without p-decyloxyphenyl chain. J-U characteristic of the BHJ polymer solar cell with **27** along with the described method of fabrication of solar cells and the image of the constructed device are provided (Figure S48). Taking into consideration the present trends in the organic solar cells, it is very important and prosperous to apply new small organic compounds in PV to the control morphology of active layer and to investigate the intermolecular interactions and miscibility towards increase the performance of PV.
